# Exploring Calixarene‐Based Pseudorotaxane Architectures: Threading Ammonium Axles With an *Exo*‐Rim π‐Functionalized Calix[6]Arene Macrocycle

**DOI:** 10.1002/chem.71289

**Published:** 2026-06-23

**Authors:** Veronica Iuliano, Paolo Della Sala, Placido Neri, Donato De Simone, Rocco Del Regno, Margherita De Rosa, Carmine Gaeta, Carmen Talotta

**Affiliations:** ^1^ Dipartimento di Chimica e Biologia Università di Salerno Salerno Italy

**Keywords:** ammonium axles, calix[6]arenes, host–guest recognition, pseudorotaxanes, π‐functionalization

## Abstract

In this study, we present the synthesis of a novel 1,4‐π‐functionalized calix[6]arene derivative **2**, which features two electron‐rich 1,2,3‐trimethoxybenzene units on the *exo* rim and six methoxy groups on the *endo* rim. This design creates an extended and deep aromatic cavity, aimed at enhancing host–guest interactions with dialkylammonium axles. The results show that threading occurs even in the presence of bulky aromatic groups on the *exo* rim of **2**, observed with a series of symmetric and directional ammonium salts. NMR studies confirmed the formation of pseudorotaxane complexes and revealed that *exo*‐rim π‐functionalization influences binding affinity and orientational selectivity, depending on the axle's structure. Interestingly, the threading of **2** with the biphenyl axle **3c^+^** results in a pseudorotaxane stabilized by π···π interactions, which is 4.4 times more stable than the analog with hexamethoxy‐calix[6]arene **1a** that features *tert*‐butyl groups on the *exo* rim. Comparison of the *endo*‐*p*‐NO_2_‐benzyl/*endo*‐alkyl stereoselectivity ratios observed with directional axle **3e** ^+^ and the two macrocycles **1a** and **2**, indicates that the extended cavity of **2** enhances and promotes C─H···π interactions between the axles' alkyl chain and the enlarged aromatic cavity of **2**. This finding is further supported by density functional theory (DFT) and noncovalent interaction (NCI) calculations. The results described here provide valuable insights for designing new wheels with extended and deep aromatic cavities.

## Graphical abstract

This research presents a novel 1,4‐π‐functionalized calix[6]arene with enhanced interactions with dialkylammonium axles. We investigate threading behavior in CDCl_3_, highlighting how *exo*‐rim π‐functionalization affects binding affinity and stereoselectivity. Our findings, supported by DFT calculations, emphasize key noncovalent interactions (NCI) in stabilizing pseudorotaxane architectures.

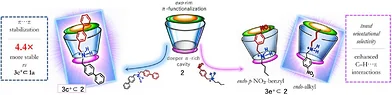

## 1 Introduction

The factors that govern and influence the formation of interpenetrated supramolecular architectures, such as pseudorotaxanes, continue to captivate scientists in the field of supramolecular chemistry [1]. The precise control over secondary chemical interactions that drive the templating effect for pseudorotaxane formation [2, 3] allows for the regulation of their thermodynamic stability and, at times, the control of the stereochemistry of the threading process [1, 4, 5, 6, 7, 8, 9, 10, 11, 12, 13, 14, 15, 16, 17, 18, 19, 20]. This aspect is critical in the template synthesis of mechanomolecules [1, 4−20].

Among the various macrocyclic wheels studied in the synthesis of mechanomolecules, calix[n]arenes (Figure 1) stand out for their peculiar structure, synthetic, and conformational versatility [1, 21, 22]. Compared to other macrocycles such as crown ethers [23], cyclodextrins [24], or cucurbiturils [25, 26], calixarenes enable fine manipulation of both rims and the introduction of functional groups that can significantly influence the stability of pseudorotaxanes and their stereochemistry [1, 21, 22].

**FIGURE 1:**
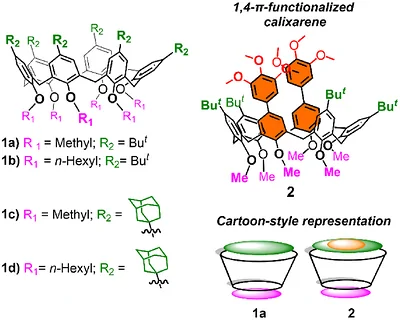
Structures of calix[6]arene wheels **1a**–**d** and derivative **2**.

Arduini and Secchi reported the first example of calix[6]arene threading with viologen axles [13, 14, 16, 18, 20, 21, 27]. Subsequently, Pappalardo, Parisi, and Gattuso demonstrated that the threading of the narrow annulus of calix[5]arene [28, 29]. Neri, Gaeta, and Talotta showed that scarcely pre‐organized calix[6]arene macrocycles (e.g., **1a**–**d** in Figure 1) are threaded with dialkylammonium axles (see cations in Figure 2), using the weakly coordinating Tetrakis[3,5‐bis(trifluoromethyl)phenyl]borate ([B(Ar^F^)_4_]^−^) counter anion (Figure 2) [30]. This anion facilitates efficient threading by generating “naked” cations in apolar media, thereby enabling stable pseudorotaxane formation even with conformationally flexible calix[6]arene hosts (e.g., **1a–d** in Figure 1) [30].

**FIGURE 2:**
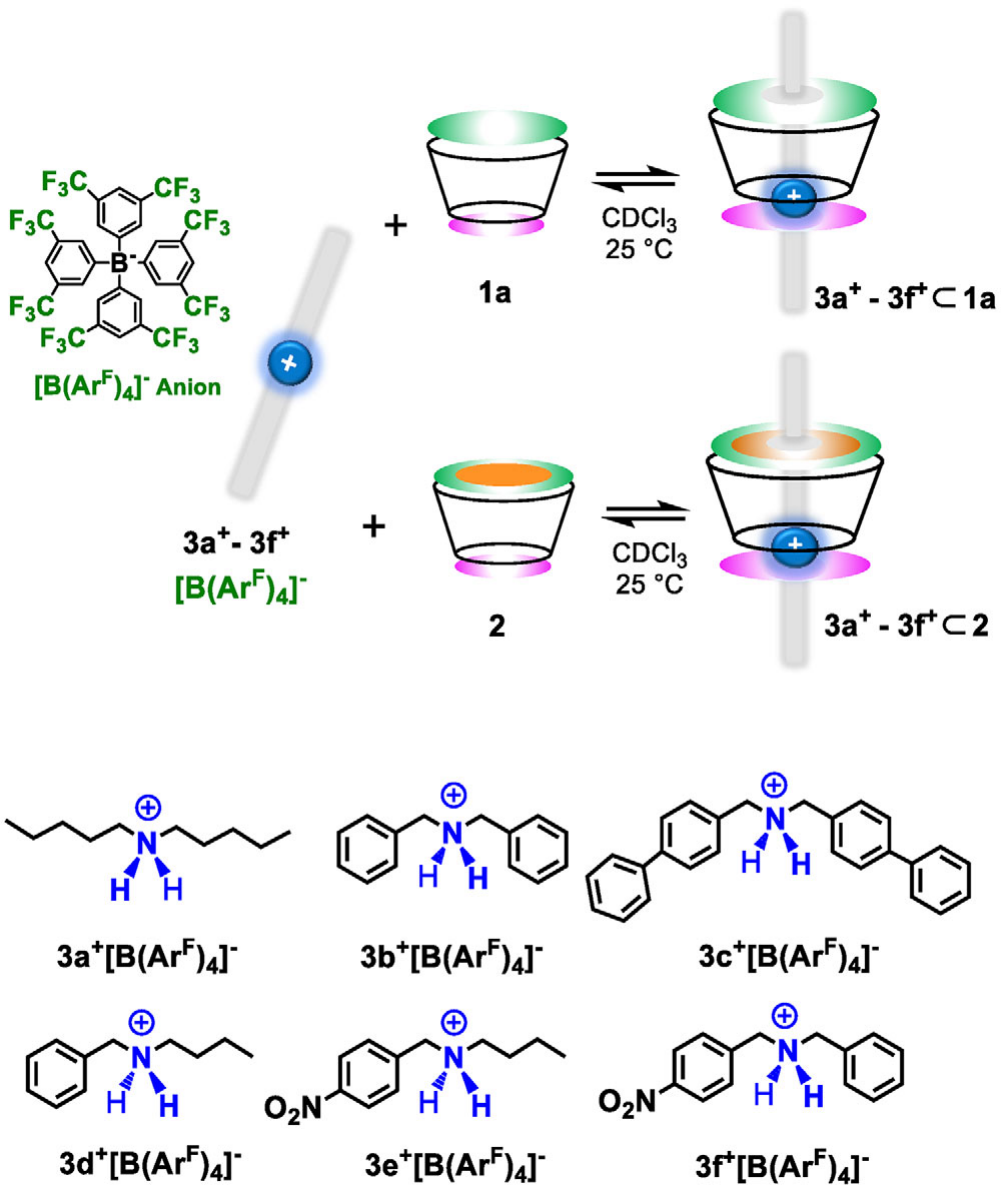
Cartoon representation of the threading equilibrium between calix[6]arene derivatives **1a** and **2** and dialkylammonium axles **3a** ^+^–**3f** ^+^, including the structure of the [B(Ar^F^)_4_]– anion.

Previously [31], our group demonstrated that the threading efficiency and thermodynamic stability of calix[6]arene‐based pseudorotaxanes can be significantly enhanced by incorporating large alkyl groups on both rims of the calixarene (e.g., **1c** and **1d** in Figure 1). In particular, calix[6]arene **1d** [31] functionalized with adamantyl groups on the *exo* rim and *n*‐hexyl chains on the *endo* rim, forms pseudorotaxane complexes with ammonium axles **3a** ^+^, **3b** ^+^, and **3d** ^+^ in Figure 1 that exhibit greater thermodynamic stability compared to hexamethoxy‐*p‐tert*‐butylcalix[6]arene **1a** [30]. This enhanced stability is attributed to the more favorable van der Waals interactions of adamantyl groups compared to *tert*‐butyl groups, with respect to the dicationic axle [31].

Based on these considerations, we were inspired to investigate the influence of aromatic units on the *exo* rims of the calix[6]arene skeleton (π‐functionalization). We envisioned that the introduction of π‐electron‐rich 1,2,3‐trimethoxybenzene moieties on the calix[6]arene *exo* rim could generate a π‐electron‐rich aromatic cavity that is deeper and provides a more extensive contact area with the ammonium axle. To further elucidate the role of electron‐rich aromatic groups at the *exo* rim, we designed a new calix[6]arene derivative **2**, rationally constructed to incorporate two key structural elements: (i) an extended π‐rich aromatic substituent at the upper rim to enhance potential π–π or CH–π interactions with the axle, and (ii) a fully alkylated lower rim. This dual‐functional approach aims to reinforce both electronic and steric contributions to host–guest recognition in pseudorotaxane complexes. Consequently, we proposed the synthesis and study of the threading abilities of a deep‐cavity calix[6]arene macrocycle **2** bearing these features.

## 2 Results and Discussion

### 2.1 Synthesis of the 1,4‐π‐Functionalized Calix[6]Arene Derivative **2**

The novel 1,4‐π‐functionalized calix[6]arene derivative **2** was obtained by the selective introduction of 1,2,3‐trimethoxybenzene moieties at positions 1 and 4 of the macrocycle, following the synthetic route outlined in Scheme 1. In calixarene chemistry, a commonly used approach for selectively functionalizing the upper rim exploits the decreased reactivity of alkylated PhOR rings relative to their unsubstituted phenol (PhOH) counterparts in partially derivatized compounds [32]. This essentially means that the selective derivatization initially applied to the *endo*‐rim is effectively shifted to the *exo*‐rim [32]. Following this approach, we applied a three‐step protection‐deprotection procedure, as outlined in Scheme 1, that yielded a 1,2,4,5‐tetramethylated calix[6]arene **6** bearing two 1,4‐diametrically positioned residual OH groups, achieving an overall yield of 40% [33]. *p‐tert*‐Butylcalix[6]arene **1e** was selectively 1,4‐dibenzylated [33] at the *endo*‐rim phenolic OH groups, yielding a partially protected intermediate **4** with a 60% yield. Subsequently, methylation of the free phenolic OH groups of **4** using methyl iodide and K_2_CO_3_ in acetone produced the fully alkylated derivative **5** in 96% yield. Catalytic hydrogenolysis (Pd/C, H_2_, and MeOH) of the benzyl protecting groups yielded compound **6**, which features two free phenolic OH groups at the *endo*‐rim (70% yield). 1,4‐Dihydroxycalix[6]arene **6** represents the ideal candidate for transferring the *endo*‐rim substitution pattern to the *exo*‐rim [32]. Consequently, selective bromination at the free phenolic rings of **6** with bromine in dry dichloromethane yielded the dibromo derivative **7** in 90% yield, which was subsequently methylated at the free OH groups on the *endo* rim to afford compound **8** in 98% yield.

**SCHEME 1:**
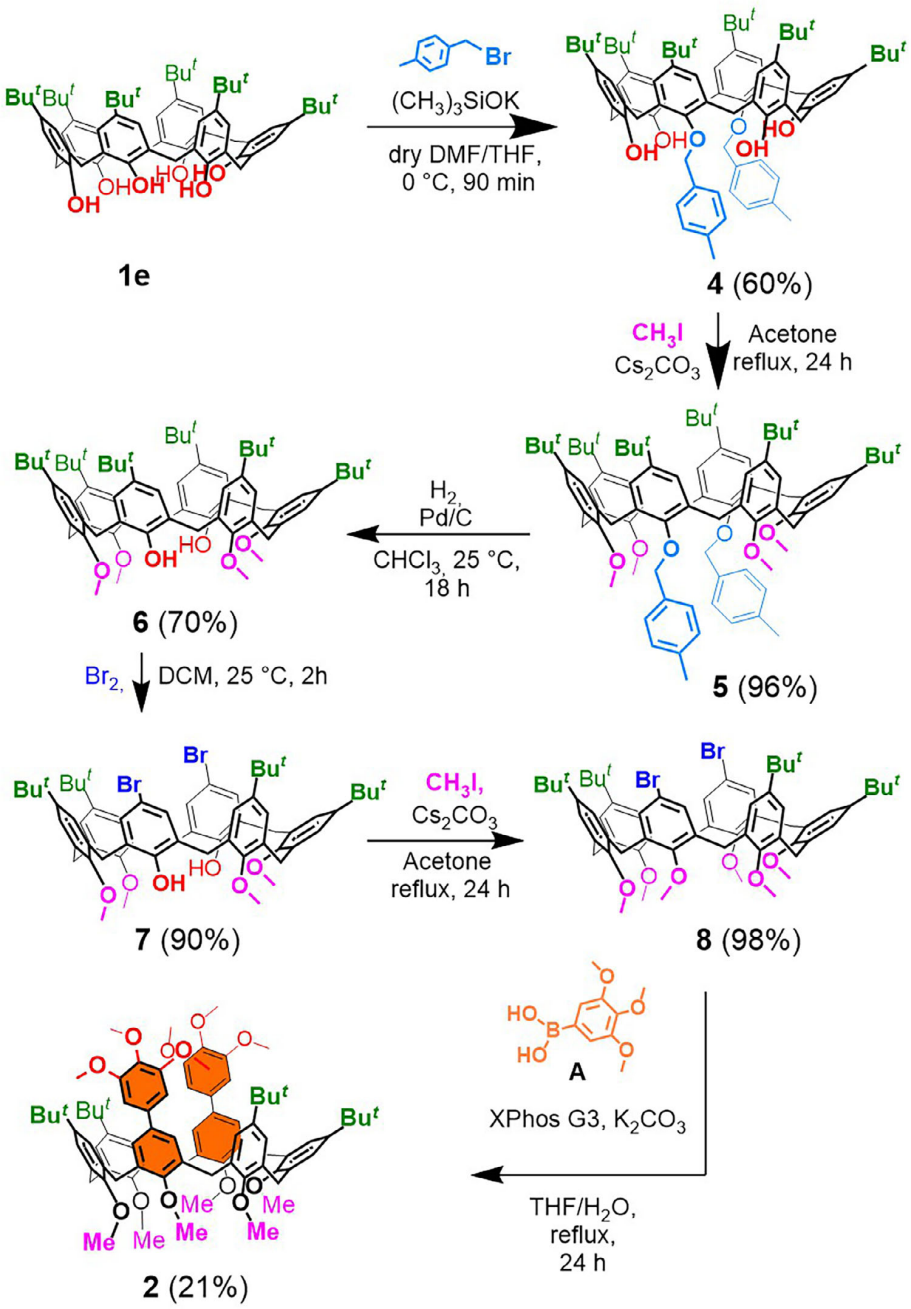
Synthesis of 1,4‐π‐functionalized calix[6]arene **2**.

The key transformation for obtaining selective 1,4‐π‐functionalization, involved Suzuki–Miyaura cross‐coupling between compound **8** and arylboronic acid **A**, utilizing XPhos Pd G3 as a catalyst. This reaction enabled the incorporation of two electron‐rich, π‐extended aryl substituents at the *exo*‐rim under mild conditions (THF/H_2_O, 80°C), resulting in the target derivative **2** with a yield of 21% after chromatographic purification.

Derivative **2** was fully characterized by 1D and 2D NMR (600 MHz, CDCl_3_, 298 K) and high‐resolution mass spectrometry (HR‐MS) (see Supporting Information). The ^1^H NMR spectrum (Figure 3a) reveals two singlets at 2.91 and 3.40 ppm (total of 18 hydrogen atoms, 2:1 ratio), associated with the methoxy groups at the *endo‐*rim. Additionally, two sharp singlets at 3.83 and 3.86 ppm (total of 18 hydrogen atoms, 2:1 ratio) correspond to the methoxy groups on the upper‐rim. In the ArCH_2_Ar region, two singlets at 3.92 and 3.97 ppm (total of 12 hydrogen atoms, 1:2 ratio) were observed. In the aromatic region, a signal at 6.56 ppm is attributed to the proton of the trimethoxybenzene moieties, while three additional signals at 6.89, 7.12, and 7.14 ppm (1:1:1 ratio) are assigned to the aromatic protons of the calixarene core. In addition, HR‐MALDI MS showed a molecular ion signal at *m/z* 1276.7217, in close agreement with the calculated value of 1276.7209 for **2** (C_82_H_100_O_12_).

**FIGURE 3:**
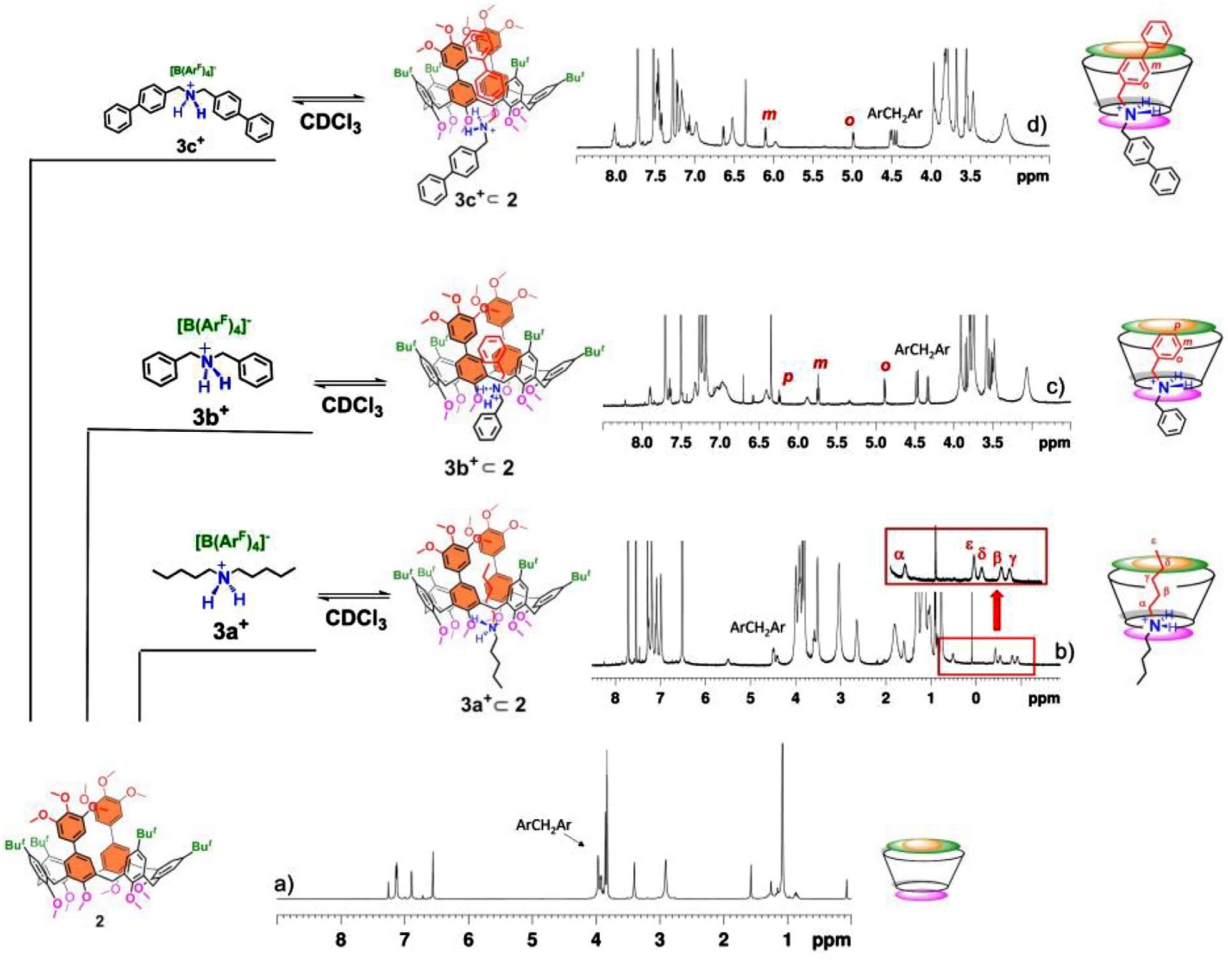
^1^H NMR spectra (600 MHz, CDCl_3_, 298 K) of: (a) free calix[6]arene **2**, and 1:1 mixture of **2** with the symmetric dialkylammonium axles, **3a^+^** (in b), **3b^+^** (in c), and **3c^+^** (in d). The appearance of diagnostic AX systems for the ArCH_2_Ar bridges, together with the shielding of the axle resonances included within the aromatic cavity, confirms the formation of the corresponding pseudo[2]rotaxanes **3a^+^** ⊂**2**, **3b^+^** ⊂**2**, and **3c^+^** ⊂**2**. The insert in (b) highlights the upfield‐shifted α–ε resonances of the threaded pentyl chain in **3a^+^** ⊂**2**.

### 2.2 Threading Studies With Constitutionally Symmetric Axles 3a^+^‒c^+^

We first investigated the threading behavior of derivative **2** with the dipentylammonium cation **3a^+^**. In the absence of a guest, calix[6]arene **2** exhibits sharp ^1^H NMR signals in CDCl_3_ at 298 K (Figure 3a), indicative of rapid *cone‐to‐cone* interconversion via *through‐the‐annulus* rotation of the OMe groups at the *endo* rim. Upon the addition of **3a^+^** (1:1 mixture of derivative **2** and **3a^+^** as a barfate salt in CDCl_3_), new resonances appeared in the ^1^H NMR spectrum (Figure 3b), confirming the formation of pseudorotaxane **3a^+^** ⊂**2** [30]. Two diagnostic AX systems for the ArCH_2_Ar groups emerged at 4.36 and 3.53 ppm (*J* = 13.0 Hz), and at 4.45 and 3.55 ppm (*J* = 12.3 Hz). These resonances indicate that, upon the formation of the pseudorotaxane **3a^+^** ⊂**2**, the calixarene wheel **2** adopts a cone conformation [34, 35, 36] that is stable on the NMR time scale. Additionally, four upfield‐shifted alkyl signals (from −0.42 to −0.92 ppm) further substantiate the inclusion of the axle **3a^+^** within the calixarene **2** cavity. Analysis of the COSY‐45 spectrum (SI) from a 1:1 mixture of derivative **2** and **3a** ^+^ in CDCl_3_ provided further confirmation of the pseudorotaxane structure, revealing two distinct sets of signals corresponding to the pentyl chains of the axle **3a** ^+^ [30]. One set is attributable to the pentyl chain embedded within the calixarene cavity, with *γ*, *β*,* δ, ε*, and *α* protons resonating at −0.92, −0.80, −0.53, −0.43, and 0.52 ppm, respectively. The other set corresponds to the pentyl chain extending outward from the narrow rim.

The threading of the axle **3a^+^** into derivative **2** reached equilibrium after mixing at 298 K, resulting in a 50% formation of the pseudorotaxane **3a^+^** ⊂**2**. The apparent association constant (*K*_a_) was determined to be (5.2 ± 0.2) × 10^2^ M^−^^1^, based on the direct integration of diagnostic NMR signals [30, 37] corresponding to the free and complexed species [30]. The binding constant of (5.2 ± 0.2) × 10^2^ M^−^^1^ calculated for the formation of the pseudorotaxane **3a^+^** ⊂**2** is higher than that reported for the formation of the pseudorotaxane with hexamethoxy‐*p‐tert*‐butylcalix[6]arene **3a^+^** ⊂**1a**, which is (4.4 ± 0.2) × 10^2^ M^−^^1^ [30]. This suggests that the extended cavity of **2**, due to the 1,4‐π‐functionalization with electron‐rich 1,2,3‐trimethoxybenzene units on the *exo* rim of calix[6]arene, establishes additional secondary interactions with the axle **3a^+^** (vide infra) and slightly enhances the thermodynamic stability of **3a^+^** ⊂**2** compared to **3a^+^** ⊂**1a**.

Additionally, *through‐the‐annulus* threading was observed between calix[6]arene derivative **2** and dibenzylammonium salt **3b^+^** in CDCl_3_. Upon the addition of **3b^+^** to the CDCl_3_ solution of **2** (in a 1:1 ratio), significant changes appeared in the ^1^H NMR spectrum of **2** (Figure 3c), indicating the formation of the pseudorotaxane **3b^+^⊂2**. The cone conformation of calix[6]arene **2** in **3b^+^⊂2** was confirmed through 1D and 2D NMR studies (see Supporting Information). Specifically, AX systems attributed to methylene bridge protons were observed at 3.52/4.36 ppm (*J* = 14.6 Hz) and 3.55/4.50 ppm (*J* = 14.3 Hz).

In the pseudorotaxane **3b** ^+^⊂**2**, the dibenzylammonium axle **3b** ^+^ exhibited two distinct benzylic ^1^H NMR resonance signals [30]. The ^1^H NMR signals at 4.93, 5.78, and 6.27 ppm (corresponding to *ortho*‐, *meta*‐, and *para*‐BnH, respectively) were attributable to the aromatic ring of **3b^+^** located inside the cavity of **2**, while the benzylic group of **3b^+^** located outside the calixarene cavity showed ^1^H NMR signals at typical chemical shift values (6.5–7.5 ppm). The threading equilibrium between **3b^+^** and **2** showed a kinetic slow compared to the NMR time scale; thus, the direct integration of the ^1^H NMR signals of free and complexed host **2** allowed for the determination of the apparent association constants. A binding constant value (*K*_a_ in Table 1) of (6.7 ± 0.2) × 10^2^ M^−^^1^ was calculated for the formation of the pseudorotaxane **3b** ^+^⊂**2**, which is lower than the value of (2.5 ± 0.2) × 10^3^ M^−^^1^ calculated for the formation of **3b** ^+^⊂**1a** [30]. The comparison of the threading abilities of the two macrocycles, **1a** and **2**, with the axles dipentylammonium **3a** ^+^ and dibenzylammonium **3b** ^+^ shows that the two trimethoxybenzene rings on the *exo*‐rim of **2** enhance its ability to thread with the dipentylammonium axle compared to **1a**. In contrast, the dibenzylammonium axle behaves differently. This difference is likely because the pentyl portion of **3a** ^+^ can form a large number of C─H··· π interactions with the deep aromatic cavity of **2** than with that of **1a**. Prompted by this result, we studied the threading of the biphenyl axle **3c** ^+^ as a barfate salt [38] with **2**. When a 1:1 mixture of **2** and **3c^+^** was combined in CDCl_3_ at 298 K, the ^1^H NMR spectrum of the mixture exhibited well‐defined resonances indicative of the formation of the pseudorotaxane **3c^+^** ⊂**2** (Figure 3d). The 2D COSY spectrum acquired immediately after mixing in CDCl_3_ revealed two distinct AX systems at 3.54/4.52 and 3.55/4.46 ppm, which correlated in the HSQC spectrum with ^13^C resonances at 28.1 and 28.8 ppm, indicative of methylene bridge groups between syn‐oriented aromatic rings [34, 35, 36, 38]. This result highlights the formation of a **3c^+^** ⊂**2** pseudorotaxane structure in which calix[6]arene **2** adopts a cone conformation. A closer analysis of the 1D and 2D NMR spectra of **3c** ^+^⊂**2** clearly identifies two aromatic AX systems at 5.00/6.11 ppm (*J* = 7.8 Hz) and 6.64/7.43 ppm (*J* = 7.6 Hz), which are assigned to the aromatic protons of axle **3c** ^+^ shielded within the calixarene cavity (Figure 3d). The apparent association constant was determined to be (3.8 ± 0.2) × 10^2^ M^−^^1^ (slow exchange in the NMR time scale). This value is 4.4 times higher than that measured for the formation of the pseudorotaxane **3c** ^+^⊂**1a**, of (87 ± 2) M^−^^1^, suggesting that the extended π‐rich substituents at the *exo*‐rim of **2** enhance host–guest stabilization through additional π—π stacking interactions with the biphenyl axle **3c** ^+^.

**TABLE 1:** Association Constants (*K*_a_, M^−1^) of dialkylammonium **3a** ^+^–**3f** ^+^ complexes with calixarene hosts **1a** and **2** determined by ^1^H NMR spectroscopy (600 MHz, CDCl_3_, 25°C) upon addition of 1 equiv. of their corresponding barfate salts.

Axle	*K*_a_ (M^−^^1^) 3a^+^‐3f^+^⊂1a	*K*_a_ (M^−^^1^) 3a^+^–3f^+^⊂2	Diastereoisomeric ratio for 1a	Diastereoisomeric ratio for 2
**3a^+^**	(4.4 ± 0.2) × 10^2^ ^a^	(5.2 ± 0.2) × 10^2^ ^c^	—	—
**3b^+^**	(2.5 ± 0.2) × 10^3^ ^a^	(6.7 ± 0.2) × 10^2^ ^b^	—	—
**3c^+^**	87 ± 2^b^	(3.8 ± 0.2) × 10^2^ ^b^	—	—
**3d^+^**	(6.5 ± 0.2) × 10^4^ ^d^	(2.1 ± 0.2) × 10^3^ ^c^	**90**/10 (***endo*‐alkyl**)**/**(*endo*‐benzyl)	**95**/5 (***endo*‐alkyl**)**/**(*endo*‐benzyl)
**3e^+^**	(8.3 ± 0.2) × 10^3^ ^b^	(9.3 ± 0.2) × 10^2^ ^b^	**75**/25 (***endo*‐*p*‐NO_2_‐benzyl**)/(endo‐alkyl)	**60**/40 (***endo‐p*‐NO_2_‐benzyl**)/(endo‐alkyl)
**3f^+^**	(1.7 ± 0.2) × 10^4^ ^b^	(2.5 ± 0.2) × 10^3^ ^b^	**95**/5^e^ (***endo*‐*p*‐NO_2_‐benzyl**)/(endo‐benzyl)	**98/**2 (***endo*‐*p*‐NO_2_‐benzyl**)/(endo‐benzyl)

For directional axles **3d^+^–3f^+^**, the term “*endo*” refers to the portion of the axle located inside the calix[6]arene cavity.

^a^ Literature value reported for the corresponding pseudo[2]rotaxane with **1a** [30].

^b^ Determined by ^1^H NMR competition experiments.

^c^ Determined by quantitative ^1^H NMR spectroscopy using 1,1,2,2‐tetrachloroethane as internal standard.

^d^ Literature value reported for the corresponding directional pseudo[2]rotaxane with **1a** [31, 44].

^e^ Reference [9].

### 2.3 In Silico Calculations

Insights into the structure of the pseudorotaxanes **3a^+^**‒**c^+^** with both **1a** and **2** were obtained through DFT calculations at the B3LYP/6‐31G(d,p) level of theory [39]. Additionally, an analysis of the energy contributions from noncovalent interactions (NCI) [40, 41] was performed using a second‐order perturbation theory (SOPT) analysis of the Fock matrix in the NBO basis [40, 41]. The DFT‐optimized geometries of **3a** ^+^⊂**1a** and **3a** ^+^⊂**2** (SI), calculated at the B3LYP/6‐31G(d,p) level, demonstrate the formation of stabilizing hydrogen bonds between the ammonium group of the axles and the oxygen atoms at the lower rim of the calix‐wheels. In the **3a** ^+^⊂**2** pseudorotaxane, an average N···O distance of 2.9 Å and an N─H···O angle of 159.8° were calculated [42]. Similar values were observed for **3a** ^+^⊂**1a**, with an angle of 150.4° and an average N···O distance of 3.0 Å.

The secondary interaction analysis for the **3a^+^⊂2** complex indicates a significant contribution from C─H··· π interactions, accounting for 25.6% of the total stabilization energy of **3a^+^⊂2**. This value is notably higher than the 18.2% calculated for **3a^+^⊂1a**. Specifically, in the **3a^+^⊂2** pseudorotaxane, the extended π‐surface of the 1,2,3‐trimethoxybenzene moieties at the *exo*‐rim plays a crucial role, contributing 7.2% to the total stabilization energy through C─H···π contacts. This result highlights the importance of the deep aromatic cavity of **2** in providing stabilization to the **3a^+^⊂2** pseudorotaxane and supports the greater thermodynamic stability of **3a^+^⊂2** compared to **3a^+^⊂1a**, as experimentally observed.

In the case of dibenzylammonium **3b^+^**, the selectivity toward the two macrocycles **1a** and **2** is reversed, with **3b^+^** forming a more stable complex with **1a** than with **2** (Table 1), in contrast to what is observed for **3a^+^**.

DFT‐optimized structures of **3b^+^⊂1a** and **3b^+^⊂2** (see Supporting Information), calculated at the B3LYP/6‐31G(d,p) level of theory, show that in both complexes the ammonium group of the guest engages in stabilizing hydrogen‐bonding interactions with the oxygen atoms at the lower rim of the calixarene hosts (HN^+^─H···O*^calix^*). In addition, C─H···π interactions involving the α^in^‐C ***H***_2–_NH_2–_Bn fragment of **3b^+^** and the aromatic cavity are more significant in **3b^+^⊂1a** (13.1%) than in **3b^+^⊂2** (8.2%).

By contrast, the contribution of the 1,2,3‐trimethoxybenzene units of **2** is minimal, accounting for only 1.2% of the total stabilization energy of **3b^+^⊂2**. In **3b^+^⊂2**, the geometry of the guest does not allow effective interaction between the phenyl ring of **3b^+^** and the upper‐rim 1,2,3‐trimethoxybenzene unit, rendering its stabilizing contribution negligible (Figure S81; see also **3f^+^⊂2**).

Overall, although the two complexes **3b^+^⊂1a** and **3b^+^⊂2** exhibit comparable hydrogen‐bonding HN^+^─H···O*^calix^* interactions, the slightly higher stability of **3b^+^⊂1a** arises from additional favorable C─H···O interactions between the α*^out^*‐C ***H***_2–_NH_2–_Bn fragment and the oxygen atoms at the lower rim of **1a** (Figure S82). These interactions, together with enhanced C─H···π contacts of α^in^‐C ***H***_2–_NH_2–_Bn fragment within the aromatic cavity of **1a**, further reinforce the stability of the **3b^+^⊂1a** complex.

This behavior contrasts with that of **3a^+^**, for which the longer dipentylammonium chain can effectively engage in additional C─H···π interactions with the 1,2,3‐trimethoxybenzene unit, resulting in a higher stability of **3a^+^⊂2** compared to **3a^+^⊂1a**. In the case of **3b^+^**, the shorter dibenzylammonium framework is unable to productively interact with the upper‐rim 1,2,3‐trimethoxybenzene unit, and conformational adjustments in **2** within the **3b^+^⊂2** complex further weaken the C─H···O interactions at the lower rim that are instead preserved in **3b^+^⊂1a**, ultimately accounting for the observed reversal in selectivity.

With these results in hand, we focused our attention on the biphenyl axle **3c^+^**‐based pseudorotaxanes. Experimentally, a significantly higher binding constant was observed for **3c^+^** ⊂**2** compared to **3c^+^** ⊂**1a** (87 ± 2 M^−1^ for **3c^+^** ⊂**1a** and 3.8 ± 0.2 × 10^2^ M^−^^1^ for **3c^+^** ⊂**2**). This trend can be attributed to the presence of extensive π–π and C─H··· π interactions between the biphenyl moiety of **3c** ^+^ and the electron‐rich aromatic surfaces of **2** (Figure S83). Specifically, about one‐third of the total π–π stabilization in **3c** ^+^⊂**2** arises from these interactions with the substituent (8.8% of the total stabilization energy), while the remaining two‐thirds originate from stacking within the calixarene cavity (23.9%). The total π–π interaction energy calculated for the **3c** ^+^⊂**2** complex is significantly higher than that calculated for **3c** ^+^⊂**1a**. Favorable C─H···π interactions are observed between the (*α*)CH_2–_N^+^ groups of the axle **3c^+^** and the oxygen lone pairs of the calixarene. In both complexes, **3c^+^** ⊂**1a** and **3c^+^** ⊂**2**, the ^+^NH_2_ group forms stabilizing hydrogen bonding interactions with the phenolic oxygen atoms of the host, with comparable interaction energies. The average N···O distances and N─H···O angles are 2.9 Å/166.2° for **3c^+^** ⊂**2** and 2.9 Å/155.3° for **3c^+^⊂1a**, respectively [42]. These results highlight that the greater stability of the pseudorotaxane **3c** ^+^⊂**2** is likely attributable to the extended π‐surface of **2**, which promotes cooperative π–π and C─H···π NCI that collectively reinforce the thermodynamic stability of the complex.

Despite the deep aromatic cavity of **2** and its ability to enhance π–π and CH–π interactions with **3c^+^**, we observe from Table 1 that **2** binds the dibenzylammonium axleaxis **3b^+^** (670 M^−^^1^) more strongly than the biphenyl axleaxis **3c^+^** (380 M^−^^1^). This difference can be attributed to the strength of the N^+^─H···O*^calix^* hydrogen bond (Figures S83 and S84). Analysis of the DFT structures indicates that this bond is stronger in the complex **3b^+^⊂2**, exhibiting a mean N^+^─H···O*^calix^* angle of 166° (Figure S84), which is more linear compared to the mean angle of 147° measured for **3c^+^⊂2** (Figure S83).

### 2.4 Threading Studies With Directional Constitutionally Non‐Symmetric Linear Axle **3d^+^−f^+^**

As previously reported by our group [5, 6, 9, 10, 15, 17, 30, 43] the threading of directional alkylbenzylammonium cations with calix[6]arene macrocycles—characterized by an *exo*–rim distinct from the *endo*–rim—can yield two distinct stereoisomeric pseudorotaxanes (Figure 4), in which either the alkyl or the benzyl group is located within the calixarene cavity. These isomers are designated as *endo*‐alkyl and *endo*‐benzyl, respectively (Figure 4) [5, 6, 9, 10, 15, 17, 30, 43]. Generally, the *endo*‐alkyl orientation is preferred, leading to the establishment of the *endo*‐alkyl rule [30, 43]. This rule indicates that the threading of a directional alkylbenzylammonium axle through a hexaalkoxycalix[6]arene occurs preferentially in the *endo*‐alkyl orientation [43]. The ^1^H NMR spectra (Figure 5) of the 1:1 mixtures of **2** with the directional ammonium axles **3d** ^+^–**3f** ^+^ in CDCl_3_ at 298 K show the typical signatures of the formation of pseudorotaxane complexes. Now, the question arises as to whether the presence of trimethoxybenzene units at the *exo*‐rim of **2** may influence the stereochemistry of threading in relation to the *endo*‐alkyl rule. In all cases examined, upon the addition of axles **3d** ^+^–**3f** ^+^ as barfate salts to a solution of **2** in CDCl_3_, the ^1^H NMR signals of the ArCH_2_Ar methylene groups in **2** appear as AX systems, indicating the conformational locking of the calix[6]arene wheel in a cone‐like arrangement [34, 35, 36] after the formation of the pseudorotaxane complexes.

**FIGURE 4:**
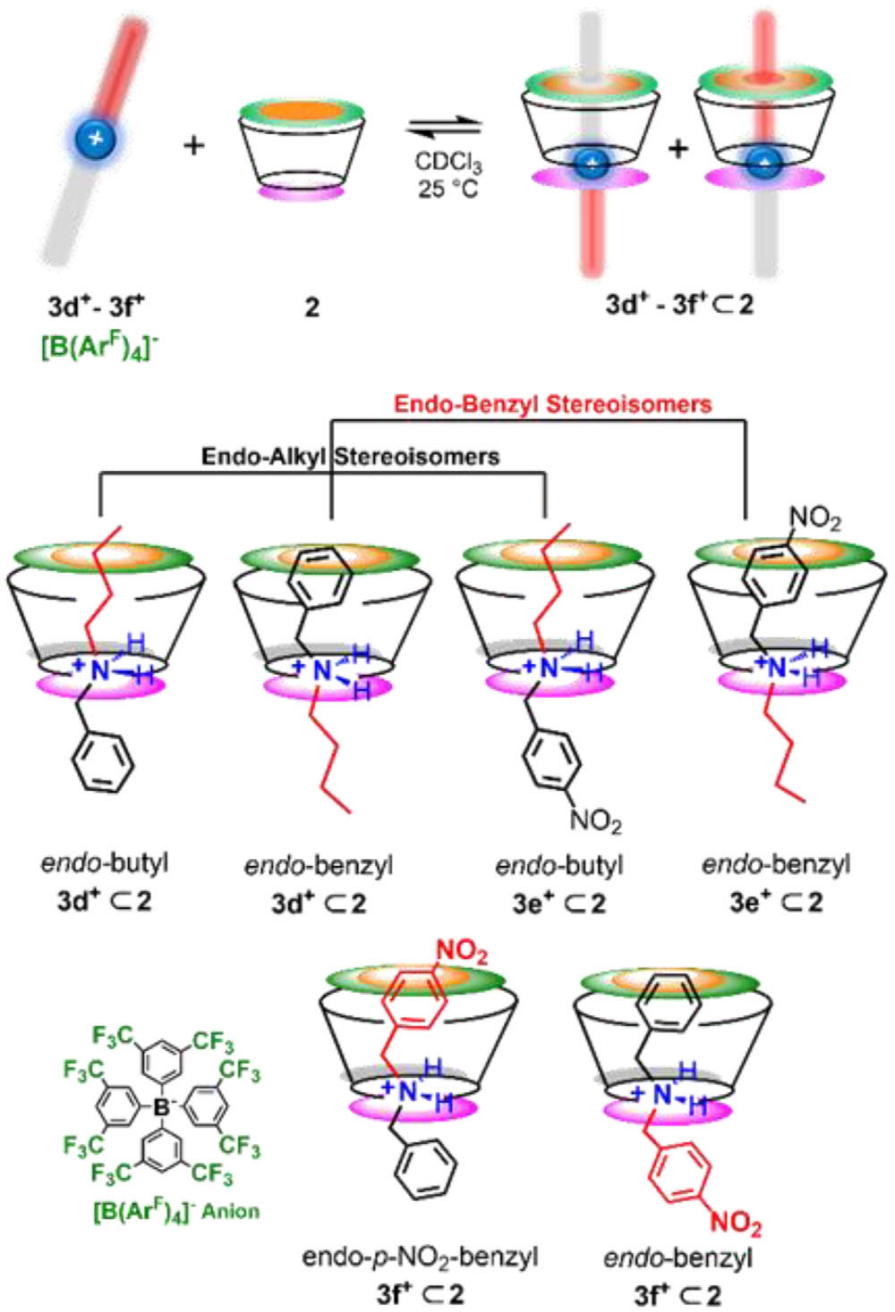
The threading of the directional ammonium axles **3d** ^+^–**3f** ^+^ through calix[6]arene **2** in CDCl_3_ results in the formation of the corresponding orientational pseudo[2]rotaxane stereoisomers, which include *endo*‐alkyl, *endo*‐benzyl, and *endo*‐*p*‐NO_2_‐benzyl configurations.

**FIGURE 5:**
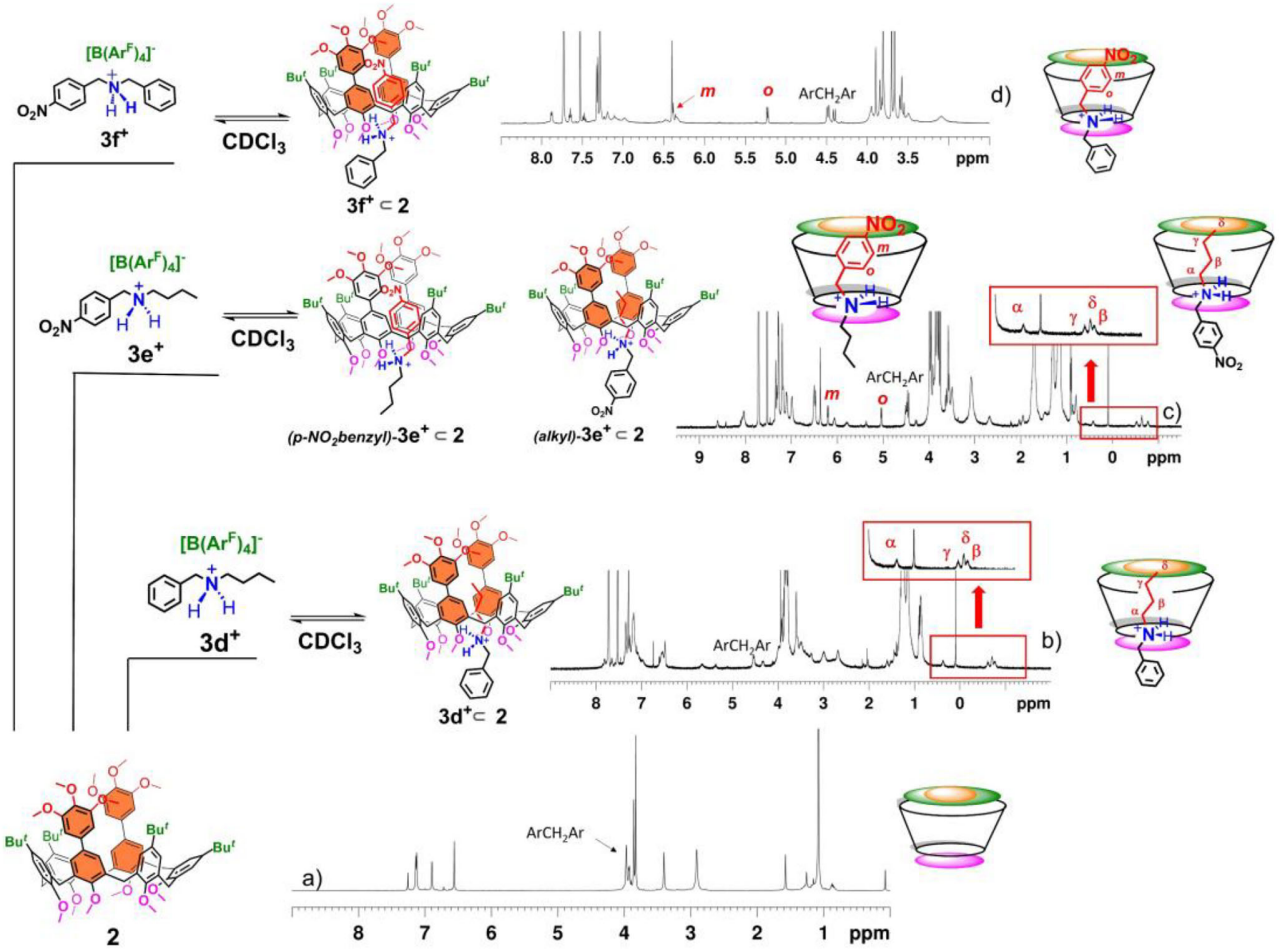
^1^H NMR spectra (600 MHz, CDCl_3_, 298 K) of: (a) free calix[6]arene **2,** and 1:1 mixture of **2** with the directional ammonium axles **3d^+^** (in b), **3e^+^** (in c), and **3f^+^** (in d). The insert in (b) highlights the upfield‐shifted α–d resonances of the threaded butyl chain in **3d^+^** ⊂**2**.

For **3d** ^+^⊂**2**, the markedly upfield‐shifted *α–δ* resonances of the butyl chain (Figures 4 and 5b) indicate its inclusion within the aromatic cavity of **2**, supporting the predominant formation of the *endo*‐butyl stereoisomer. In fact, as it is known [30, 44], the threading of butylbenzylammonium **3d** ^+^ as a barfate salt with calix[6]arene **1a** resulted in a 90:10 mixture of the pseudorotaxane stereoisomers: (*endo‐*butyl)‐**3d** ^+^⊂**1a**/(*endo‐*benzyl)‐**3d** ^+^⊂**1a** (Table 1) [30, 44]. This stereoselectivity is further enhanced during threading of **3d** ^+^ with **2**, which yielded a ratio of 95:5 for (*endo‐*butyl)‐**3d** ^+^⊂**2**/(*endo‐*benzyl)‐**3d** ^+^⊂**2**. This confirms the high affinity of the extended aromatic cavity of **2** for alkyl chains, likely due to the strong C─H···π interactions.

At this point, given the presence of two electron‐rich units at the *exo*‐rim of calixarene **2**, we aimed to investigate its threading capabilities with the benzyl‐*p*‐NO_2_‐benzylammonium axle (**3f^+^**), which possesses an electron‐poor *p‐*NO_2_‐benzyl unit. Previously [9], we demonstrated that threading calixarene **1a** with **3f^+^** resulted in the formation of pseudorotaxanes, exhibiting an *endo*‐*p‐*NO_2_‐benzyl/*endo*‐benzyl stereoisomeric ratio of 95:5 [9]. This outcome was attributed to favorable π–π interactions between the electron‐poor *p‐*NO_2_‐benzyl ring and the electron‐rich aromatic cavity of **1a**, which guided the stereochemistry of the threading process.

Analogously, the threading of **3f^+^** with **2** results in a similar stereoisomeric (*endo‐p‐*NO_2_‐benzyl)/(*endo*‐benzyl) ratio of 98:2 (Table 1). Consequently, the presence of the 1,2,3‐trimethoxybenzene moieties at the *exo* rim of calix[6]arene **2** does not significantly alter the stereochemistry of the threading interactions with axle **3f^+^**. This result is consistent with that observed for the **3b^+^⊂2** complex. Indeed, in both the *endo*‐*p*‐NO_2_‐benzyl and *endo*‐benzyl orientations, the aromatic moiety of **3f^+^** located inside the cavity does not establish sufficiently strong interactions with the 1,2,3‐trimethoxybenzene unit at the upper rim of **2**. As a consequence, the contribution of this aromatic unit to the overall stabilization of the complex is negligible.

Encouraged by this finding, we investigated the directional threading of calixarenes **1a** and **2** with the benzyl‐*p‐*NO_2_‐butylammonium axle **3e^+^**. In both cases, pseudorotaxanes **3e** ^+^⊂**1** and **3e** ^+^⊂**2** were formed (Figure 5c), and it was noteworthy that, for the first time, the *endo*‐alkyl rule was disregarded, favoring the *endo‐p*‐NO_2_‐benzyl stereoisomer (Table 1 and Figure 5c), also driven by π–π interactions. The stereoselectivity ratios, (*endo‐p*‐ NO_2_‐benzyl)/(*endo*‐butyl), were 75:25 for **1a** and 60:40 for **2** (Table 1). Notably, the percentage of the *endo*‐butyl stereoisomer was higher in **2** (40%) than in **1a** (25%), confirming the enhanced capability of the extended aromatic cavity of **2** to establish strong C─H.··· π interactions with the alkyl chains.

### 2.5 In Silico Calculations

To investigate the impact of *exo*‐rim π ‐functionalization on the threading of directional ammonium axles, density functional theory (DFT) calculations were conducted. A comparison was made between calix[6]arene **1a** and the π‐functionalized scaffold **2**. For each axle, the orientational *endo*‐alkyl/*endo*‐benzyl stereoisomers (Figure 5) were analyzed by DFT and NBO/NCI calculations.

For the butylbenzylammonium axle **3d** ^+^, calculations indicate a clear preference for the *endo*‐butyl orientation in both derivatives **1a** and **2**. The (*endo‐butyl*)‐**3d** ^+^⊂**2** stereoisomer is more stable than the corresponding *endo*‐benzyl one by 8.64 kcal mol^−^^1^, consistent with the experimentally observed formation of the *endo*‐alkyl pseudo[2]rotaxane in solution. In both (*endo*‐*butyl*)‐**3d** ^+^⊂**1a** and (*endo*‐*butyl*)‐**3d** ^+^⊂**2**, stabilization primarily arises from N^+^─H···O hydrogen bonds between the ammonium group of **3d** ^+^ and the oxygen atoms at the lower rim of calixarenes **1a** and **2**, with average N···O distances of 2.9 Å for both complexes. The N─H···O angle is 165.6° for (*endo*‐butyl)‐**3d** ^+^⊂**1a** and 156.4° for (*endo*‐butyl)‐**3d** ^+^⊂**2** [42]. Notably, C─H··· π interactions between the butyl chain of **3d** ^+^ and the aromatic cavity of the calixarene account for 19% of the total energy stabilization of the complex in (*endo*‐butyl)‐**3d** ^+^⊂**1a** and 30% in (*endo*‐butyl)‐**3d** ^+^⊂**2**, with 8% directly associated with contacts involving the 1,2,3‐trimethoxybenzene units in **2**. These results, once again in accordance with the *endo‐*alkyl rule, confirm that the C─H··· π interactions between the alkyl chain and the aromatic calixarene cavity play a crucial role in the thermodynamic stabilization of the calixarene pseudorotaxanes. These findings highlight that by increasing the π‐surface area of the calixarene cavity in **2**, the C─H··· π interactions become more significant and pivotal.

Interestingly, the “*endo*‐alkyl rule” observed for **3d^+^** likely originates from a network of cooperative and synergistic stabilizing interactions involving both the *endo*‐cavity inclusion of the alkyl chain of **3d^+^** inside the calix‐wheels **1a/2** and secondary interactions between the benzyl moiety of **3d^+^** and the methoxy groups located at the lower rim of **1a/2**. As revealed by DFT and NCI analyses (Figures S50, S66, and S66b), the *endo*‐cavity orientation of the alkyl chain of **3d^+^** not only maximizes favorable C─H···π interactions with the aromatic calixarene‐cavity but also positions the benzyl group of **3d^+^** to engage in stabilizing C─H···O and C─H···π contacts with the methoxy substituents at the calixarene‐lower‐rim. This additional “external” stabilization likely contributes to shifting the thermodynamic equilibrium toward the *endo*‐alkyl orientation, acting in concert with the stabilization provided by the multiple C─H···π interactions between the alkyl chain of **3d^+^** and the aromatic calix‐cavity.

This dual and synergistic stabilization may also account for the higher affinity of both calix‐wheels **1a** and **2** toward the butylbenzylammonium axle **3d^+^** compared with the symmetric dibenzylammonium **3b** ^+^ and dipentylammonium **3a** ^+^ axles. Indeed, symmetric axle **3a,b** ^+^ cannot simultaneously optimize both sets of interactions because of their inherent structural redundancy.

Considering these results, we need to examine the behavior of axle **3e^+^**, which features a butyl chain and an electron‐deficient aromatic *p*‐nitrobenzyl unit. Interestingly, upon the threading of **3e^+^** with the π‐functionalized host **2**, a 40% *endo‐*butyl stereoisomer was observed (Figure 5c and Table 1), which is higher than the 25% obtained when **3e^+^** was threaded with calixarene **1a**. This indicates that the presence of the extended electron‐rich cavity in **2** enhances the affinity for the *endo‐*butyl stereoisomer compared to **1a**. This observation aligns with DFT analysis: (*endo‐*butyl)‐**3e^+^** ⊂**1a** is favored by 0.8 kcal mol^−1^ with respect to (*endo‐p‐*NO_2_‐benzyl)‐**3e^+^** ⊂**1a**. This energy difference between the two stereoisomers increases to 4.63 kcal mol^−1^ in the case of the calix‐wheel **2**. NBO/NCI analysis indicates that (*endo*‐butyl)‐**3e^+^⊂2** and (*endo‐p*‐NO_2_‐benzyl)‐**3e^+^⊂2** are comparably stabilized by N^+^─H···O hydrogen bonds. Additional stabilization arises from CH··· π interactions between the included butyl chain of **3e^+^** and the aromatic walls of **2**, contributing to the total stabilization energy of the (*endo*‐butyl)‐**3e^+^⊂2** complex to an extent double that observed for C‐H··· π in (*endo*‐butyl)‐**3e^+^⊂1a**. In other words, the more extended aromatic cavity of **2** enables a greater number of CH···π interactions, thereby enhancing the stabilization of the *endo‐*butyl stereoisomer compared to macrocycle **1a**.

## 3 Conclusion

In summary, a new 1,4‐π‐functionalized calix[6]arene wheel **2** was synthesized and explored as a platform for ammonium axle threading. NMR experiments confirmed the formation of pseudo[2]rotaxanes with both symmetric **3a** ^+^−**3c** ^+^ and directional **3d** ^+^−**3f** ^+^ axles. A comparison with the parent calix[6]arene **1a** revealed that *exo*‐rim π‐functionalization influences the threading process in a manner strongly dependent on the axle. The most pronounced stabilization was observed with the π‐extended biphenyl axle **3c** ^+^, where host **2** enables large π–π contacts with the guest.

In directional systems, the π‐functionalized *exo*‐rim modulates, rather than overrides, the intrinsic orientational preferences dictated by the calix[6]arene's lower rim. As a result, **2** preserves the *endo*‐alkyl preference for **3d^+^**, reduces the *endo‐p‐*nitrobenzyl selectivity for **3e^+^,** and maintains the strong *endo‐p*‐nitrobenzyl preference for **3f^+^**. DFT calculations show these effects stem from a subtle redistribution of N^+^─H···O hydrogen bonding, C─H··· π interactions, and π—π stacking.

Therefore, *exo*‐rim π‐functionalization emerges as a powerful tool to tune both the stability and orientational outcome of calix[6]arene‐based pseudo[2]rotaxanes, offering a rational design principle for advanced interpenetrated and mechanically interlocked architectures.

## 4 Experimental Section

### 4.1 General Comments

All chemical reagents were used as obtained by TCI, Fluorochem, and Merck, with no additional purification. Reaction temperatures were measured externally and were monitored by Merck TLC silica gel plates (0.25 mm) and visualized by UV light 254 nm, or by spraying with H_2_SO_4_‐Ce(SO_4_)_2_. NMR spectra were recorded on a Bruker Avance‐600 [600 (^1^H) and 150 MHz (^13^C)] and Avance‐300 [300 (^1^H) and 75 MHz (^13^C)] spectrometers. Chemical shifts are reported relative to the residual solvent peak [45]. Standard pulse programs, provided by the manufacturer, were used for 2D COSY (cosygpqf), 2D HSQC (hsqcedetgpsisp2.2), and 2D NOESY (noesygpphpp) experiments. Structural assignments were made with additional information from gCOSY, gHSQC, and geNOESY experiments. HR MALDI mass spectra were recorded on a Bruker Solaris XR Fourier transform ion cyclotron resonance mass spectrometer equipped with a 7T refrigerated actively shielded superconducting magnet.

### 4.2 Synthesis of Derivative **7**

Derivative **6** (2.00 g, 1.95 mmol) was dissolved in dry DCM (60 mL) and, drop by drop, a solution of bromine in dry DCM was added (0.34 g, 2.15 mmol in 20 mL). The reaction was carried out in the dark for 3 h, after which the reaction mixture was evaporated, and the crude product was purified by precipitation with methanol. Derivative **7** was obtained with a yield of 90% (1,87 g, 1.75 mmol). **^1^H NMR** (300 MHz, CDCl_3_, 298 K): δ 8.47 (s, ─OH, 2H), 7.01 (s, ArH─OCH_3_, 4H), 6.98 (s, ArH─OCH_3_, 8H), 6.86 (s, ArH─OH, 4H), 3.96 (s, ─ArCH_2_Ar─, 4H), δ 3.81 (s, ─ArCH_2_Ar─, 8H), 3.23 (s, ─OCH_3_, 12H), 1.18 (s, ─C(CH_3_)_3_, 36H). **^13^C NMR** (150 MHz, CDCl_3_, 298 K): δ 153.1, 151.3, 147.3, 134.0, 131.4, 130.2, 130.1, 127.1, 126.4, 111.8, 61.5, 34.4, 31.5, and 31.2. **MALDI‐MS** (*m/z*) calcd for [M]**^+^** = 1074.3832 found 1074.3850. calcd for [M + Na]**^+^** = 1097.3724 found 1097.3758. calcd for [M + K]**^+^** = 1113.3463 found 1113.3495.

### 4.3 Synthesis of Derivative **8**

Derivative **7** (0.50 g, 0.46 mmol) was dissolved in acetone (120 mL). Subsequently, cesium carbonate (4.50 g, 13.81 mmol) was added to the reaction. After 30 min, methyl iodide (6.60 g, 46.50 mmol) was added, and the reaction was carried out under reflux for about 24 h. The reaction mixture evaporated, and the crude was extracted with chloroform and 1 M HCl. The organic phases were evaporated and subsequently purified by precipitation in methanol. Derivative **8** as a white solid was obtained with an overall yield of 98% (0.50 g, 0.45 mmol). **^1^H NMR** (400 MHz, CDCl_3_, 298 K): δ 7.00–6.94 (overlapped, Ar*H* 12H), 3.94–3.91 (overlapped, ─ArCH_2_Ar, 12H), 3.26 (s, ─OCH_3_, 6H), 3.03 (s, ─OCH_3_, 12H), 1.17 (s, ─C(CH_3_)_3_, 36H). **^13^C NMR** (100 MHz, CDCl_3_, 298 K): δ 155.3, 153.9, 146.3, 137.3, 133.9, 132.9, 131.3, 126.6, 126.0, 116.4, 60.4, 60.1, 34.3, 31.6, and 30.6. **MALDI‐MS** (*m/z*) calcd for [M]**^+^** = 1102.4145 found 1102.4114, calcd for [M + K]**^+^** = 1141.3776 found 1141.3798.

### 4.4 Synthesis of Derivative **2**

Derivative **8** (0.20 g, 0.18 mmol) was dissolved in THF (50 mL) and water (15 mL). After that, boronic acid (0.61 g, 1.45 mmol), XPhos Pd G3 palladium catalyst (0.076 g, 0.09 mmol), and potassium carbonate (0.25 g, 1.81 mmol) were added to the reaction mixture. The reaction was carried out at 80°C for 3 h under magnetic stirring. Subsequently, the reaction mixture was cooled to room temperature. CH_2_Cl_2_ was added, and the mixture was washed with water and a saturated solution of NaCl. The organic phases were collected, dried, and filtered. Purification was carried out by chromatography with eluents CHCl_3_/CH_3_OH 90:10. Derivative **2,** as a white solid, was obtained with a yield of 21% (0.05 g, 0.04 mmol). **^1^H NMR** (600 MHz, CDCl_3_, 298 K): δ 7.14–7.12 (overlapped, ArH, 8H), 6.90 (s, ArH, 4H), 6.56 (s, ArH─(OCH_3_)_3_, 4H) 3.97–3.92 (overlapped, Ar─CH_2_─Ar, 12H), 3.86‐3.83 (overlapped, OCH_3_, 18H), 3.40 (s, Ar─OCH_3_, 6H) 2.91 (s, Ar─OCH_3_ **_,_** 12H), 1.08 (s, ─C(CH_3_)_3_, 36H). **^13^C NMR** (150 MHz, CDCl_3_, 298 K): δ 155.7, 154.1, 153.5, 146.0, 137.3, 137.1, 136.2, 135.2, 133.9, 133.5, 126.9, 126.4, 126.2, 104.0, 61.0, 60.5, 60.0, 56.1, 34.2, 31.5, 31.4, 30.8, and 30.7. **MALDI‐MS** (*m/z*) calcd for [M]**^+^** = 1276.7209 found 1276.7217.

### 4.5 Synthesis of Derivative **3e^+^** [B(Ar^F^)_4_]^−^

To *p*‐nitrobenzaldehyde (10.0 mmol) was added butylamine (10.0 mmol). The reaction mixture was stirred at room temperature for 2 h to give the imine intermediate in a quantitative yield. The imine was used for the next step without further purification. The imine (10.0 mmol) was dissolved in dry MeOH (20 mL) under a nitrogen atmosphere, and NaBH_4_ (10.0 mmol) was added at 0°C. Then the mixture was allowed to warm at room temperature. The solution was kept under stirring for 3 h. The solvent was removed under reduced pressure, and the residue was partitioned between AcOEt and an aqueous saturated solution of NaHCO_3_. The organic layer was dried over Na_2_SO_4,_ and the solvent was removed under reduced pressure, to give the secondary amine as a yellow viscous liquid. The amine was used for the next step without further purification. The crude product (10.0 mmol) was dissolved in methanol (20 mL) at room temperature, and an aqueous solution of HCl (37% w/w, 10.0 mmol) was added dropwise. The mixture was kept under stirring for 1 h. The solvent was removed under reduced pressure, and the residue was washed with acetonitrile and dried under vacuum, to give the corresponding chloride salt as a white solid. Finally, the corresponding chloride **3e** ^+^Cl^−^ (2.0 mmol) was dissolved in dry MeOH (10 mL), then sodium tetrakis[3,5‐ bis(trifluoromethyl)phenyl]borate (1 eq) was added, and the mixture was kept under stirring overnight in the dark. The solvent was removed, and deionized water was added, obtaining a brown precipitate that was filtered off and dried under vacuum to give the corresponding salt **3e** ^+^ [B(Ar^F^)_4_]^−^ (yield: 1.89 g, 1.74 mmol, 87% calculated with respect to derivative **3e^+^** Cl^−^). **^1^H NMR** (600 MHz, CD_3_OD, 298 K): δ 8.36 (d, *J* = 6.0 Hz,Ar*H*, 2H), 7.76 (d, *J* = 6.0 Hz, ArH, 2H), 7.61 (overlapped, ArH, 12H) 4.35 (s, ─Ph CH_2_N, 2H), 3.10 (t, *J* = 9.0 Hz, ─NCH_2_, 2H), 1.73 (m, ─CH_2_, 2H), 1.47 (m, ─CH_2_, 2H), 1.02 (t, *J* = 9.0 Hz, ─CH_3_, 3H), **^13^C NMR** (150 MHz, CD_3_OD, 298 K): δ 163.0, 150.0, 139.7, 135.8, 133.4, 132.1, 130.6, 130.4, 128.5, 126.7, 125.2, 124.9, 123.1, 118.5, 51.4, 29.3, 20.8, and 13.9. **Anal. Calcd** for C_43_H_29_BF_24_N_2_O_2_: C 48.16, H 2.73, N 2.61. Found: C 48.20, H 2.72, N 2.58.

### 4.6 Determination of K_ass_ Values [30, 37]

The association constant values of complexes were calculated by means of two methods: ^1^H NMR competition experiments or quantitative ^1^H NMR experiments using TCE as the internal standard [46, 47] (see Supporting Information for more details).

### 4.7 DFT Studies

The starting structures for DFT calculations were obtained by molecular mechanics calculation performed by YASARA software (ver. 25.9.17) [48, 49]. DFT studies have been performed using the method incorporated in the Gaussian 16 package [39], and using b3lyp/6‐31G(d,p) level of theory without solvent. All optimized structures were characterized by 0 imaginary frequency.

## Conflicts of Interest

The authors declare no conflicts of interest.

## Supporting information




**Supporting File**: chem71289‐sup‐0001‐SuppMat.docx

## Data Availability

The data that support the findings of this study are available in the Supporting Information of this article
