# Supplementary material for: Exploring Calixarene‐Based Pseudorotaxane Architectures: Threading Ammonium Axles With an Exo‐Rim π‐Functionalized Calix[6]Arene Macrocycle
Source: Chemistry. 2026 Jun 23;32(37):e71289. doi: 10.1002/chem.71289 (PMC13628540; doi:10.1002/chem.71289)
Supplement: Supplementary file 1 [file CHEM-32-e71289-s001.docx]

Supporting information

Exploring Calixarene-Based Pseudorotaxane Architectures: Threading Ammonium Axles with an Exo-Rim π-Functionalized Calix[6]arene Macrocycle

Veronica Iuliano, Paolo Della Sala,* Placido Neri, Donato De Simone, Rocco Del Regno, Margherita De Rosa, Carmine Gaeta* and Carmen Talotta*

Dipartimento di Chimica e Biologia, Università di Salerno, Via Giovanni Paolo II, Fisciano (SALERNO), 84084-I, ITALY

**Table of Contents**

[NMR characterization of derivatives **7**, **8**, **2** and **3e**	S2](#_Toc228728077)

[Study of Pseudo[2]Rotaxane Formation in Solution and NMR Characterization	S17](#_Toc228728078)

[^1^H NMR determination of K_a_ values	S39](#_Toc228728079)

[DFT Studies and](#_Toc228728080) [[NBO and NCI analysis	S50](#_Toc228728080)](#_Toc228728081)

[References	S115](#_Toc228728082)

# NMR characterization of derivatives 7, 8, 2 and 3e

## ^1^H NMR spectrum of derivative 7

**Figure S1.** ^1^H NMR spectrum of derivative **7** (300 MHz, CDCl_3_, 298 K).

## ^13^C NMR spectrum of derivative 7

**Figure S2.** ^13^C NMR spectrum of derivative **7** (150 MHz, CDCl_3_, 298 K).

## 2D HSQC spectrum of derivative 7

**(b)**

**(a)**

**Figure S3.** **(a)** 2D HSQC NMR spectrum of derivative **7**. (b) Portion of 2D HSQC spectrum of derivative **7** between 4.1 and 3.0 ppm (600 MHz, CDCl_3_, 298 K).

## HR-MS spectrum of derivative 7

**Figure S4.** MALDI-MS spectrum of derivative **7**.

## ^1^H NMR spectrum of derivative 8

**Figure S5.** ^1^H NMR spectrum of derivative **8** (400 MHz, CDCl_3_, 298 K).

## ^13^C NMR spectrum of derivative 8

**Figure S6.** ^13^C NMR spectrum of derivative **8** (100 MHz, CDCl_3_, 298 K).

## 2D HSQC spectrum of derivative 8

**Figure S7.** 2D HSQC spectrum of derivative **8** (150 MHz, CDCl_3_, 298 K).

## HR-MS spectrum of derivative 8

**Figure S8.** MALDI-MS spectrum of derivative **8**.

## ^1^H NMR spectrum of derivative 2

**Figure S9.** ^1^H NMR spectrum of derivative **2** (600 MHz, CDCl_3_, 298 K).

## ^13^C NMR spectrum of derivative 2

**Figure S10.** ^13^C NMR spectrum of derivative **2** (150 MHz, CDCl_3_, 298 K).

## 2D HSQC spectrum of derivative 2

**Figure S11.** 2D HSQC spectrum of derivative **2** (150 MHz, CDCl_3_, 298 K).

## HR-MS spectrum of derivative 2

**Figure S12.** MALDI-MS spectrum of derivative **2**.

## ^1^H NMR spectrum of derivative 3e^+^ [B(Ar^F^)_4_]^-^

**Figure S13.** ^1^H NMR spectrum of derivative **3e^+^ [B(Ar^F^)_4_]^-^** (600 MHz, CD_3_OD, 298 K).

## ^13^C NMR spectrum of derivative 3e^+^ [B(Ar^F^)_4_]^-^

**Figure S14.** ^13^C NMR spectrum of derivative **3e^+^ [B(Ar^F^)_4_]^-^** (150 MHz, CD_3_OD, 298 K).

## 2D COSY spectrum of derivative 3e^+^ [B(Ar^F^)_4_]^-^

**Figure S15.** 2D COSY spectrum of derivative **3e^+^ [B(Ar^F^)_4_]^-^** (600 MHz, CD_3_OD, 298 K).

# Study of Pseudo[2]Rotaxane Formation in Solution and NMR Characterization

Figure S16. Hosts and guests studied in this work.

A 1:1 mixture of the calix[6]arene derivative (**1a** or **2**) and **3a-f**^+^ ammonium guests was dissolved in 0.5 ml of CDCl_3_. Table S1 details the concentrations used for the calix[6]arene derivatives and the axles. Then, the solution was transferred to an NMR tube for the acquisition of 1D and 2D NMR spectra.

**Table S1.** Concentrations used for threading studies among calixarenes **1a** and **2** and axles **3a^+^**-**3f^+^**

| **Pseudo[2]**  **rotaxanes** | **Calix[6]arenes** | | **Axles** | | **CDCl_3_** |
| --- | --- | --- | --- | --- | --- |
|  | mg | mmol | mg | mmol | **ml** |
| **3a^+^ ⊂ 2** | 2.00 | 1.56 x 10^-3^ | 1.60 | 1.56 x 10^-3^ | 0.5 |
| **3b^+^ ⊂ 2** | 2.00 | 1.56 x 10^-3^ | 1.66 | 1.56 x 10^-3^ | 0.5 |
| **3c^+^ ⊂ 2** | 2.00 | 1.56 x 10^-3^ | 1.88 | 1.56 x 10^-3^ | 0.5 |
| **3d^+^ ⊂ 2** | 2.00 | 1.56 x 10^-3^ | 1.61 | 1.56 x 10^-3^ | 0.5 |
| **3e^+^ ⊂ 2** | 2.00 | 1.56 x 10^-3^ | 1.68 | 1.56 x 10^-3^ | 0.5 |
| **3f^+^ ⊂ 2** | 2.00 | 1.56 x 10^-3^ | 1.73 | 1.56 x 10^-3^ | 0.5 |
| **3c^+^ ⊂ 1a** | 2.00 | 1.89 x 10^-3^ | 2.27 | 1.89 x 10^-3^ | 0.5 |
| **3e^+^ ⊂ 1a** | 2.00 | 1.89 x 10^-3^ | 2.09 | 1.89 x 10^-3^ | 0.5 |
| **3f^+^ ⊂ 1a** | 2.00 | 1.89 x 10^-3^ | 2.03 | 1.89 x 10^-3^ | 0.5 |

## ^1^H NMR spectrum of pseudo[2]rotaxane 3a^+^ ⊂ 2

**Figure S17.** ^1^H NMR spectrum of **3a^+^** ⊂ **2** (600 MHz, CDCl_3_, 298 K).

## 2D COSY spectrum of pseudo[2]rotaxane 3a^+^ ⊂ 2

**Figure S18.** 2D COSY spectrum of **3a^+^** ⊂ **2** (600 MHz, CDCl_3_, 298 K).

## ^1^H NMR spectrum of pseudo[2]rotaxane 3b^+^ ⊂ 2

**Figure S19.** ^1^H NMR spectrum of **3b^+^** ⊂ **2** (600 MHz, CDCl_3_, 298 K).

## 2D COSY spectrum of pseudo[2]rotaxane 3b^+^ ⊂ 2

**(b)**

**(c)**

**(a)**

**Figure S20.** **(a)** 2D COSY spectrum of **3b^+^** ⊂ **2** (600 MHz, CDCl_3_, 298 K). **(b)** Portion of 2D COSY spectrum of **3b^+^⊂ 2** between 4.8 and 3.1 ppm and **(c)** between 6.7 and 4.7 ppm**.**

## 2D HSQC spectrum of pseudo[2]rotaxane 3b^+^ ⊂ 2

**Figure S21.** 2D HSQC spectrum of **3b^+^ ⊂ 2** (600 MHz, CDCl_3_, 298 K).

## ^1^H NMR spectrum of pseudo[2]rotaxane 3c^+^ ⊂ 2

**Figure S22.** ^1^H NMR spectrum of **3c^+^ ⊂ 2** (600 MHz, CDCl_3_, 298 K).

## 2D COSY spectrum of pseudo[2]rotaxane 3c^+^ ⊂ 2

**Figure S23.** 2D COSY spectrum of **3c^+^** ⊂ **2** (600 MHz, CDCl_3_, 298 K).

## ^1^H NMR spectrum of pseudo[2]rotaxane 3d^+^ ⊂ 2

**Figure S24.** ^1^H NMR spectrum of **3d^+^⊂ 2** (600 MHz, CDCl_3_, 298 K).

## 2D COSY spectrum of pseudo[2]rotaxane 3d^+^ ⊂ 2

**(c)**

**(b)**

**(a)**

**Figure S25.** **(a)** 2D COSY spectrum of **3d^+^ ⊂ 2** (600 MHz, CDCl_3_, 298 K). **(b)** Portion of 2D COSY spectrum of **3d^+^⊂ 2** between 5.0 and 3.0 ppm and **(c)** between 0.6 and -1.2 ppm**.**

## ^1^H NMR spectrum of pseudo[2]rotaxane 3e^+^ ⊂ 2

**Figure S26.** ^1^H NMR spectrum of **3e^+^ ⊂ 2** (600 MHz, CDCl_3_, 298 K).

## 2D COSY spectrum of pseudo[2]rotaxane 3e^+^ ⊂ 2

**(a)**

**(c)**

**(b)**

**Figure S27.** **(a)** 2D COSY spectrum of **3e^+^ ⊂ 2** (600 MHz, CDCl_3_, 298 K). **(b)** Portion of 2D COSY spectrum of **3e^+^⊂ 2** between 6.8 and 4.7 ppm and **(c)** between 1.0 and -1.3 ppm**.**

## 2D HSQC spectrum of pseudo[2]rotaxane 3e^+^ ⊂ 2

**(c)**

**(b)**

**(a)**

**Figure S28.** **(a)** 2D HSQC spectrum of **3e^+^ ⊂ 2** (600 MHz, CDCl_3_, 298 K). **(b)** Portion of 2D HSQC spectrum of **3e^+^⊂ 2** between 9.0 and 4.5 ppm and **(c)** between -0.4 and -0.9 ppm**.**

## ^1^H NMR spectrum of pseudo[2]rotaxane 3f^+^ ⊂ 2

**Figure S29.** ^1^H NMR spectrum of **3f^+^ ⊂ 2** (600 MHz, CDCl_3_, 298 K).

## 2D COSY spectrum of pseudo[2]rotaxane 3f^+^ ⊂ 2

**(a)**

**Figure S30.** **(a)** 2D COSY spectrum of **3f^+^** **⊂ 2** (600 MHz, CDCl_3_, 298 K). **(b)** Portion of 2D COSY spectrum of **3f^+^** ⊂ **2** between 7.0 and 5.5 ppm and **(c)** between 4.8 and 3.4 ppm

## 2D HSQC spectrum of pseudo[2]rotaxane 3f^+^ ⊂ 2

**(a)**

**Figure S31.** **(a)** 2D HSQC spectrum of **3f^+^ ⊂ 2** (600 MHz, CDCl_3_, 298 K). **(b)** Portion of 2D HSQC spectrum of **3f^+^ ⊂ 2** between 5.0 and 3.0 ppm and **(c)** between 8.5 and 5.0 ppm.

## ^1^H NMR spectrum of pseudo[2]rotaxane 3c^+^ ⊂ 1a

**Figure S32.** ^1^H NMR spectrum of **3c^+^⊂ 1a** (600 MHz, CDCl_3_, 298 K).

## 2D COSY spectrum of pseudo[2]rotaxane 3c^+^ ⊂ 1a

**Figure S33.** 2D COSY spectrum of **3c^+^ ⊂ 1a** (600 MHz, CDCl_3_, 298 K).

## ^1^H NMR spectrum of pseudo[2]rotaxane 3e^+^ ⊂ 1a

**Figure S34.** ^1^H NMR spectrum of **3e^+^ ⊂ 1a** (600 MHz, CDCl_3_, 298 K).

## 2D COSY spectrum of pseudo[2]rotaxane 3e^+^ ⊂ 1a

**(a)**

**(b)**

**Figure S35.** **(a)** 2D COSY spectrum of **3e^+^** ⊂ **1a** (600 MHz, CDCl_3_, 298 K); **(b)** Portion of 2D COSY spectrum of **3e^+^** ⊂ **1a** between 6.5 and 4.5 ppm.

## ^1^H NMR spectrum of pseudo[2]rotaxane 3f^+^ ⊂ 1a

Figure S36. ^1^H NMR spectrum of 3f^+^ ⊂ 1a (600 MHz, CDCl_3_, 298 K).

## 2D COSY spectrum of pseudo[2]rotaxane 3f^+^ ⊂ 1a

**(a)**

**Figure S37.** **(a)** 2D COSY spectrum of **3f^+^ ⊂ 1a** (600 MHz, CDCl_3_, 298 K).; **(b)** Portion of 2D COSY spectrum of **3f^+^ ⊂ 1a** between 6.5 and 4.5 ppm and **(c)** between 8.2 and 7.0 ppm.

# ^1^H NMR determination of K_a_ values

The association constant values of complexes were calculated by means of two methods:

a) ^1^H NMR competition experiments. An analysis of a 1:1:1 mixture of host, and two guests (or two hosts and one guest) was performed in an NMR tube using 0.5 mL of CDCl_3_ as solvent. The following equation was used to obtain K_a_ value.

$K_{A\subset H}=\frac{\left[ \mathrm{HG}\text{A} \right]}{\left[ H \right]\left[ G\text{A} \right]}\quad\text{and}\quad K_{B\subset H}=\frac{\left[ \mathrm{HG}\text{B} \right]}{\left[ H \right]\left[ G\text{B} \right]}\to K_{\mathrm{rel}}=\frac{K_{A\subset H}}{K_{B\subset H}}=\frac{\left[ \mathrm{HG}\text{A} \right]\left[ H \right]\left[ G\text{B} \right]}{\left[ \mathrm{HG}\text{B} \right]\left[ H \right]\left[ G\text{A} \right]}$

$$\left[ \mathrm{HG}\text{A} \right]=\left[ G\text{B} \right]$$

$$\left[ \mathrm{HG}\text{B} \right]=\left[ G\text{A} \right]$$

$$K_{\mathrm{rel}}=\frac{K_{A\subset H}}{K_{B\subset H}}=\frac{\left[ \mathrm{HG}\text{A} \right]^{2}}{\left[ \mathrm{HG}\text{B} \right]^{2}}$$

b) Quantitative ^1^H NMR experiments using TCE as the internal standard. In this case, ^1^H NMR experiments were carried out on a 1:1 mixture of host and guest in 0.5 mL of CDCl_3_ containing 1 μL of 1,1,2,2-tetrachloroethane (d= 1.586 g/mL) as internal standard. The following equation was used to obtain the moles of the complex:

$$\frac{G_{a}}{G_{b}}=\frac{F_{a}}{F_{b}}\times\frac{N_{b}}{N_{a}}\times\frac{M_{a}}{M_{b}}$$

Where:

*G*_a_ = grams of 1,1,2,2-Tetrachloroethane**;** *G*_b_= grams of pseudorotaxane.

*F*_a_ and *F*_b_ = areas of the signal related of 1,1,2,2-tetrachloroethane and pseudorotaxane.

*N*_a_ and *N*_b_ = numbers of nuclei which cause the signals (*N*_a_ for 1,1,2,2-tetrachloroethane**;** *N*_b_ for pseudorotaxane)

*M*_a_ and *M*_b_ = molecular masses of 1,1,2,2-tetrachloroethane (a) and pseudorotaxane (b).

Table S2. K_a_ values measured for the formation of the pseudorotaxanes.

|  | **1a** | **2** |
| --- | --- | --- |
| **3a^+^** | 4.4±0.2 x 10^2^ M^–1 (1)^ | 5.2±0.2 x 10^2^ M^–1^ |
| **3b^+^** | 2.5±0.2 x 10^3^ M^–1 (1)^ | 6.7±0.2 x 10^2^ M^–1^ |
| **3c^+^** | 87±2 M^–1^ | 3.8±0.2 x 10^2^ M^–1^ |
| **3d^+^** | 6.5±0.2 x 10^4^ M^–1 (1)^ | 2.1±0.2 x 10^3^ M^–1^ |
| **3e^+^** | 8.3±0.2 x 10^3^ M^–1^ | 9.3±0.2 x 10^2^ M^–1^ |
| **3f^+^** | 1.7±0.2 x 10^4^ M^–1^ | 2.5±0.2 x 10^3^ M^–1^ |

**Table S3.** Relative stability constants (K_rel_) calculated by ^1^H NMR competition experiments.

|  | $\boldsymbol{K}_{{\boldsymbol{3}\boldsymbol{b}}^{\boldsymbol{+}}\boldsymbol{\subset}\mathbf{1a}}$  **(2.5±0.2x10^3^ M^–1^)^(1)^** | $\boldsymbol{K}_{{\boldsymbol{3}\boldsymbol{f}}^{\boldsymbol{+}}\boldsymbol{\subset}\mathbf{2}}$  **(2.5±0.2 x 10^3^ M^–1^)** | $\boldsymbol{K}_{{\boldsymbol{3}\boldsymbol{e}}^{\boldsymbol{+}}\boldsymbol{\subset}\mathbf{2}}$  (**9.3±0.2 x 10^2^** **M^–1^)** | $\boldsymbol{K}_{{\boldsymbol{3}\boldsymbol{c}}^{\boldsymbol{+}}\boldsymbol{\subset}\mathbf{2}}$  **(3.8±0.2 x 10^2^ M^–1^)** |
| --- | --- | --- | --- | --- |
| $\boldsymbol{K}_{{\boldsymbol{3}\boldsymbol{f}}^{\boldsymbol{+}}\boldsymbol{\subset}\mathbf{1a}}$  **(1.7±0.2 x 10^4^ M^–1^)** | $\boldsymbol{K}_{\boldsymbol{rel}}\boldsymbol{=}\frac{\boldsymbol{K}_{{\boldsymbol{3}\boldsymbol{f}}^{\boldsymbol{+}}\boldsymbol{@1}\boldsymbol{a}}}{\boldsymbol{K}_{{\boldsymbol{3}\boldsymbol{b}}^{\boldsymbol{+}}\boldsymbol{@1}\boldsymbol{a}}}\boldsymbol{=6.69}$ | $\boldsymbol{K}_{\boldsymbol{rel}}\boldsymbol{=}\frac{\boldsymbol{K}_{{\boldsymbol{3}\boldsymbol{f}}^{\boldsymbol{+}}\boldsymbol{@2}}}{\boldsymbol{K}_{{\boldsymbol{3}\boldsymbol{f}}^{\boldsymbol{+}}\boldsymbol{@1}\boldsymbol{a}}}\boldsymbol{=0.15}$ |  |  |
| $\boldsymbol{K}_{{\boldsymbol{3}\boldsymbol{e}}^{\boldsymbol{+}}\boldsymbol{\subset}\mathbf{1a}}$  **(8.3±0.2 x 10^3^ M^–1^)** | $\boldsymbol{K}_{\boldsymbol{rel}}\boldsymbol{=}\frac{\boldsymbol{K}_{{\boldsymbol{3}\boldsymbol{e}}^{\boldsymbol{+}}\boldsymbol{@1}\boldsymbol{a}}}{\boldsymbol{K}_{{\boldsymbol{3}\boldsymbol{b}}^{\boldsymbol{+}}\boldsymbol{@1}\boldsymbol{a}}}\boldsymbol{=3.31}$ |  | $\boldsymbol{K}_{\boldsymbol{rel}}\boldsymbol{=}\frac{\boldsymbol{K}_{{\boldsymbol{3}\boldsymbol{e}}^{\boldsymbol{+}}\boldsymbol{@4}}}{\boldsymbol{K}_{{\boldsymbol{3}\boldsymbol{e}}^{\boldsymbol{+}}\boldsymbol{@1}\boldsymbol{a}}}\boldsymbol{=0.62}$ |  |
| $\boldsymbol{K}_{{\boldsymbol{3}\boldsymbol{b}}^{\boldsymbol{+}}\boldsymbol{\subset}\mathbf{2}}$  **(6.7±0.2 x 10^2^ M^–1^)** | $\boldsymbol{K}_{\boldsymbol{rel}}\boldsymbol{=}\frac{\boldsymbol{K}_{{\boldsymbol{3}\boldsymbol{b}}^{\boldsymbol{+}}\boldsymbol{@2}}}{\boldsymbol{K}_{{\boldsymbol{3}\boldsymbol{b}}^{\boldsymbol{+}}\boldsymbol{@1}\boldsymbol{a}}}\boldsymbol{=0.27}$ |  |  | $\boldsymbol{K}_{\boldsymbol{rel}}\boldsymbol{=}\frac{\boldsymbol{K}_{{\boldsymbol{3}\boldsymbol{b}}^{\boldsymbol{+}}\boldsymbol{@2}}}{\boldsymbol{K}_{{\boldsymbol{3}\boldsymbol{c}}^{\boldsymbol{+}}\boldsymbol{@2}}}\boldsymbol{=1.77}$ |
| $\boldsymbol{K}_{{\boldsymbol{3}\boldsymbol{c}}^{\boldsymbol{+}}\boldsymbol{\subset}\mathbf{1a}}$  **(1.0±0.2 x 10^2^ M^–1^)** |  |  |  | $\boldsymbol{K}_{\boldsymbol{rel}}\boldsymbol{=}\frac{\boldsymbol{K}_{{\boldsymbol{3}\boldsymbol{c}}^{\boldsymbol{+}}\boldsymbol{@1}\boldsymbol{a}}}{\boldsymbol{K}_{{\boldsymbol{3}\boldsymbol{c}}^{\boldsymbol{+}}\boldsymbol{@2}}}\boldsymbol{=0.23}$ |

## K_a_ value of 3a^+^⊂ 2

**Figure S38.** ^1^H NMR spectrum of an equimolar solution (6.1 mM) of **3a^+^** and **2** in 0.5 mL of CDCl_3_ containing 1 μL of TCE. The association constant *K*_a_ value was calculated by integration of signal of complex **3a^+^⊂ 2** (▲) and TCE (■) (600 MHz, CDCl_3_, 298 K).

## K_a_ value of 3e^+^⊂ 1a

**(a)**

**(b)**

**(c)**

**Figure S39.** ^1^H NMR spectrum of (a) an equimolar solution (4.5 mM) of **3b^+^** and **1a** in 0.5 mL of CDCl_3_; (b) an equimolar solution (4.5 mM) of **3b^+^**, **1a** and **3e^+^** in 0.5 mL of CDCl_3_. (c) an equimolar solution (4.5 mM) of **3e^+^** and **1a** in 0.5 mL of CDCl_3_.The association constant *K*_a_ value was calculated by integration of signal of complex **3b^+^**⊂ **1a** (▲) and complex **3e^+^**⊂ **1a** (■) (600 MHz, CDCl_3_, 298 K).

## K_a_ value of 3f^+^⊂ 1a

**(a)**

**(b)**

**(c)**

**Figure S40.** ^1^H NMR spectrum of (a) an equimolar solution (4.5 mM) of **3b^+^** and **1a** in 0.5 mL of CDCl_3_; (b) an equimolar solution (4.5 mM) of **3b^+^**, **1a** and **3f^+^** in 0.5 mL of CDCl_3_. (c) an equimolar solution (4.5 mM) of **3f^+^** and **1a** in 0.5 mL of CDCl_3_.The association constant Ka value was calculated by integration of signal of complex **3b^+^⊂ 1a** (▲) and complex **3f^+^⊂ 1a** (■) (600 MHz, CDCl_3_, 298 K).

## K_a_ value of 3b^+^⊂ 2

**(a)**

**(b)**

**(c)**

**Figure S41.** ^1^H NMR spectrum of (a) an equimolar solution (4.5 mM) of **3b^+^** and **1a** in 0.5 mL of CDCl_3_; (b) an equimolar solution (4.5 mM) of **3b^+^**, **1a** and **2** in 0.5 mL of CDCl_3_. (c) an equimolar solution (4.5 mM) of **3b^+^** and **2** in 0.5 mL of CDCl_3_.The association constant *K*_a_ value was calculated by integration of signal of complex **3b^+^**⊂ **1a** (▲) and complex **3b^+^**⊂ **2** (■) (600 MHz, CDCl_3_, 298 K).

## K_a_ value of 3c^+^⊂ 2

**(a)**

**(b)**

**(c)**

**Figure S42.** ^1^H NMR spectrum of (a) an equimolar solution (4.5 mM) of **3b^+^** and **2** in 0.5 mL of CDCl_3_; (b) an equimolar solution (4.5 mM) of **3b^+^**, **3c^+^** and **2** in 0.5 mL of CDCl_3_. (c) an equimolar solution (4.5 mM) of **3c^+^** and **2** in 0.5 mL of CDCl_3_.The association constant Ka value was calculated by integration of signal of complex **3b^+^ ⊂ 2** (▲) and complex **3c^+^ ⊂ 2** (■) (600 MHz, CDCl3, 298 K).

## K_a_ value of 3c^+^⊂ 1a

**(a)**

**(b)**

**(c)**

**Figure S43.** ^1^H NMR spectrum of (a) an equimolar solution (4.5 mM) of **3c^+^** and **2** in 0.5 mL of CDCl_3_; (b) an equimolar solution (4.5 mM) of **3c^+^**, **2** and **1a** in 0.5 mL of CDCl_3_. (c) an equimolar solution (4.5 mM) of **3c^+^** and **1a** in 0.5 mL of CDCl_3_.The association constant *K*_a_ value was calculated by integration of signal of complex **3c^+^**⊂ **1a** (▲) and complex **3c^+^** ⊂ **2** (■) (600 MHz, CDCl_3_, 298 K).

## K_a_ value of 3d^+^⊂ 2

**Figure S44.** ^1^H NMR spectrum of an equimolar solution (6.1 mM) of **3d^+^** and **2** in 0.5 mL of CDCl_3_ containing 1 μL of TCE. The association constant *K*_a_ value was calculated by integration of the signal of complex **3d^+^**⊂ **2** (▲) and TCE (■) (600 MHz, CDCl_3_, 298 K).

## K_a_ value of 3e^+^⊂ 2

**(a)**

**(b)**

**(c)**

**Figure S45.** ^1^H NMR spectrum of (a) an equimolar solution (4.5 mM) of **3e^+^** and **1a** in 0.5 mL of CDCl_3_; (b) an equimolar solution (4.5 mM) of **3e^+^**, **1a** and **2** in 0.5 mL of CDCl_3_. (c) an equimolar solution (4.5 mM) of **3e^+^** and **2** in 0.5 mL of CDCl_3_.The association constant Ka value was calculated by integration of the signal of complex **3e^+^⊂ 1a** (▲) and complex **3e^+^⊂ 2** (■) (600 MHz, CDCl_3_, 298 K).

## K_a_ value of 3f^+^⊂ 2

**(a)**

**(b)**

**(c)**

**Figure S46.** ^1^H NMR spectrum of (a) an equimolar solution (4.5 mM) of **3f^+^** and **1a** in 0.5 mL of CDCl_3_; (b) an equimolar solution (4.5 mM) of **3f^+^**, **1a** and **2** in 0.5 mL of CDCl_3_. (c) an equimolar solution (4.5 mM) of **3f^+^** and **2** in 0.5 mL of CDCl_3_.The association constant *K*_a_ value was calculated by integration of signal of complex **3f^+^**⊂ **1a** (▲) and complex **3f^+^**⊂ **2** (■) (600 MHz, CDCl_3_, 298 K).

# DFT Studies

The starting structures for DFT calculations were obtained by molecular mechanics calculation performed by YASARA software (ver. 25.9.17).^2^ DFT studies have been performed using the method incorporated in the Gaussian 16 package,^3^ and using b3lyp/6-31G(d,p) level of theory in the gas phase, including Grimme’s D3 dispersion correction. All optimized structures were characterized by 0 imaginary frequency.

**DFT structure of 3a-f**⊂**1a complexes**

**3a^+^**⊂**1a**

**Figure S47.** DFT-optimized structure of the **3a^+^**⊂**1a** complex.

O	2.23034300	-3.54836200	2.32913300

O	-1.66334800	-2.97902900	2.30569000

O	3.90379600	0.52862400	1.87464700

O	2.96462700	3.66124900	2.29291200

O	-1.41103700	2.60214400	2.59627200

O	-3.47992400	-0.15487800	2.85072000

C	-2.17101400	-3.61742000	1.16627200

C	-1.53305600	5.14827300	-0.07205000

H	-0.85468200	5.85202100	-0.53991900

C	-3.50871900	-3.41300100	0.79834600

C	1.28827100	-3.82394700	-1.21818100

H	0.48064900	-4.23737600	-1.81272900

C	-1.01080600	4.30499900	0.92017400

C	-3.98112000	-4.05399100	-0.35639000

H	-5.02138600	-3.91300000	-0.62266600

C	-1.89477800	3.43222900	1.57205100

C	-1.32349300	-4.45619200	0.41765500

C	2.78488200	3.60112800	0.91459800

C	5.25192200	-1.04931600	0.63470100

C	-1.85453900	-5.07925600	-0.71448200

H	-1.21287500	-5.76009400	-1.26600800

C	-4.84103200	-1.20659100	1.14796200

C	3.35423800	-2.76401500	0.34222100

C	1.36199200	3.85866800	-1.01019900

H	0.39449900	4.11129600	-1.42870200

C	-4.46570400	-2.59328300	1.66025900

H	-5.39019400	-3.17253100	1.76681400

H	-4.03861600	-2.49005100	2.65446700

C	1.22258700	-3.96307600	0.17142800

C	2.27825800	-3.43944900	0.94032800

C	2.36374800	-3.19347700	-1.86063300

C	-4.36087300	-0.03990600	1.77048600

C	3.37202200	-2.65522700	-1.05260600

H	4.20528400	-2.13559900	-1.50630700

C	1.52550800	3.93270400	0.37360000

C	6.31136700	-1.27068000	-0.26009400

H	6.57582100	-2.29942800	-0.47951700

C	-3.26237200	3.38459500	1.24499500

C	2.41033700	3.49206600	-1.87389100

C	0.44281600	4.42127200	1.33560200

H	0.60132100	3.90233800	2.28042100

C	-3.17868200	-4.88907800	-1.13764600

C	4.92077300	0.26981100	0.96075700

C	3.83560800	3.17882900	0.09425800

C	-4.72638800	1.23427300	1.31215700

C	-3.72056800	4.23945300	0.23909900

H	-4.78031400	4.23036000	0.00587600

C	0.10340000	-4.74835400	0.85249800

H	0.20156000	-4.58809700	1.92439500

C	5.59181200	1.35339700	0.36587800

C	-4.24231100	2.50099700	2.01149500

H	-3.79082000	2.21755900	2.95898800

H	-5.12270200	3.11139200	2.24444300

C	3.62840900	3.13917000	-1.29086100

H	4.45805300	2.82256800	-1.91250900

C	-3.69814300	-5.61609100	-2.39225200

C	6.62697600	1.08183300	-0.52934100

H	7.14638500	1.92549600	-0.97541000

C	2.89513600	-4.69924100	2.86195500

H	2.79683000	-4.64265000	3.94805700

H	2.43392000	-5.62717000	2.50349000

C	-5.60789600	1.31872700	0.22611100

H	-5.90799700	2.30415700	-0.10720500

C	-2.88122600	5.13339500	-0.44143500

C	-6.12895600	0.18929300	-0.41148300

C	7.02337700	-0.22588300	-0.85505900

C	-5.72083100	-1.06202800	0.07282200

H	-6.12053300	-1.96112800	-0.38459200

C	-1.46601900	3.20542800	3.89938400

H	-2.48551400	3.52025800	4.14397600

H	-0.80203600	4.07366200	3.95819200

C	2.43187900	-3.16074700	-3.40055300

C	-7.14582000	0.27434000	-1.56592400

C	-3.46438800	6.06580200	-1.51999800

C	1.16552100	-2.49207600	-3.98105900

H	0.25148400	-3.01580800	-3.68515300

H	1.20492300	-2.49203700	-5.07568100

H	1.08250200	-1.45311000	-3.64709000

C	3.44702200	4.92008300	2.77130200

H	4.42763100	5.16174000	2.34405400

H	2.75323200	5.73639100	2.53746000

C	2.52504100	-4.61382600	-3.92651400

H	3.41661300	-5.11572800	-3.53730500

H	2.58191300	-4.62064300	-5.02043900

H	1.65347000	-5.20749200	-3.63446400

C	-4.09494200	5.21340800	-2.64692000

H	-4.51742900	5.86174600	-3.42176600

H	-4.90172400	4.57533300	-2.27376000

H	-3.34628400	4.56740900	-3.11734100

C	-1.80809700	-3.74063400	3.51382700

H	-2.86333400	-3.90340800	3.75895000

H	-1.31417500	-4.71391800	3.42989200

C	-5.16675600	-5.27108300	-2.70287600

H	-5.83896400	-5.57403600	-1.89348800

H	-5.48513100	-5.80008800	-3.60605500

H	-5.30464200	-4.19977700	-2.88442800

C	-2.84009600	-5.20935000	-3.61389100

H	-2.89349500	-4.12984500	-3.79016700

H	-3.19846900	-5.71746100	-4.51535100

H	-1.78759500	-5.47766400	-3.48164800

C	3.65838200	-2.38418000	-3.91660200

H	3.65731600	-2.38152200	-5.01091900

H	4.59831800	-2.84136400	-3.59146400

H	3.65144900	-1.34133600	-3.58248000

C	2.20246700	3.53469700	-3.40053200

C	-3.59561700	-7.14548800	-2.18020300

H	-2.56502000	-7.46583300	-2.00123600

H	-3.95787800	-7.67625400	-3.06697200

H	-4.19900500	-7.46463800	-1.32439600

C	-4.55220100	6.96743500	-0.88879300

H	-4.13364300	7.58659500	-0.08893900

H	-5.37432900	6.38336500	-0.46474000

H	-4.97705900	7.63450800	-1.64622700

C	-2.39295800	6.97622900	-2.14888500

H	-1.93002300	7.63643100	-1.40841000

H	-2.85328500	7.61209300	-2.91092600

H	-1.60128200	6.39939600	-2.63867200

C	-8.47703600	-0.37477600	-1.11621500

H	-8.89168800	0.14213500	-0.24488100

H	-8.34744100	-1.42802300	-0.84991500

H	-9.21560800	-0.32474700	-1.92333300

C	-7.43638000	1.72821300	-1.98377200

H	-7.87788900	2.30901800	-1.16742300

H	-8.15087800	1.73641000	-2.81227100

H	-6.53163600	2.24275700	-2.32489500

C	3.45655100	3.08767000	-4.17560000

H	3.25927900	3.13130400	-5.25118500

H	3.74112800	2.05834900	-3.93355700

H	4.31436300	3.73672600	-3.97344300

C	1.03559100	2.60660100	-3.80590900

H	0.09981900	2.88478300	-3.31172100

H	1.25909400	1.56596200	-3.54871600

H	0.86671900	2.65712600	-4.88726100

C	-6.60196100	-0.47978500	-2.80194700

H	-7.32715900	-0.43490900	-3.62128500

H	-6.41266700	-1.53599100	-2.58811100

H	-5.66562500	-0.03501100	-3.15359900

C	1.86836500	4.98570900	-3.82345100

H	0.95368400	5.34791700	-3.34416400

H	1.72242600	5.04388800	-4.90761800

H	2.67965400	5.66901000	-3.55318800

H	0.63525900	5.48354100	1.54328000

C	8.19401300	-0.45488200	-1.82908600

C	7.85757600	0.18431500	-3.19739800

C	9.47376600	0.20125200	-1.25818500

C	8.48265300	-1.95070000	-2.05588400

H	9.32132900	-2.06226300	-2.74978600

H	8.75618900	-2.45967900	-1.12591000

H	7.62427900	-2.47168100	-2.49310700

H	8.68341700	0.03365400	-3.90096100

H	6.95773100	-0.26550700	-3.63003600

H	7.68656000	1.26166100	-3.11412300

H	10.31551200	0.05115500	-1.94273900

H	9.35147200	1.27879700	-1.11483300

H	9.74117000	-0.23667100	-0.29112000

H	0.28697700	-5.81653800	0.67734200

H	-1.33440000	-3.16509000	4.31137000

H	3.95795100	-4.70677400	2.59681600

H	3.53987000	4.82818600	3.85565800

H	-1.13921700	2.44987200	4.61585300

C	-4.12681300	-0.21667700	4.12869900

H	-4.71817600	0.68587900	4.31929300

H	-4.78673600	-1.08842600	4.20243700

H	-3.33732700	-0.29619700	4.87954000

C	4.37194100	0.71170100	3.21395800

H	3.55256000	1.16241000	3.77645400

H	4.65908700	-0.24527900	3.66922100

H	5.23484400	1.38452600	3.25168000

C	2.07988000	-1.51270700	7.64929200

H	2.17268100	-2.58712500	7.45598700

H	1.82634600	-1.38662000	8.70587300

C	1.02195100	-0.87519700	6.74432200

H	3.06475500	-1.06095200	7.48801800

H	0.92770200	0.19206100	6.98606300

C	1.33738400	-1.03014100	5.25122900

H	0.04087000	-1.32220400	6.95602300

C	0.27542100	-0.39469600	4.34253500

H	2.31539700	-0.58056500	5.03797900

H	1.43380300	-2.09489100	5.00077900

C	0.59532200	-0.58465400	2.86146900

H	-0.70337800	-0.84250000	4.56356000

H	0.18990700	0.67491900	4.57124800

N	-0.56260400	-0.17641200	1.98994500

H	1.46311200	0.00075700	2.54603000

H	0.79429400	-1.63413700	2.63840700

C	-0.24332800	-0.22116400	0.51547400

C	-1.48528500	0.03177000	-0.33116900

H	0.53232100	0.52948900	0.34098000

H	0.18103900	-1.20886600	0.31949500

C	-1.19637600	-0.09336800	-1.83178900

H	-2.26338200	-0.68942600	-0.05731400

H	-1.88521500	1.02927300	-0.11456100

C	-2.44165700	0.16473400	-2.69018300

H	-0.40427100	0.61135300	-2.11394200

H	-0.80933500	-1.09826600	-2.04200600

C	-2.18386000	0.01054200	-4.19173100

H	-3.23716600	-0.52508900	-2.38035200

H	-2.81903000	1.17483000	-2.48184000

H	-3.09241700	0.19955100	-4.77229400

H	-1.41731600	0.71154100	-4.53938400

H	-1.84023600	-1.00115400	-4.43427900

H	-1.35009500	-0.81419300	2.18384800

H	-0.89013100	0.77529700	2.24021700

C	5.18820200	2.78572300	0.67084100

H	5.95708100	3.45059800	0.26094000

H	5.16984100	2.95217800	1.74875500

C	4.48615800	-2.22016200	1.21431100

H	5.19832000	-3.03482500	1.40372700

H	4.06184800	-1.95323600	2.18320600

1 30 1.0 59 1.0

2 7 1.0 90 1.0

3 43 1.0 165 1.0

4 18 1.0 79 1.0

5 16 1.0 69 1.0

6 32 1.0 161 1.0

7 10 1.5 17 1.5

8 9 1.0 13 1.5 64 1.5

9

10 14 1.5 26 1.0

11 12 1.0 29 1.5 31 1.5

12

13 16 1.5 40 1.0

14 15 1.0 42 1.5

15

16 38 1.5

17 20 1.5 48 1.0

18 35 1.5 44 1.5

19 36 1.5 43 1.5 207 1.0

20 21 1.0 42 1.5

21

22 26 1.0 32 1.5 67 1.5

23 30 1.5 33 1.5 207 1.0

24 25 1.0 35 1.5 39 1.5

25

26 27 1.0 28 1.0

27

28

29 30 1.5 48 1.0

30

31 33 1.5 72 1.0

32 45 1.5

33 34 1.0

34

35 40 1.0

36 37 1.0 66 1.5

37

38 46 1.5 51 1.0

39 54 1.5 105 1.0

40 41 1.0 142 1.0

41

42 56 1.0

43 50 1.5

44 54 1.5 204 1.0

45 51 1.0 62 1.5

46 47 1.0 64 1.5

47

48 49 1.0 156 1.0

49

50 57 1.5 204 1.0

51 52 1.0 53 1.0

52

53

54 55 1.0

55

56 93 1.0 97 1.0 106 1.0

57 58 1.0 66 1.5

58

59 60 1.0 61 1.0 158 1.0

60

61

62 63 1.0 65 1.5

63

64 74 1.0

65 67 1.5 73 1.0

66 143 1.0

67 68 1.0

68

69 70 1.0 71 1.0 160 1.0

70

71

72 75 1.0 82 1.0 101 1.0

73 118 1.0 122 1.0 134 1.0

74 86 1.0 110 1.0 114 1.0

75 76 1.0 77 1.0 78 1.0

76

77

78

79 80 1.0 81 1.0 159 1.0

80

81

82 83 1.0 84 1.0 85 1.0

83

84

85

86 87 1.0 88 1.0 89 1.0

87

88

89

90 91 1.0 92 1.0 157 1.0

91

92

93 94 1.0 95 1.0 96 1.0

94

95

96

97 98 1.0 99 1.0 100 1.0

98

99

100

101 102 1.0 103 1.0 104 1.0

102

103

104

105 126 1.0 130 1.0 138 1.0

106 107 1.0 108 1.0 109 1.0

107

108

109

110 111 1.0 112 1.0 113 1.0

111

112

113

114 115 1.0 116 1.0 117 1.0

115

116

117

118 119 1.0 120 1.0 121 1.0

119

120

121

122 123 1.0 124 1.0 125 1.0

123

124

125

126 127 1.0 128 1.0 129 1.0

127

128

129

130 131 1.0 132 1.0 133 1.0

131

132

133

134 135 1.0 136 1.0 137 1.0

135

136

137

138 139 1.0 140 1.0 141 1.0

139

140

141

142

143 144 1.0 145 1.0 146 1.0

144 150 1.0 151 1.0 152 1.0

145 153 1.0 154 1.0 155 1.0

146 147 1.0 148 1.0 149 1.0

147

148

149

150

151

152

153

154

155

156

157

158

159

160

161 162 1.0 163 1.0 164 1.0

162

163

164

165 166 1.0 167 1.0 168 1.0

166

167

168

169 170 1.0 171 1.0 172 1.0 173 1.0

170

171

172 174 1.0 175 1.0 176 1.0

173

174

175 177 1.0 178 1.0 179 1.0

176

177 180 1.0 181 1.0 182 1.0

178

179

180 183 1.0 184 1.0 185 1.0

181

182

183 186 1.0 202 1.0 203 1.0

184

185

186 187 1.0 188 1.0 189 1.0

187 190 1.0 191 1.0 192 1.0

188

189

190 193 1.0 194 1.0 195 1.0

191

192

193 196 1.0 197 1.0 198 1.0

194

195

196 199 1.0 200 1.0 201 1.0

197

198

199

200

201

202

203

204 205 1.0 206 1.0

205

206

207 208 1.0 209 1.0

208

209

___________________________

**0 imaginary frequency**

**Energy: -3703.1192 Hartree**

**3b^+^**⊂**1a**

**Figure S48.** DFT-optimized structure of the **3b^+^**⊂**1a** complex.

O	2.10354300	-4.25711300	2.20165200

O	-1.81854600	-3.24451700	2.40789200

O	3.90857900	-0.42692100	2.12990900

O	2.90749100	3.39136700	2.35844800

O	-0.88652500	2.93951600	2.27744800

O	-3.08435800	-0.00604400	2.59311500

C	-2.39402300	-3.72098000	1.22834300

C	-1.13915000	5.30341500	-0.53504700

H	-0.49925200	6.01861200	-1.03956400

C	-3.68707800	-3.31365800	0.86757300

C	0.85023500	-3.92905200	-1.24081400

H	-0.04707600	-4.16368800	-1.80261800

C	-0.58531600	4.56581500	0.52022400

C	-4.24086400	-3.83333300	-0.31228100

H	-5.24643300	-3.52963200	-0.57475400

C	-1.42103500	3.67660100	1.21272000

C	-1.65720800	-4.60850700	0.41986700

C	2.94847000	3.34766800	0.96651900

C	5.17805500	-1.80803300	0.61064300

C	-2.26589800	-5.10828900	-0.73363200

H	-1.70940200	-5.82561700	-1.32997600

C	-4.78929200	-0.98510300	1.18589400

C	3.14541600	-3.32609900	0.23458100

C	2.00965900	3.94577900	-1.16765000

H	1.24217500	4.46355500	-1.73412000

C	-4.50373400	-2.37409500	1.75064400

H	-5.46786800	-2.85514800	1.95346700

H	-3.98898200	-2.27092000	2.70281100

C	0.88986600	-4.27291900	0.11320000

C	2.05452700	-3.96649900	0.84314800

C	1.92531200	-3.30587500	-1.89264800

C	-4.09061000	0.15105700	1.63021000

C	3.05622200	-3.00664900	-1.12565600

H	3.90329300	-2.51191300	-1.58316200

C	1.93869800	3.99274000	0.22712000

C	6.20210300	-1.90588900	-0.34233700

H	6.44780800	-2.89423300	-0.71500500

C	-2.77875100	3.52287800	0.87719500

C	3.03706800	3.27567800	-1.84946700

C	0.85796700	4.80016200	0.94170600

H	0.96266900	4.61025900	2.00815300

C	-3.56116300	-4.74009200	-1.12952900

C	4.86737700	-0.54344500	1.12450400

C	3.98121500	2.64499100	0.32637900

C	-4.38139600	1.43592000	1.14689200

C	-3.27281400	4.28229000	-0.18700600

H	-4.32566300	4.19649400	-0.43202000

C	-0.24900800	-5.03563800	0.79011500

H	-0.10246100	-4.95245900	1.86597500

C	5.51046000	0.61203800	0.65304700

C	-3.70724300	2.67020500	1.74095800

H	-3.14447000	2.35108300	2.61309900

H	-4.50601100	3.33093100	2.10199100

C	3.99710500	2.61787700	-1.07367200

H	4.79484700	2.06824500	-1.55673600

C	-4.17904800	-5.34825300	-2.40260700

C	6.52798100	0.46100000	-0.29331300

H	7.04605300	1.35538700	-0.62855600

C	2.65973000	-5.53595200	2.52230200

H	2.64239100	-5.61938400	3.61093500

H	2.06841200	-6.35071000	2.08734600

C	-5.40416000	1.55991800	0.19685100

H	-5.65577900	2.55313200	-0.15173600

C	-2.47979000	5.17954500	-0.91637800

C	-6.13370200	0.46442900	-0.27340000

C	6.90924300	-0.79002600	-0.80293700

C	-5.80529100	-0.79621000	0.24641400

H	-6.37946600	-1.66252300	-0.06656700

C	-1.00879700	3.59835800	3.55064700

H	-0.56244800	2.93837000	4.29327500

H	-2.05989700	3.77950300	3.79859700

C	1.84389700	-3.01244300	-3.40360500

C	-7.28777700	0.60170400	-1.28512600

C	-3.11050600	6.02513600	-2.04023200

C	0.62453500	-2.11170100	-3.70579500

H	-0.31628300	-2.57377700	-3.39427900

H	0.55676600	-1.91690700	-4.78169900

H	0.70580600	-1.14864400	-3.19192000

C	3.64911700	4.47181400	2.93512100

H	4.71322500	4.40845900	2.68058100

H	3.26297600	5.44251000	2.60219500

C	1.69094800	-4.34946300	-4.16853000

H	2.54300500	-5.01032000	-3.97943300

H	1.63541100	-4.16724900	-5.24729500

H	0.78221600	-4.88344900	-3.87463400

C	-3.74637700	5.09845200	-3.10275100

H	-4.22099700	5.69479100	-3.88910200

H	-4.51285100	4.44632700	-2.67383400

H	-2.99039600	4.46185400	-3.57416300

C	-2.07689100	-4.08829100	3.53794000

H	-3.14507900	-4.10769000	3.78459300

H	-1.74090300	-5.11495100	3.35587700

C	-5.59784900	-4.81544600	-2.67733900

H	-6.29074400	-5.05840100	-1.86517300

H	-5.99157500	-5.27235400	-3.59014700

H	-5.60567300	-3.73004400	-2.82479500

C	-3.29152200	-5.00571700	-3.62274500

H	-3.21070100	-3.92209400	-3.75954500

H	-3.72099500	-5.43071100	-4.53613000

H	-2.27925800	-5.40744400	-3.51783700

C	3.10540900	-2.30195000	-3.92984000

H	2.99868600	-2.11041400	-5.00197800

H	4.00530600	-2.91024400	-3.79337000

H	3.26589300	-1.33777700	-3.43567400

C	3.10856900	3.32992200	-3.38822700

C	-4.26330900	-6.88555200	-2.24630700

H	-3.27803900	-7.33532800	-2.09275300

H	-4.69633000	-7.33523200	-3.14612200

H	-4.89305000	-7.15955400	-1.39385200

C	-4.20756500	6.93462800	-1.43576800

H	-3.78785800	7.60598200	-0.67987800

H	-5.00395500	6.35328000	-0.96167000

H	-4.66555400	7.54907000	-2.21822200

C	-2.07809000	6.92365900	-2.74716300

H	-1.63084700	7.65169600	-2.06287600

H	-2.56778200	7.48697400	-3.54712700

H	-1.27159300	6.33941200	-3.20295400

C	-8.60370700	0.11546900	-0.63193500

H	-8.84615600	0.71204300	0.25328300

H	-8.54450100	-0.93234200	-0.32307200

H	-9.43477700	0.20569000	-1.33948000

C	-7.49125300	2.05652400	-1.74859200

H	-7.75633700	2.71798600	-0.91741100

H	-8.30958100	2.10022300	-2.47348900

H	-6.59714200	2.45761100	-2.23783400

C	4.23856200	2.44850800	-3.95315300

H	4.23794500	2.50620000	-5.04589200

H	4.11238300	1.39638600	-3.67672300

H	5.22498400	2.77633900	-3.61051500

C	1.77545500	2.85151900	-4.00666700

H	0.93485500	3.48382900	-3.70596700

H	1.54524800	1.82345500	-3.71003300

H	1.83346900	2.88414000	-5.09984400

C	-6.98731700	-0.26119600	-2.53346600

H	-7.80298400	-0.17690600	-3.25934500

H	-6.87861400	-1.32050500	-2.28187600

H	-6.06402300	0.06600500	-3.02288000

C	3.37208700	4.79258300	-3.82178400

H	2.57698700	5.46396800	-3.48320400

H	3.42734100	4.86330700	-4.91357800

H	4.31740900	5.15996700	-3.40981700

H	1.07443000	5.86518200	0.78834000

C	8.06278400	-0.89003900	-1.81864600

C	7.72440600	-0.04996900	-3.07289400

C	9.35987600	-0.34635000	-1.17317100

C	8.32140000	-2.34017300	-2.26886100

H	9.14688800	-2.36027900	-2.98676200

H	8.60115600	-2.98514300	-1.42963000

H	7.44700700	-2.77753400	-2.76217400

H	8.53838000	-0.11279400	-3.80300900

H	6.80981500	-0.41246300	-3.55373900

H	7.58028200	1.00718900	-2.83055100

H	10.19152000	-0.40564600	-1.88344400

H	9.25725700	0.69959800	-0.86928300

H	9.63006400	-0.92767600	-0.28577300

H	-0.14275300	-6.09929600	0.53878800

H	-1.51780800	-3.67586400	4.38027700

H	3.69413100	-5.62414600	2.17062800

H	3.53311900	4.38942100	4.01795000

H	-0.47995800	4.55684100	3.54756100

C	-3.55363200	0.08116600	3.94867900

H	-2.67715400	0.00230300	4.59273700

H	-4.05497000	1.03791300	4.12784100

H	-4.25354800	-0.72984000	4.17605400

C	4.47199600	-0.55729500	3.44338400

H	5.20901700	0.23097400	3.63668400

H	3.64938500	-0.46540700	4.15375000

H	4.95896900	-1.53043700	3.57227800

C	5.09686200	1.98774700	1.13865700

H	5.97709300	2.64299500	1.11108100

H	4.77104900	1.93730500	2.17733400

C	4.41108200	-3.04143800	1.04156300

H	5.08023700	-3.90791000	0.95731200

H	4.12597100	-2.97322100	2.09221900

C	0.65364100	-0.66735800	2.78357500

N	-0.27596000	0.01398700	1.79107100

C	0.09918500	-0.39220100	0.37016800

H	-1.26099700	-0.23170200	2.00469200

C	-0.66426100	1.29994000	-1.33742600

C	-1.51521100	1.69504600	-2.36905400

C	-2.56257400	0.86536400	-2.77087400

C	-2.75884700	-0.36215800	-2.13707100

C	-1.90996400	-0.75843700	-1.10491500

C	-0.85883700	0.07163000	-0.69349200

H	0.16151900	1.94160700	-1.04619500

H	-1.35334100	2.64763100	-2.86127000

H	-3.21724700	1.17113400	-3.58163000

H	-3.56766500	-1.01610200	-2.44573900

H	-2.05436700	-1.72382000	-0.63223600

C	1.30738900	1.53644900	5.82056800

C	1.31935400	1.05154700	4.51095400

C	0.58641000	-0.09853200	4.17823200

C	-0.14627600	-0.75108900	5.17841700

C	-0.15900700	-0.26395000	6.48637100

C	0.56870900	0.88245700	6.80919600

H	1.88439300	2.42157700	6.07130300

H	1.90320900	1.56671000	3.75113800

H	-0.69565000	-1.65463400	4.93069900

H	-0.72377800	-0.78516100	7.25329800

H	0.56951000	1.25773300	7.82790400

H	0.17003200	-1.48148100	0.38675300

H	1.10139800	0.01162200	0.21102800

H	1.66148900	-0.56569300	2.37310000

H	0.37600700	-1.72303300	2.76506300

H	-0.24507800	1.04311800	1.90015000

1 30 1.0 59 1.0

2 7 1.0 90 1.0

3 43 1.0 165 1.0

4 18 1.0 79 1.0

5 16 1.0 69 1.0

6 32 1.0 161 1.0

7 10 1.5 17 1.5

8 9 1.0 13 1.5 64 1.5

9

10 14 1.5 26 1.0

11 12 1.0 29 1.5 31 1.5

12

13 16 1.5 40 1.0

14 15 1.0 42 1.5

15

16 38 1.5

17 20 1.5 48 1.0

18 35 1.5 44 1.5

19 36 1.5 43 1.5 172 1.0

20 21 1.0 42 1.5

21

22 26 1.0 32 1.5 67 1.5

23 30 1.5 33 1.5 172 1.0

24 25 1.0 35 1.5 39 1.5

25

26 27 1.0 28 1.0

27

28

29 30 1.5 48 1.0

30

31 33 1.5 72 1.0

32 45 1.5

33 34 1.0

34

35 40 1.0

36 37 1.0 66 1.5

37

38 46 1.5 51 1.0

39 54 1.5 105 1.0

40 41 1.0 142 1.0

41

42 56 1.0

43 50 1.5

44 54 1.5 169 1.0

45 51 1.0 62 1.5

46 47 1.0 64 1.5

47

48 49 1.0 156 1.0

49

50 57 1.5 169 1.0

51 52 1.0 53 1.0

52

53

54 55 1.0

55

56 93 1.0 97 1.0 106 1.0

57 58 1.0 66 1.5

58

59 60 1.0 61 1.0 158 1.0

60

61

62 63 1.0 65 1.5

63

64 74 1.0

65 67 1.5 73 1.0

66 143 1.0

67 68 1.0

68

69 70 1.0 71 1.0 160 1.0

70

71

72 75 1.0 82 1.0 101 1.0

73 118 1.0 122 1.0 134 1.0

74 86 1.0 110 1.0 114 1.0

75 76 1.0 77 1.0 78 1.0

76

77

78

79 80 1.0 81 1.0 159 1.0

80

81

82 83 1.0 84 1.0 85 1.0

83

84

85

86 87 1.0 88 1.0 89 1.0

87

88

89

90 91 1.0 92 1.0 157 1.0

91

92

93 94 1.0 95 1.0 96 1.0

94

95

96

97 98 1.0 99 1.0 100 1.0

98

99

100

101 102 1.0 103 1.0 104 1.0

102

103

104

105 126 1.0 130 1.0 138 1.0

106 107 1.0 108 1.0 109 1.0

107

108

109

110 111 1.0 112 1.0 113 1.0

111

112

113

114 115 1.0 116 1.0 117 1.0

115

116

117

118 119 1.0 120 1.0 121 1.0

119

120

121

122 123 1.0 124 1.0 125 1.0

123

124

125

126 127 1.0 128 1.0 129 1.0

127

128

129

130 131 1.0 132 1.0 133 1.0

131

132

133

134 135 1.0 136 1.0 137 1.0

135

136

137

138 139 1.0 140 1.0 141 1.0

139

140

141

142

143 144 1.0 145 1.0 146 1.0

144 150 1.0 151 1.0 152 1.0

145 153 1.0 154 1.0 155 1.0

146 147 1.0 148 1.0 149 1.0

147

148

149

150

151

152

153

154

155

156

157

158

159

160

161 162 1.0 163 1.0 164 1.0

162

163

164

165 166 1.0 167 1.0 168 1.0

166

167

168

169 170 1.0 171 1.0

170

171

172 173 1.0 174 1.0

173

174

175 176 1.0 192 1.0 203 1.0 204 1.0

176 177 1.0 178 1.0 205 1.0

177 184 1.0 201 1.0 202 1.0

178

179 180 1.5 184 1.5 185 1.0

180 181 1.5 186 1.0

181 182 1.5 187 1.0

182 183 1.5 188 1.0

183 184 1.5 189 1.0

184

185

186

187

188

189

190 191 1.5 195 1.5 196 1.0

191 192 1.5 197 1.0

192 193 1.5

193 194 1.5 198 1.0

194 195 1.5 199 1.0

195 200 1.0

196

197

198

199

200

201

202

203

204

205

___________________________

**0 imaginary frequency**

**Energy: -3850.6958 Hartree**

**3c^+^**⊂**1a**

**Figure S49.** DFT-optimized structure of the **3c^+^**⊂**1a** complex.

O	-3.79360700	3.05974800	1.69642600

O	-4.26027400	-0.19040000	-1.05055200

O	-0.07039600	4.13005400	0.51076500

O	1.86181700	2.53421500	-2.10454800

O	0.95016100	-0.67437800	-4.07784800

O	-2.17911900	-2.19317900	-3.91688100

C	-4.82190500	-1.19020800	-0.25401300

C	4.15567500	-1.84991700	-2.68879200

C	-4.90669100	-2.50178200	-0.74937500

C	-3.16239100	-0.32008900	2.99755500

C	3.18185400	-0.97147500	-3.18312700

C	-5.58552800	-3.45128300	0.02181100

C	1.96207700	-1.52157000	-3.60555000

C	-5.31495400	-0.85634400	1.01424700

C	3.16088900	2.32463400	-1.61401900

C	0.18803100	3.65067700	2.86088600

C	-5.97114100	-1.85019200	1.75332700

C	-2.89905500	-3.51731900	-2.02297300

C	-1.94640200	2.19710100	2.99789800

C	5.29836100	1.24517400	-1.77912400

C	-4.28910400	-2.87819100	-2.08465800

C	-3.78777200	0.72822500	2.32199900

C	-3.17756700	1.99664700	2.35282900

C	-1.91587700	-3.18221300	-2.97149800

C	-1.35481500	1.10581800	3.64920200

C	3.97437700	1.34764400	-2.21707400

C	1.04511400	3.46049800	3.94706900

C	1.70811300	-2.90352800	-3.54947700

C	3.47389200	0.51676500	-3.39226000

C	-6.14385000	-3.15350900	1.27418300

C	0.76251400	3.90718200	1.60073200

C	3.63186700	3.13932000	-0.57295400

C	-0.65722700	-3.81402600	-2.97003200

C	2.73166300	-3.73159600	-3.08510500

C	-5.12078800	0.52872100	1.60039900

C	2.15523900	3.91589100	1.42485600

C	0.39055800	-3.49694600	-4.03604700

C	4.96769900	2.98569500	-0.17581400

C	-1.31913500	3.59171400	3.04369700

C	2.96813400	3.69087000	2.54556400

C	-4.71946300	3.79110000	2.50612700

C	-0.41857700	-4.80634000	-2.01483000

C	-2.59844800	-4.50709600	-1.07957000

C	0.98042000	-0.44398800	-5.49331900

C	2.76642700	4.22455600	0.05893500

C	1.80188700	3.53846800	-3.13320800

C	-5.24573500	0.44580300	-1.88057000

C	-6.91740500	-4.23471600	2.05250000

C	-0.39034300	5.51301000	0.31147600

C	-2.83348900	-2.66751300	-5.09906200

C	-1.59324200	1.02896000	-2.75418800

N	-0.38270000	0.69785200	-1.89482500

C	-0.66499900	0.41826500	-0.42523000

C	0.47711100	-0.32961400	0.21111700

C	-2.07140900	2.45500800	-2.67343300

H	5.09577600	-1.43224300	-2.35513600

H	-3.64314100	-1.29184800	2.98395500

H	-6.36735700	-1.57332200	2.72400700

H	5.95073700	0.54260000	-2.28754100

H	-4.96367100	-3.58350500	-2.58804800

H	-4.22230400	-1.99642200	-2.72056300

H	0.59844600	3.30793600	4.92553900

H	2.56708200	0.97904900	-3.77581700

H	2.56388500	-4.80424100	-3.09572800

H	-5.20192100	1.29351200	0.82626500

H	-0.06237200	-2.82485100	-4.76160000

H	0.62411100	-4.42968000	-4.56298200

H	5.34559300	3.65398600	0.58843800

H	-1.81078800	4.19811600	2.28407100

H	-1.55928400	4.04360000	4.01535100

H	4.04159500	3.70744800	2.40595500

H	-4.22096400	4.24382000	3.37137800

H	-5.13327600	4.58125800	1.87588000

H	0.54194700	-5.31133100	-2.03344900

H	-3.35682700	-4.75413600	-0.34866900

H	1.88848600	0.09453100	-5.78605200

H	0.10441500	0.16081800	-5.73743000

H	3.38546900	5.12453200	0.15990300

H	1.95133000	4.46782600	-0.61814000

H	0.74643300	3.70273400	-3.35455600

H	2.24989200	4.47508600	-2.78873900

H	-5.70460100	-0.27251600	-2.56975100

H	-6.03547200	0.90187700	-1.27323300

H	4.22593500	0.58979300	-4.18902400

H	-5.93880200	0.71856900	2.30765100

H	-0.40320700	1.26415500	4.13963400

H	-5.69680700	-4.45183800	-0.38667100

H	-4.73383800	1.22415400	-2.44723800

H	2.32761600	3.20662600	-4.03575100

H	0.93357800	-1.38623300	-6.04852800

H	-5.53456100	3.15065400	2.86275900

H	0.04383300	-0.12862800	-2.33282900

H	-1.59350200	-0.15523300	-0.39727400

H	-0.83435400	1.38543800	0.04916400

H	-2.36445700	0.31762400	-2.45166100

H	-1.29128300	0.78267700	-3.77244700

H	0.34899700	1.43309000	-1.96189200

C	-1.95166800	-0.15804700	3.68653800

C	5.82997400	2.05707200	-0.76650500

C	3.96648200	-3.23459600	-2.64100100

C	5.50697200	-5.05875400	-3.41553600

C	4.53979500	-5.15138000	-1.08727000

C	5.07126600	-4.21206500	-2.19473100

C	6.31132700	-3.48298100	-1.64361900

C	-1.21261400	-2.57831600	3.56432100

C	-1.36073500	-1.34003000	4.47888600

C	0.02362500	-1.01485700	5.06996200

C	-2.31963800	-1.68293000	5.64485600

C	7.71544700	2.91563100	0.73826400

C	7.65178400	0.51187400	0.06223800

C	8.17764100	2.29730100	-1.64006000

C	7.32266500	1.95109400	-0.39681800

C	-7.43912600	-3.71938100	3.40712800

C	-8.13188400	-4.69638500	1.21176500

C	-5.99319600	-5.44521800	2.32263400

H	4.67472300	-5.63905900	-3.82549400

H	5.89753300	-4.42261300	-4.21643300

H	3.68285000	-5.74211300	-1.42524500

H	4.23806100	-4.58644600	-0.20044200

H	6.06602600	-2.86457000	-0.77419600

H	6.78523000	-2.85040900	-2.40165800

H	-2.17245900	-2.90120200	3.15023900

H	-0.53795300	-2.37062000	2.72872500

H	0.74796500	-0.75867500	4.29032100

H	-0.02176300	-0.18671000	5.78463300

H	-3.31268000	-1.96424100	5.28116800

H	-2.44036100	-0.82831900	6.31842800

H	7.98782400	1.61135900	-2.47091900

H	7.96815400	3.31323400	-1.98974100

H	7.56303300	3.96278700	0.45776300

H	7.15122700	2.71932300	1.65617200

H	7.09040500	0.25133500	0.96534400

H	7.41663000	-0.23071000	-0.70561500

H	-5.62852000	-5.89686500	1.39535000

H	-5.12286700	-5.15036900	2.91822000

H	-6.62413800	-3.39962100	4.06493600

H	-8.13251400	-2.88086100	3.28626400

H	-8.81066900	-3.86121500	1.01144100

H	-7.82599200	-5.11705400	0.24925400

H	6.29447200	-5.76340800	-3.12748000

H	5.32200700	-5.85603800	-0.78586000

H	7.05610800	-4.21836200	-1.32464100

H	8.77693000	2.79266200	0.97325800

H	8.71875000	0.42280700	0.29263600

H	9.24358700	2.23580800	-1.39633900

H	-0.80168600	-3.41831900	4.13550800

H	0.40854100	-1.88691500	5.60781200

H	-1.92629100	-2.52234100	6.22896400

H	-7.97993600	-4.51992200	3.92080800

H	-8.69366100	-5.46866000	1.74807900

H	-6.53442600	-6.22012700	2.87592100

H	-0.90842500	5.93231100	1.18206400

H	0.51269900	6.10723300	0.12565000

H	-1.04642500	5.56091800	-0.55756700

H	-2.97718000	-1.80042200	-5.74751700

H	-2.22219500	-3.41386100	-5.62058300

H	-3.80916700	-3.11060800	-4.86980700

C	1.43993400	0.34399100	0.96981500

C	2.48754100	-0.35090800	1.56756800

C	2.60961200	-1.74205700	1.42289700

C	1.64542400	-2.40952600	0.64789700

C	0.59165400	-1.71809000	0.05675400

H	1.36227400	1.41487400	1.11476600

H	3.20272700	0.19428200	2.17468100

H	1.72710300	-3.47938200	0.49531100

H	-0.14588500	-2.26902900	-0.52079900

C	-3.25895100	4.24498600	-1.54386000

C	-2.76647600	2.94339900	-1.55777200

C	-1.89997400	3.30798400	-3.77350100

C	-2.39759900	4.60887800	-3.76069700

C	-3.08824900	5.10623100	-2.64237700

H	-3.76805800	4.59460700	-0.65252900

H	-2.94606200	2.30994900	-0.69649200

H	-1.39877100	2.94038600	-4.66610400

H	-2.28270700	5.23342500	-4.64087000

C	5.81593200	-3.87799500	3.33814000

C	6.04446700	-2.62020700	2.77725900

C	5.00514900	-1.93024000	2.15516100

C	3.71589300	-2.48374900	2.07861600

C	3.50070800	-3.75085300	2.64698400

C	4.54018500	-4.44014700	3.27028100

H	6.62454300	-4.41469400	3.82473600

H	7.03544200	-2.17768400	2.81893600

H	5.19549200	-0.96277300	1.70082500

H	2.50582000	-4.18514500	2.62541600

H	4.35043300	-5.41360900	3.71262900

C	-4.63930300	9.11734000	-2.58813600

C	-3.41486200	8.84644100	-3.20084000

C	-2.91269700	7.54649700	-3.21954100

C	-3.62365900	6.49086800	-2.62398100

C	-4.85458100	6.77879200	-2.01044600

C	-5.35746300	8.07825700	-1.99383900

H	-5.03145000	10.12953100	-2.57523500

H	-2.84621300	9.64958100	-3.65981900

H	-1.94804900	7.35192100	-3.67904100

H	-5.43469100	5.97355800	-1.56975100

H	-6.31603000	8.27826200	-1.52453200

C	-1.37474600	-5.18540500	-1.06235600

C	-1.08025600	-6.34445600	-0.08943200

C	-2.23716900	-6.58871600	0.89742200

H	-1.97837900	-7.41173100	1.57056300

H	-2.44045900	-5.70782300	1.51498200

H	-3.16167600	-6.86751200	0.38186700

C	-0.86022900	-7.64104700	-0.90597100

H	-0.65316100	-8.48244500	-0.23589500

H	-1.74842800	-7.88829800	-1.49607700

H	-0.01583000	-7.54955700	-1.59571300

C	0.19235700	-6.04125400	0.73406800

H	0.40875300	-6.87112600	1.41540200

H	1.07081600	-5.90408000	0.09597800

H	0.06158000	-5.13530200	1.33484400

C	2.44378800	3.47568000	3.82321700

C	3.32909500	3.28455000	5.06950000

C	3.04173100	4.42304800	6.07764500

H	3.65981300	4.30232900	6.97380200

H	3.26621400	5.40104300	5.63995500

H	1.99504900	4.43147100	6.39556900

C	3.01131700	1.92344800	5.73214600

H	3.63350600	1.77988300	6.62202400

H	1.96597800	1.85576400	6.04708000

H	3.20819100	1.09383200	5.04504000

C	4.83149900	3.31231200	4.73056200

H	5.41692800	3.16797400	5.64359700

H	5.10773400	2.51335300	4.03392200

H	5.13519800	4.26987100	4.29511200

1 23 1.0 41 1.0

2 7 1.0 47 1.0

3 31 1.0 49 1.0

4 15 1.0 46 1.0

5 13 1.0 44 1.0

6 24 1.0 50 1.0

7 9 1.5 14 1.5

8 11 1.5 56 1.0 100 1.5

9 12 1.5 21 1.0

10 22 1.5 57 1.0 98 1.5

11 13 1.5 29 1.0

12 30 1.5 87 1.0

13 28 1.5

14 17 1.5 35 1.0

15 26 1.5 32 1.5

16 27 1.5 31 1.5 39 1.0

17 30 1.5 58 1.0

18 21 1.0 24 1.5 43 1.5

19 23 1.5 25 1.5 39 1.0

20 26 1.5 59 1.0 99 1.5

21 60 1.0 61 1.0

22 23 1.5 35 1.0

23

24 33 1.5

25 86 1.0 98 1.5

26 29 1.0

27 62 1.0 212 1.5

28 34 1.5 37 1.0

29 63 1.0 84 1.0

30 48 1.0

31 36 1.5

32 38 1.5 45 1.0

33 37 1.0 42 1.5

34 64 1.0 100 1.5

35 65 1.0 85 1.0

36 40 1.5 45 1.0

37 66 1.0 67 1.0

38 68 1.0 99 1.5

39 69 1.0 70 1.0

40 71 1.0 212 1.5

41 72 1.0 73 1.0 91 1.0

42 74 1.0 198 1.5

43 75 1.0 198 1.5

44 76 1.0 77 1.0 90 1.0

45 78 1.0 79 1.0

46 80 1.0 81 1.0 89 1.0

47 82 1.0 83 1.0 88 1.0

48 113 1.0 114 1.0 115 1.0

49 152 1.0 153 1.0 154 1.0

50 155 1.0 156 1.0 157 1.0

51 52 1.0 55 1.0 95 1.0 96 1.0

52 53 1.0 92 1.0 97 1.0

53 54 1.0 93 1.0 94 1.0

54 158 1.5 162 1.5

55 168 1.5 169 1.5

56

57

58

59

60

61

62

63

64

65

66

67

68

69

70

71

72

73

74

75

76

77

78

79

80

81

82

83

84

85

86

87

88

89

90

91

92

93

94

95

96

97

98 106 1.0

99 112 1.0

100 103 1.0

101 103 1.0 116 1.0 117 1.0 140 1.0

102 103 1.0 118 1.0 119 1.0 141 1.0

103 104 1.0

104 120 1.0 121 1.0 142 1.0

105 106 1.0 122 1.0 123 1.0 146 1.0

106 107 1.0 108 1.0

107 124 1.0 125 1.0 147 1.0

108 126 1.0 127 1.0 148 1.0

109 112 1.0 130 1.0 131 1.0 143 1.0

110 112 1.0 132 1.0 133 1.0 144 1.0

111 112 1.0 128 1.0 129 1.0 145 1.0

112

113 136 1.0 137 1.0 149 1.0

114 138 1.0 139 1.0 150 1.0

115 134 1.0 135 1.0 151 1.0

116

117

118

119

120

121

122

123

124

125

126

127

128

129

130

131

132

133

134

135

136

137

138

139

140

141

142

143

144

145

146

147

148

149

150

151

152

153

154

155

156

157

158 159 1.5 163 1.0

159 160 1.5 164 1.0

160 161 1.5 179 1.0

161 162 1.5 165 1.0

162 166 1.0

163

164

165

166

167 168 1.5 171 1.5 172 1.0

168 173 1.0

169 170 1.5 174 1.0

170 171 1.5 175 1.0

171 190 1.0

172

173

174

175

176 177 1.5 181 1.5 182 1.0

177 178 1.5 183 1.0

178 179 1.5 184 1.0

179 180 1.5

180 181 1.5 185 1.0

181 186 1.0

182

183

184

185

186

187 188 1.5 192 1.5 193 1.0

188 189 1.5 194 1.0

189 190 1.5 195 1.0

190 191 1.5

191 192 1.5 196 1.0

192 197 1.0

193

194

195

196

197

198 199 1.0

199 200 1.0 204 1.0 208 1.0

200 201 1.0 202 1.0 203 1.0

201

202

203

204 205 1.0 206 1.0 207 1.0

205

206

207

208 209 1.0 210 1.0 211 1.0

209

210

211

212 213 1.0

213 214 1.0 218 1.0 222 1.0

214 215 1.0 216 1.0 217 1.0

215

216

217

218 219 1.0 220 1.0 221 1.0

219

220

221

222 223 1.0 224 1.0 225 1.0

223

224

225

___________________________

**0 imaginary frequency**

**Energy: -4312.8194 Hartree**

(*endo-butyl*)-**3d^+^**⊂**1a**

**Figure S50.** DFT-optimized structure of the (*endo-butyl*)-**3d^+^**⊂**1a** complex.

O	-1.15464100	-3.61925500	2.27558100

O	-3.05004200	-0.26414900	2.62626400

O	2.53560900	-3.81820700	2.17864100

O	3.53505700	0.46818000	2.09055800

O	2.10066600	4.16644300	2.16630600

O	-1.59224400	3.12575700	2.35907300

C	-4.14606700	-0.32930900	1.76790500

C	3.19780400	3.04533400	-1.16440200

H	4.07188400	2.58032400	-1.60441700

C	-4.79934700	0.86154700	1.40082700

C	-3.44634800	-3.83586200	-0.60264000

H	-4.44661700	-3.51748200	-0.86384600

C	3.21893200	3.29893400	0.21352900

C	-5.94519900	0.75977700	0.60668500

C	2.10027500	3.91269400	0.80110600

C	-4.58907000	-1.58042300	1.31149600

C	4.63607200	0.44898100	1.22228300

C	1.67325100	-4.13023900	-0.05893700

C	-5.74649000	-1.62105700	0.52062500

H	-6.12109900	-2.59271900	0.22111900

C	-3.55738900	3.10659600	0.93314700

C	-0.85714200	-4.62092800	0.10301300

C	6.27110200	1.63718500	-0.08074500

H	6.65502300	2.58574400	-0.43908000

C	-4.31359600	2.21184300	1.91558300

H	-5.18830100	2.77245600	2.26898400

H	-3.66965600	2.04194700	2.77517100

C	-2.97451300	-3.52866000	0.68124900

C	-1.67143500	-3.92940900	1.02144300

C	-2.68726800	-4.55008000	-1.53409700

C	-2.26127000	3.56400800	1.21405700

C	-1.39597100	-4.93735700	-1.14636200

C	5.12979400	1.66452000	0.73159400

C	1.81002400	-3.87522500	-1.42670600

H	1.08614700	-4.31745500	-2.10189000

C	0.96700700	4.24420500	0.03315500

C	6.92652500	0.44655800	-0.41540500

C	4.45109000	2.98099800	1.05613200

H	4.15394100	3.01234600	2.10529900

C	-6.44940400	-0.46871700	0.15455200

C	2.62500500	-3.56993700	0.81289900

C	5.22515400	-0.77647500	0.87546400

C	-1.59796500	4.45985400	0.35387000

C	0.99345200	3.96428200	-1.33558300

H	0.12213500	4.22909600	-1.92514200

C	-3.87807200	-2.86283300	1.72020900

H	-3.27118800	-2.66466900	2.60071200

C	3.64604000	-2.72793000	0.33627300

C	-0.20703200	4.97952900	0.67570700

H	-0.04862100	4.96947000	1.75241600

H	-0.16016800	6.02889300	0.35722400

C	6.37034700	-0.74513400	0.07210300

H	6.84143300	-1.69352700	-0.17073000

C	0.56191000	-5.04058400	0.46334900

H	0.65931200	-5.10262500	1.54543800

H	0.73042300	-6.04780600	0.06251700

C	3.73711600	-2.50967100	-1.04405600

H	4.53196200	-1.86987800	-1.40448700

C	-1.41868100	-4.59443900	3.28942600

H	-0.99891100	-5.57221300	3.02434500

H	-0.94231900	-4.22910400	4.20129900

C	-2.26964000	4.89750000	-0.78898900

H	-1.77157500	5.62267400	-1.42639900

C	2.10328100	3.38359900	-1.96941600

C	-3.56116600	4.45944900	-1.12052400

C	2.84542700	-3.09217700	-1.95351500

C	-4.17448800	3.56280000	-0.24169500

H	-5.17551100	3.21193300	-0.45519600

C	2.64225600	5.43995200	2.53483000

H	2.06404700	6.26201800	2.09659800

H	3.68815300	5.53768000	2.22120500

C	4.64078400	-2.10388400	1.31814200

H	5.47004200	-2.80601700	1.47543100

H	4.13952200	-2.00341100	2.28217200

C	3.91206400	0.60082900	3.47684000

H	4.56328400	1.46859700	3.61633100

H	2.99336800	0.73186400	4.04717400

C	-3.41460100	-0.37154200	4.01044900

H	-4.07575800	0.45048600	4.30931500

H	-3.92522200	-1.31942800	4.21484900

H	5.18606900	3.78395600	0.91803100

H	-4.64358800	-3.59354500	2.01316300

H	-0.78929800	-5.52234900	-1.83137200

C	3.38586300	2.51002800	-4.00489100

C	1.98229500	4.57161700	-4.18135200

C	2.09867400	3.18713800	-3.49823800

H	4.27590700	3.10850400	-3.78636500

H	3.52525000	1.51639200	-3.56568600

H	2.82280800	5.21665300	-3.90635000

H	1.05924500	5.08717100	-3.90018600

C	0.89556300	2.31243600	-3.92022300

H	-0.06004000	2.75525400	-3.62500500

H	0.95729500	1.31479900	-3.47174600

C	-3.24735000	-6.47656800	-3.05839700

C	-3.22114800	-4.93500600	-2.92706600

C	-4.64747400	-4.40803900	-3.17394900

H	-2.25186500	-6.91427600	-2.93862000

H	-3.90180100	-6.92332200	-2.30317500

C	-2.29676100	-4.34450400	-4.01810100

H	-5.36502800	-4.82259500	-2.45839600

H	-4.69487800	-3.31545100	-3.11643700

H	-2.25436100	-3.25250300	-3.94751500

H	-2.66843600	-4.60563000	-5.01473600

C	9.35435000	-0.21530600	-0.43784000

C	7.96442000	-0.45345700	-2.53162000

C	8.64968700	1.81135300	-1.72419300

H	7.88892000	2.30028500	-2.34175300

C	8.20756500	0.40768600	-1.26949900

H	8.87609900	2.46436800	-0.87515100

H	9.55516500	0.37734400	0.46036700

H	9.12089200	-1.23593000	-0.12047000

H	7.69768500	-1.48408000	-2.27909000

H	7.15756700	-0.03669200	-3.14342600

C	-8.13978100	-1.94301900	-1.07959900

C	-8.90466800	0.10395700	0.13368500

C	-7.54059000	0.30991700	-1.98056700

H	-7.30958800	1.35843000	-1.76992500

C	-7.74195700	-0.50865700	-0.68318000

H	-8.45113900	0.28832900	-2.58886100

H	-7.36898700	-2.42890700	-1.68714900

H	-8.33477600	-2.57053000	-0.20385100

H	-8.70831500	1.14438500	0.40900300

H	-9.07554000	-0.45972300	1.05642100

H	-6.48535000	1.67058300	0.36778800

H	-2.48907900	-0.32424500	4.58467500

H	4.43390200	-0.29810800	3.81968100

H	2.58580300	5.49867300	3.62359400

H	-2.49474700	-4.71134500	3.46338200

C	3.30304100	-4.94182100	2.62651800

C	-1.82365700	3.94423400	3.51331600

H	-1.17635600	3.56533700	4.30677500

H	-1.57334100	4.99236900	3.31636700

H	-2.86887500	3.88740000	3.83795100

H	3.15112100	-5.01446900	3.70521800

H	2.96442500	-5.86855600	2.14886100

H	4.37077500	-4.80614600	2.42008100

H	3.33354500	2.38585700	-5.09072400

H	1.98270000	4.45963100	-5.27089600

H	0.87966600	2.18909000	-5.00829300

H	10.27430100	-0.25436600	-1.03065000

H	8.87028000	-0.48897900	-3.14589100

H	9.55918800	1.73200700	-2.32701500

H	-3.62084900	-6.76792700	-4.04598000

H	-4.97890400	-4.69967800	-4.17515900

H	-1.27493400	-4.72793900	-3.93800900

H	-9.05781900	-1.91809300	-1.67440600

H	-9.83055400	0.08552200	-0.45109400

H	-6.72193600	-0.10054900	-2.58088100

C	2.98238300	-2.92525600	-3.47977500

C	-4.24987900	4.99073900	-2.39184700

C	-3.39527300	4.63110200	-3.63050300

H	-3.87279800	5.00332100	-4.54314100

H	-2.39550700	5.07307000	-3.57894800

H	-3.28039700	3.54631500	-3.72698200

C	-4.38956100	6.52888700	-2.29553100

H	-3.41820200	7.02318900	-2.20082000

H	-4.87536400	6.92295300	-3.19454000

H	-4.99597100	6.81432700	-1.43002500

C	-5.65543200	4.39295400	-2.59057900

H	-6.09938900	4.79449700	-3.50646200

H	-5.62577600	3.30284700	-2.69040500

H	-6.32730800	4.64594400	-1.76386000

C	1.70863100	-2.26587100	-4.05733500

H	1.78357200	-2.18302600	-5.14688500

H	0.80710800	-2.84219700	-3.83009900

H	1.57404800	-1.25585000	-3.65495400

C	4.19188800	-2.05429400	-3.86768900

H	4.24881800	-1.96804400	-4.95701200

H	4.11406000	-1.04029700	-3.46114400

H	5.13550200	-2.48849000	-3.52255200

C	3.16976100	-4.31959900	-4.12573000

H	3.27894000	-4.22356800	-5.21126800

H	4.06545500	-4.81460600	-3.73732200

H	2.31596200	-4.97633900	-3.93517000

C	0.17747400	-0.76268800	2.17308800

N	0.78712200	0.33719100	1.32216100

C	0.93218800	0.00056900	-0.15008000

H	0.24158100	1.19847000	1.43452200

H	1.74846500	0.53303000	1.66749900

C	-2.61738700	0.19256100	-1.81285700

C	1.41048600	-1.39186100	5.71669900

C	1.15084000	-1.55794300	4.35450700

C	0.47584100	-0.56074500	3.63886900

C	0.06422200	0.60364300	4.30406100

C	0.34050200	0.77305500	5.66176900

C	1.01207300	-0.22568000	6.37162200

H	1.93043900	-2.17154600	6.26543500

H	1.47412200	-2.45595700	3.83606800

H	-0.48362700	1.36984000	3.76176000

H	0.02258900	1.67876400	6.16994300

H	1.21913800	-0.09557400	7.42944800

H	0.59390400	-1.70436200	1.81886100

H	-0.89737400	-0.75929100	1.98631300

C	-0.35359200	-0.43581200	-0.84342900

H	1.68473500	-0.78930700	-0.19115500

H	1.34903800	0.89944300	-0.61165000

C	-1.46371200	0.61891500	-0.90042700

H	-0.05261400	-0.70139200	-1.86440100

H	-0.73084800	-1.36494000	-0.40189300

H	-1.85815100	0.80785900	0.10513500

H	-1.04794400	1.57110400	-1.25244400

H	-3.41024600	0.94474800	-1.81386300

H	-3.05488000	-0.75402200	-1.48010000

H	-2.27263000	0.06038000	-2.84496600

1 29 1.0 59 1.0

2 7 1.0 78 1.0

3 41 1.0 129 1.0

4 17 1.0 75 1.0

5 15 1.0 69 1.0

6 31 1.0 130 1.0

7 10 1.5 16 1.5

8 9 1.0 13 1.5 64 1.5

9

10 14 1.5 25 1.0

11 12 1.0 28 1.5 30 1.5

12

13 15 1.5 38 1.0

14 40 1.5 124 1.0

15 36 1.5

16 19 1.5 46 1.0

17 33 1.5 42 1.5

18 34 1.5 41 1.5 54 1.0

19 20 1.0 40 1.5

20

21 25 1.0 31 1.5 67 1.5

22 29 1.5 32 1.5 54 1.0

23 24 1.0 33 1.5 37 1.5

24

25 26 1.0 27 1.0

26

27

28 29 1.5 46 1.0

29

30 32 1.5 95 1.0

31 43 1.5

32 83 1.0

33 38 1.0

34 35 1.0 66 1.5

35

36 44 1.5 49 1.0

37 52 1.5 108 1.0

38 39 1.0 81 1.0

39

40 118 1.0

41 48 1.5

42 52 1.5 72 1.0

43 49 1.0 62 1.5

44 45 1.0 64 1.5

45

46 47 1.0 82 1.0

47

48 57 1.5 72 1.0

49 50 1.0 51 1.0

50

51

52 53 1.0

53

54 55 1.0 56 1.0

55

56

57 58 1.0 66 1.5

58

59 60 1.0 61 1.0 128 1.0

60

61

62 63 1.0 65 1.5

63

64 86 1.0

65 67 1.5 150 1.0

66 149 1.0

67 68 1.0

68

69 70 1.0 71 1.0 127 1.0

70

71

72 73 1.0 74 1.0

73

74

75 76 1.0 77 1.0 126 1.0

76

77

78 79 1.0 80 1.0 125 1.0

79

80

81

82

83

84 86 1.0 87 1.0 88 1.0 137 1.0

85 86 1.0 89 1.0 90 1.0 138 1.0

86 91 1.0

87

88

89

90

91 92 1.0 93 1.0 139 1.0

92

93

94 95 1.0 97 1.0 98 1.0 143 1.0

95 96 1.0 99 1.0

96 100 1.0 101 1.0 144 1.0

97

98

99 102 1.0 103 1.0 145 1.0

100

101

102

103

104 108 1.0 110 1.0 111 1.0 140 1.0

105 108 1.0 112 1.0 113 1.0 141 1.0

106 107 1.0 108 1.0 109 1.0 142 1.0

107

108

109

110

111

112

113

114 118 1.0 120 1.0 121 1.0 146 1.0

115 118 1.0 122 1.0 123 1.0 147 1.0

116 117 1.0 118 1.0 119 1.0 148 1.0

117

118

119

120

121

122

123

124

125

126

127

128

129 134 1.0 135 1.0 136 1.0

130 131 1.0 132 1.0 133 1.0

131

132

133

134

135

136

137

138

139

140

141

142

143

144

145

146

147

148

149 163 1.0 167 1.0 171 1.0

150 151 1.0 155 1.0 159 1.0

151 152 1.0 153 1.0 154 1.0

152

153

154

155 156 1.0 157 1.0 158 1.0

156

157

158

159 160 1.0 161 1.0 162 1.0

160

161

162

163 164 1.0 165 1.0 166 1.0

164

165

166

167 168 1.0 169 1.0 170 1.0

168

169

170

171 172 1.0 173 1.0 174 1.0

172

173

174

175 176 1.0 183 1.0 192 1.0 193 1.0

176 177 1.0 178 1.0 179 1.0

177 194 1.0 195 1.0 196 1.0

178

179

180 197 1.0 202 1.0 203 1.0 204 1.0

181 182 1.5 186 1.5 187 1.0

182 183 1.5 188 1.0

183 184 1.5

184 185 1.5 189 1.0

185 186 1.5 190 1.0

186 191 1.0

187

188

189

190

191

192

193

194 197 1.0 198 1.0 199 1.0

195

196

197 200 1.0 201 1.0

198

199

200

201

202

203

204

___________________________

**0 imaginary frequency**

**Energy: -3737.5896 Hartree**

(*endo-butyl*)-**3e^+^**⊂**1a**

**Figure S51.** DFT-optimized structure of the (*endo-butyl*)-**3e^+^**⊂**1a** complex.

O	2.36552800	-2.73608200	3.22512500

O	-1.60323000	-2.26053100	3.17208300

O	3.91657300	0.68003300	1.74091300

O	2.50263200	4.14278800	0.51781700

O	-1.44153300	3.39597300	0.86028900

O	-3.26723000	0.69724100	2.21072800

C	-2.06581000	-3.11673000	2.16788200

C	-1.73437100	4.18275900	-2.72657000

H	-1.11391200	4.60838000	-3.50676600

C	-3.37880900	-2.97985700	1.69238100

C	1.45030700	-4.18321900	-0.03034300

H	0.62423700	-4.72533100	-0.47518200

C	-1.17473700	4.05221300	-1.44819500

C	-3.81787400	-3.85687400	0.69029100

H	-4.83274500	-3.74605200	0.33018900

C	-1.99000500	3.55632100	-0.41984100

C	-1.20716300	-4.11435600	1.66439700

C	2.51100600	3.47287800	-0.70462200

C	5.40584800	-1.02229300	0.89914000

C	-1.71429400	-4.98696400	0.69774700

H	-1.07653700	-5.79789200	0.36011000

C	-4.88380700	-0.89574500	1.37257100

C	3.57228100	-2.78988800	1.13461000

C	1.41197000	2.87603700	-2.76092400

H	0.54938800	2.95648600	-3.41359400

C	-4.34712600	-1.97073000	2.30664700

H	-5.20350000	-2.52702100	2.70592600

H	-3.85210900	-1.49580300	3.15031000

C	1.36418100	-3.83623400	1.32062500

C	2.44311400	-3.13408100	1.89379400

C	2.56823700	-3.87612400	-0.82029500

C	-4.33441300	0.39753800	1.35235000

C	3.60979700	-3.16988300	-0.21208600

H	4.48474300	-2.89665800	-0.78775300

C	1.38821600	3.57071700	-1.54968700

C	6.48153100	-1.36193300	0.06561100

H	6.86215600	-2.37573300	0.12418600

C	-3.33355300	3.20643400	-0.64481100

C	2.51599400	2.11123100	-3.16942900

C	0.24664400	4.51576400	-1.18048300

H	0.36036400	4.76370800	-0.12693900

C	-3.01418000	-4.87917400	0.18032600

C	4.92841300	0.29236600	0.86405000

C	3.62064800	2.69119400	-1.05725400

C	-4.82634200	1.39176500	0.49565100

C	-3.82882200	3.33536100	-1.94562500

H	-4.86619600	3.07456400	-2.12482400

C	0.20219000	-4.29028300	2.20515700

H	0.29356200	-3.76309800	3.15246600

C	5.45254300	1.23300300	-0.03567700

C	-4.25492900	2.80351400	0.50642600

H	-3.71729700	2.94998300	1.43937500

H	-5.09860000	3.50409100	0.50986200

C	3.59872000	2.02504700	-2.28912600

H	4.46329400	1.42896600	-2.55285200

C	-3.50973600	-5.88605500	-0.87498900

C	6.51968000	0.84577000	-0.85011400

H	6.93702300	1.58674300	-1.52652800

C	2.94591000	-3.65285500	4.15905100

H	2.82575800	-3.20828600	5.14923300

H	2.43852900	-4.62472600	4.13361900

C	-5.91332500	1.07054400	-0.32955300

H	-6.32063000	1.85321600	-0.95790100

C	-3.05469100	3.81745600	-3.01068800

C	-6.50731200	-0.19488800	-0.33489900

C	7.07171900	-0.44458400	-0.81057900

C	-5.96764300	-1.15799100	0.53080500

H	-6.42313000	-2.14224600	0.57779000

C	-1.59276900	4.54044300	1.71889400

H	-1.12937500	4.28315500	2.67135300

H	-2.65017400	4.77698000	1.87396700

C	2.63170700	-4.35449500	-2.28397600

C	-7.72060600	-0.54634800	-1.21637400

C	-3.67910200	3.95959400	-4.41181600

C	1.41692200	-3.81380900	-3.07217300

H	0.46755900	-4.15506200	-2.64876900

H	1.45533500	-4.15613300	-4.11189800

H	1.40923100	-2.71887800	-3.07738300

C	3.09123800	5.44884400	0.48492100

H	4.14411500	5.40444500	0.18526600

H	2.55360900	6.11033700	-0.20429500

C	2.60679000	-5.90198200	-2.31094700

H	3.46016500	-6.31744000	-1.76568500

H	2.65301400	-6.26670200	-3.34282400

H	1.69470500	-6.30147700	-1.85723000

C	-4.16205600	2.57343600	-4.90079300

H	-4.61879100	2.65882300	-5.89241800

H	-4.90898600	2.13999500	-4.22876000

H	-3.32666400	1.86899400	-4.97360900

C	-1.83629300	-2.76549500	4.49451500

H	-2.90736600	-2.88861500	4.69113000

H	-1.33804100	-3.72888800	4.64775400

C	-4.95546600	-5.60025700	-1.32327800

H	-5.66353500	-5.67517900	-0.49147200

H	-5.25829100	-6.33403700	-2.07618000

H	-5.05534500	-4.60709600	-1.77398800

C	-2.59792900	-5.82018800	-2.12302000

H	-2.61192800	-4.81967600	-2.56817700

H	-2.94001600	-6.53233300	-2.88140100

H	-1.55951800	-6.06895300	-1.88397100

C	3.91254500	-3.88359700	-2.99875800

H	3.90715500	-4.23723700	-4.03431800

H	4.81523900	-4.27940400	-2.52266800

H	3.98895500	-2.79129200	-3.02187600

C	2.52301800	1.45369500	-4.56346200

C	-3.46244900	-7.31461400	-0.28174500

H	-2.44931600	-7.60176300	0.01469000

H	-3.81001900	-8.04384000	-1.02117800

H	-4.10489100	-7.39468700	0.60092900

C	-4.88456000	4.92755900	-4.34290000

H	-4.57288800	5.91961600	-4.00103000

H	-5.66120400	4.56738500	-3.66176400

H	-5.33850800	5.03784600	-5.33329600

C	-2.67937200	4.51165600	-5.44541800

H	-2.32680400	5.51356200	-5.18013400

H	-3.16526700	4.58504900	-6.42284800

H	-1.80763600	3.85894500	-5.56154800

C	-8.91117700	-0.95119200	-0.31454000

H	-9.19690600	-0.13052700	0.35115500

H	-8.67753900	-1.82060400	0.30699200

H	-9.78089800	-1.20777700	-0.92845000

C	-8.16633000	0.63568500	-2.09758300

H	-8.47741000	1.49850500	-1.49965700

H	-9.02348800	0.33565600	-2.70752200

H	-7.37413000	0.95690600	-2.78237600

C	3.80586500	0.64126500	-4.82204300

H	3.76064000	0.18944700	-5.81766300

H	3.92945500	-0.16943900	-4.09627800

H	4.70135700	1.26959900	-4.78826600

C	1.31533500	0.50082000	-4.70956200

H	0.36250400	1.01945500	-4.56612500

H	1.37161600	-0.31389700	-3.97996900

H	1.30083900	0.05464800	-5.70969800

C	-7.35535900	-1.72842500	-2.14563700

H	-8.20868800	-1.98887600	-2.78058400

H	-7.08062600	-2.62371900	-1.57991300

H	-6.51418600	-1.47071000	-2.79762100

C	2.42914900	2.56105200	-5.64134900

H	1.50901000	3.14536700	-5.54531700

H	2.44028000	2.11919400	-6.64357800

H	3.27370800	3.25331400	-5.56664300

H	0.39506400	5.45067800	-1.73732000

C	8.27345600	-0.79625100	-1.70742000

C	7.88945300	-0.59032300	-3.19196400

C	9.46311900	0.12788500	-1.35348500

C	8.72982200	-2.25638700	-1.52831500

H	9.58458500	-2.45684000	-2.18129100

H	9.04681600	-2.46254300	-0.50090200

H	7.94064100	-2.96759700	-1.79417100

H	8.73686100	-0.83552400	-3.84103700

H	7.05012700	-1.23485500	-3.47370400

H	7.60313600	0.44482100	-3.40072600

H	10.32648100	-0.10540900	-1.98573100

H	9.21855700	1.18368500	-1.50268200

H	9.76336200	-0.00167100	-0.30876400

H	0.34548800	-5.35781800	2.42031300

H	-1.42425500	-2.03556200	5.19472800

H	4.01260200	-3.80636100	3.95998900

H	3.02236100	5.84988000	1.49823200

H	-1.09361400	5.41719800	1.29454600

C	-3.67743500	1.18437100	3.49902900

H	-2.76683900	1.39208000	4.06335100

H	-4.26560000	2.10282300	3.40157300

H	-4.27648500	0.43866600	4.03197800

C	4.43364000	1.15335800	2.99223000

H	5.05954800	2.04262300	2.85223600

H	3.57648800	1.40927500	3.61603000

H	5.03017200	0.38248200	3.49305900

C	4.84605800	2.61729700	-0.14849600

H	5.61194300	3.30133900	-0.53581100

H	4.55963200	2.98833300	0.83607100

C	4.75160800	-2.06581900	1.78231300

H	5.50939000	-2.81003200	2.05992800

H	4.39774600	-1.61456800	2.70943100

C	0.67454800	0.45752700	2.27826500

N	-0.49939400	0.61348200	1.33461200

H	-1.37571600	0.30372800	1.79176000

C	1.01932900	3.54036500	4.49040400

C	1.08994900	2.69123400	3.38702900

C	0.57770400	1.38607700	3.46421500

C	0.00253800	0.94077300	4.66290300

C	-0.07590600	1.77952200	5.77239600

C	0.43334500	3.07048000	5.66312300

H	1.41456100	4.54811000	4.46165300

H	1.55632800	3.04443100	2.46989400

H	-0.37363700	-0.07528400	4.72591800

H	-0.50886800	1.45483000	6.71026800

H	0.07688700	-1.14069600	0.32985000

H	0.48986500	0.37007400	-0.50348200

H	1.58242300	0.65229900	1.70238700

H	0.68207400	-0.58793300	2.59212500

H	-0.66987300	1.61656300	1.13318400

N	0.35517700	3.96963300	6.83282100

O	-0.16598900	3.52521300	7.85274700

O	0.81202400	5.10312900	6.70505300

C	-0.29730200	-0.15343500	0.04608300

C	-1.58361300	-0.26521700	-0.76297900

H	-2.34834600	-0.76546700	-0.15922800

H	-1.96394600	0.73420500	-1.00222000

C	-1.37332300	-1.06024700	-2.05867000

H	-0.61142800	-0.56444700	-2.67154000

H	-0.97893900	-2.05375800	-1.81357200

C	-2.66740900	-1.20487500	-2.86467100

H	-2.49577700	-1.77138200	-3.78487900

H	-3.43572000	-1.72760300	-2.28494200

H	-3.07135900	-0.22587000	-3.14415800

1 30 1.0 59 1.0

2 7 1.0 90 1.0

3 43 1.0 165 1.0

4 18 1.0 79 1.0

5 16 1.0 69 1.0

6 32 1.0 161 1.0

7 10 1.5 17 1.5

8 9 1.0 13 1.5 64 1.5

9

10 14 1.5 26 1.0

11 12 1.0 29 1.5 31 1.5

12

13 16 1.5 40 1.0

14 15 1.0 42 1.5

15

16 38 1.5

17 20 1.5 48 1.0

18 35 1.5 44 1.5

19 36 1.5 43 1.5 172 1.0

20 21 1.0 42 1.5

21

22 26 1.0 32 1.5 67 1.5

23 30 1.5 33 1.5 172 1.0

24 25 1.0 35 1.5 39 1.5

25

26 27 1.0 28 1.0

27

28

29 30 1.5 48 1.0

30

31 33 1.5 72 1.0

32 45 1.5

33 34 1.0

34

35 40 1.0

36 37 1.0 66 1.5

37

38 46 1.5 51 1.0

39 54 1.5 105 1.0

40 41 1.0 142 1.0

41

42 56 1.0

43 50 1.5

44 54 1.5 169 1.0

45 51 1.0 62 1.5

46 47 1.0 64 1.5

47

48 49 1.0 156 1.0

49

50 57 1.5 169 1.0

51 52 1.0 53 1.0

52

53

54 55 1.0

55

56 93 1.0 97 1.0 106 1.0

57 58 1.0 66 1.5

58

59 60 1.0 61 1.0 158 1.0

60

61

62 63 1.0 65 1.5

63

64 74 1.0

65 67 1.5 73 1.0

66 143 1.0

67 68 1.0

68

69 70 1.0 71 1.0 160 1.0

70

71

72 75 1.0 82 1.0 101 1.0

73 118 1.0 122 1.0 134 1.0

74 86 1.0 110 1.0 114 1.0

75 76 1.0 77 1.0 78 1.0

76

77

78

79 80 1.0 81 1.0 159 1.0

80

81

82 83 1.0 84 1.0 85 1.0

83

84

85

86 87 1.0 88 1.0 89 1.0

87

88

89

90 91 1.0 92 1.0 157 1.0

91

92

93 94 1.0 95 1.0 96 1.0

94

95

96

97 98 1.0 99 1.0 100 1.0

98

99

100

101 102 1.0 103 1.0 104 1.0

102

103

104

105 126 1.0 130 1.0 138 1.0

106 107 1.0 108 1.0 109 1.0

107

108

109

110 111 1.0 112 1.0 113 1.0

111

112

113

114 115 1.0 116 1.0 117 1.0

115

116

117

118 119 1.0 120 1.0 121 1.0

119

120

121

122 123 1.0 124 1.0 125 1.0

123

124

125

126 127 1.0 128 1.0 129 1.0

127

128

129

130 131 1.0 132 1.0 133 1.0

131

132

133

134 135 1.0 136 1.0 137 1.0

135

136

137

138 139 1.0 140 1.0 141 1.0

139

140

141

142

143 144 1.0 145 1.0 146 1.0

144 150 1.0 151 1.0 152 1.0

145 153 1.0 154 1.0 155 1.0

146 147 1.0 148 1.0 149 1.0

147

148

149

150

151

152

153

154

155

156

157

158

159

160

161 162 1.0 163 1.0 164 1.0

162

163

164

165 166 1.0 167 1.0 168 1.0

166

167

168

169 170 1.0 171 1.0

170

171

172 173 1.0 174 1.0

173

174

175 176 1.0 180 1.0 190 1.0 191 1.0

176 177 1.0 192 1.0 196 1.0

177

178 179 1.5 183 1.5 184 1.0

179 180 1.5 185 1.0

180 181 1.5

181 182 1.5 186 1.0

182 183 1.5 187 1.0

183 193 1.0

184

185

186

187

188 196 1.0

189 196 1.0

190

191

192

193 194 1.5 195 1.5

194

195

196 197 1.0

197 198 1.0 199 1.0 200 1.0

198

199

200 201 1.0 202 1.0 203 1.0

201

202

203 204 1.0 205 1.0 206 1.0

204

205

206

___________________________

**0 imaginary frequency**

**Energy: -3942.0879 Hartree**

(*endo-p-nitro-benzyl* )- **3e^+^**⊂**1a**

**Figure S52.** DFT-optimized structure of the (*endo-p-nitro-benzyl* )- **3e^+^**⊂**1a** complex.

O	1.51254600	-3.59928500	2.52077700

O	-2.21948800	-4.21149800	2.24514500

O	2.85055400	-0.22875500	3.04575000

O	0.54198200	2.96328600	2.52659500

O	-2.90297100	3.64884000	2.61165500

O	-3.85880000	-0.08931900	2.30908300

C	-2.21578900	-3.91824500	0.88710900

C	-1.98577900	4.23992900	-0.90415300

H	-1.20600300	4.75364000	-1.45871000

C	-3.27125000	-3.15826400	0.35269400

C	2.03732700	-5.05277600	-0.81442500

H	1.50884100	-5.70966800	-1.49949900

C	-1.93347400	4.28896000	0.49153900

C	-3.25221200	-2.86076400	-1.01579700

H	-4.07168900	-2.28100500	-1.42034200

C	-2.95053100	3.64482700	1.22122600

C	-1.14327700	-4.34629100	0.08304100

C	1.27295600	3.70954800	1.58937700

C	4.52197500	-1.15702100	1.56800900

C	-1.17954900	-4.03004300	-1.27870800

H	-0.35959700	-4.37028000	-1.90177700

C	-5.18010700	-1.49186200	0.83515400

C	3.39702900	-3.42634800	1.00975000

C	1.29725700	5.43111900	-0.08960800

H	0.77805700	6.22850600	-0.61377300

C	-4.42790400	-2.73685400	1.26123900

H	-5.14790800	-3.56438000	1.30987000

H	-4.03229900	-2.62353200	2.27147200

C	1.40043800	-4.69808900	0.37731900

C	2.10957400	-3.90345500	1.30072400

C	3.33602300	-4.62605700	-1.13612100

C	-4.85564700	-0.21801300	1.32752400

C	3.98126300	-3.80135400	-0.20972400

H	4.97996200	-3.44466800	-0.42482100

C	0.59385400	4.72113200	0.88428600

C	5.59371700	-0.92736400	0.70031500

H	6.17779600	-1.78108000	0.37393300

C	-3.98826500	2.96249500	0.56469300

C	2.64638400	5.17544700	-0.38033300

C	-0.84799000	5.08997000	1.20084900

H	-1.02154600	5.01524000	2.27299500

C	-2.22648400	-3.30199700	-1.86170800

C	3.82696000	-0.03908200	2.06558900

C	2.63053500	3.43440500	1.36300700

C	-5.51951600	0.93089100	0.88037400

C	-3.98992400	2.93676900	-0.83684000

H	-4.79251400	2.40227500	-1.32959600

C	-0.01028100	-5.18476900	0.67526100

H	-0.16073700	-5.22973700	1.75225700

C	4.13276800	1.26592300	1.64153600

C	-5.11728800	2.31174200	1.36136700

H	-4.81396500	2.27607800	2.40775400

H	-5.99873100	2.96293300	1.31002000

C	3.28405900	4.18587700	0.37264600

H	4.33496400	4.00018900	0.20477800

C	-2.23596400	-3.04973300	-3.38252700

C	5.21066700	1.43036100	0.75975900

H	5.49449800	2.43417900	0.47618400

C	1.80508200	-4.55695200	3.54498300

H	1.25601000	-4.24525500	4.43535100

H	1.48210100	-5.56396700	3.25572800

C	-6.56463300	0.77784900	-0.04078200

H	-7.08988000	1.67167000	-0.35830100

C	-3.00755900	3.57666500	-1.60138200

C	-6.95490900	-0.47276500	-0.53242600

C	5.97708800	0.35742600	0.29002000

C	-6.23303500	-1.58748400	-0.08102000

H	-6.49956300	-2.57769000	-0.43957800

C	-3.63684900	4.71455800	3.22997500

H	-3.51243300	4.59465600	4.30799800

H	-4.70279100	4.66462100	2.98062500

C	3.99633200	-5.08612100	-2.45032700

C	-8.12641300	-0.65774800	-1.51477900

C	-3.03855800	3.60747500	-3.14177500

C	4.07595900	-6.63138900	-2.46670200

H	4.67167900	-7.00302500	-1.62672700

H	4.54631400	-6.97579500	-3.39380400

H	3.08668000	-7.09498600	-2.40617400

C	0.54259100	3.50165300	3.85959200

H	1.50876100	3.33531300	4.34761600

H	0.33153200	4.57466100	3.85029600

C	5.42272900	-4.52792500	-2.61467600

H	5.43202000	-3.43413200	-2.64312800

H	5.84808000	-4.88448100	-3.55769300

H	6.08609400	-4.86317400	-1.81033300

C	-3.21188900	5.07252300	-3.61068200

H	-3.23740900	5.12061100	-4.70445700

H	-2.38995300	5.70874400	-3.26914400

H	-4.14568100	5.49901000	-3.23073400

C	-2.86487800	-5.44201200	2.59250900

H	-3.91887400	-5.44198100	2.29169800

H	-2.36382000	-6.29850500	2.12684100

C	-0.94825100	-2.30833400	-3.80975000

H	-0.87524900	-1.33089500	-3.32209000

H	-0.94788500	-2.14697100	-4.89298800

H	-0.04514500	-2.87290700	-3.56136700

C	-2.30557200	-4.41142700	-4.11552800

H	-3.21146500	-4.96060500	-3.83958000

H	-2.31757700	-4.25961900	-5.20016500

H	-1.44557900	-5.04445300	-3.87804700

C	3.15158800	-4.60299500	-3.65346600

H	3.60054600	-4.94502200	-4.59210000

H	3.10330300	-3.50979000	-3.68390700

H	2.12913300	-4.99270800	-3.61778200

C	3.37280300	6.00669400	-1.45507200

C	-3.44340100	-2.20324100	-3.82810200

H	-4.39534800	-2.69484500	-3.60399600

H	-3.40264400	-2.04719000	-4.91032300

H	-3.44777100	-1.21623300	-3.35263700

C	-1.71519900	3.04481000	-3.71049200

H	-1.55979700	2.00612500	-3.40056000

H	-0.84724400	3.62580400	-3.38508600

H	-1.73414100	3.07018700	-4.80497100

C	-4.19958200	2.77616500	-3.71962000

H	-4.13207600	1.72198100	-3.42985400

H	-4.17320300	2.81953100	-4.81246100

H	-5.17495200	3.15822900	-3.40161600

C	-8.77699000	0.68196700	-1.90755800

H	-8.06732800	1.35157600	-2.40496700

H	-9.19478000	1.20435700	-1.04085500

H	-9.59892700	0.49953600	-2.60604200

C	-7.61744600	-1.34328300	-2.80502100

H	-8.44279500	-1.47957800	-3.51157100

H	-6.84885600	-0.73746000	-3.29624800

H	-7.19075200	-2.33041400	-2.60360800

C	2.62940700	5.87229500	-2.80540900

H	3.12605400	6.47686300	-3.57135200

H	1.59151000	6.21260400	-2.74011000

H	2.62532200	4.83342100	-3.15260500

C	3.38641700	7.49345900	-1.02547100

H	3.90973600	7.62184300	-0.07261500

H	2.37532500	7.89527100	-0.90908000

H	3.90002100	8.10049400	-1.77837500

C	-9.20813600	-1.54635500	-0.85498100

H	-10.04850100	-1.69238100	-1.54168100

H	-9.59408400	-1.08302700	0.05864600

H	-8.81997300	-2.53464700	-0.59145600

C	4.82813800	5.54841000	-1.66549600

H	5.43224100	5.67417000	-0.76103900

H	5.29208700	6.15161100	-2.45149100

H	4.88509200	4.50058700	-1.97773400

H	-0.98118200	6.14530700	0.93502900

C	7.25980700	0.56283500	-0.54272500

C	8.47511200	0.35335400	0.39447000

C	7.34316300	-0.44838900	-1.70864600

C	7.34149200	1.97951800	-1.14502300

H	8.24033600	2.06228000	-1.76350000

H	6.47743000	2.19204400	-1.78228800

H	7.41179900	2.75508200	-0.37526500

H	9.41093800	0.49543900	-0.15705800

H	8.45982100	1.06511600	1.22648500

H	8.48438500	-0.65618400	0.81764000

H	8.26774800	-0.28303800	-2.27157700

H	7.36383300	-1.48513100	-1.35989600

H	6.50260400	-0.33869700	-2.39849400

H	-0.10623900	-6.20796200	0.29052100

H	-2.79970500	-5.53133000	3.67847700

H	2.87788000	-4.58644600	3.77042000

H	-0.24384200	2.98482200	4.41150300

H	-3.24839000	5.69338600	2.92568000

C	-4.39130300	-0.19354700	3.64283000

H	-3.55728300	-0.06451300	4.33371700

H	-5.14222100	0.58152600	3.82645500

H	-4.84591000	-1.17607600	3.80297200

C	3.40106300	-0.19268900	4.36917500

H	4.16159900	-0.97001300	4.50549300

H	3.85731900	0.78107200	4.58417500

H	2.57827800	-0.36956400	5.06332500

C	3.39502000	2.45646500	2.25535800

H	4.13097300	3.05058200	2.81492300

H	2.69329600	2.05663000	2.98308700

C	4.17268400	-2.57402400	2.01395100

H	5.10887900	-3.09522700	2.24863000

H	3.58970100	-2.52048500	2.93063500

C	-1.23212500	0.33892800	0.26861300

N	-1.09990400	0.58178500	1.76287200

H	-0.55712700	1.44711600	1.94848700

C	1.79787000	1.45074000	-1.74094200

C	0.60955500	1.47600200	-1.01881800

C	0.08043200	0.29868400	-0.47132700

C	0.75289400	-0.91494900	-0.66654000

C	1.93797500	-0.95945600	-1.39526000

C	2.43899200	0.22850100	-1.91919800

H	2.22429300	2.35081200	-2.16244900

H	0.08631900	2.41622000	-0.88884900

H	0.34243200	-1.83706600	-0.27067100

H	2.46652800	-1.88983900	-1.55158200

H	0.47196500	-0.78582300	2.15604900

H	-1.17517300	-1.41609300	2.38711100

H	-1.86463900	1.14676800	-0.10359400

H	-1.77734300	-0.60162600	0.16648000

H	-2.06613200	0.72662700	2.09969100

N	3.68732400	0.19173500	-2.69495900

O	4.14911600	-0.91263800	-2.98277000

O	4.19921300	1.26556600	-3.00990800

C	-0.51694200	-0.56221100	2.55661400

C	-0.41348500	-0.18915800	4.03470900

H	0.35995500	0.57814400	4.13556900

H	-1.35653900	0.25746000	4.37973800

C	-0.06196800	-1.38957100	4.93135900

H	0.80671200	-1.89904300	4.50119100

H	0.25072000	-0.99971700	5.90751000

C	-1.20325800	-2.39183000	5.13649100

H	-0.89755500	-3.18158100	5.83053500

H	-2.08321700	-1.90480900	5.57312700

H	-1.50489000	-2.87563900	4.20285800

1 30 1.0 59 1.0

2 7 1.0 90 1.0

3 43 1.0 165 1.0

4 18 1.0 79 1.0

5 16 1.0 69 1.0

6 32 1.0 161 1.0

7 10 1.5 17 1.5

8 9 1.0 13 1.5 64 1.5

9

10 14 1.5 26 1.0

11 12 1.0 29 1.5 31 1.5

12

13 16 1.5 40 1.0

14 15 1.0 42 1.5

15

16 38 1.5

17 20 1.5 48 1.0

18 35 1.5 44 1.5

19 36 1.5 43 1.5 172 1.0

20 21 1.0 42 1.5

21

22 26 1.0 32 1.5 67 1.5

23 30 1.5 33 1.5 172 1.0

24 25 1.0 35 1.5 39 1.5

25

26 27 1.0 28 1.0

27

28

29 30 1.5 48 1.0

30

31 33 1.5 72 1.0

32 45 1.5

33 34 1.0

34

35 40 1.0

36 37 1.0 66 1.5

37

38 46 1.5 51 1.0

39 54 1.5 105 1.0

40 41 1.0 142 1.0

41

42 56 1.0

43 50 1.5

44 54 1.5 169 1.0

45 51 1.0 62 1.5

46 47 1.0 64 1.5

47

48 49 1.0 156 1.0

49

50 57 1.5 169 1.0

51 52 1.0 53 1.0

52

53

54 55 1.0

55

56 93 1.0 97 1.0 106 1.0

57 58 1.0 66 1.5

58

59 60 1.0 61 1.0 158 1.0

60

61

62 63 1.0 65 1.5

63

64 74 1.0

65 67 1.5 73 1.0

66 143 1.0

67 68 1.0

68

69 70 1.0 71 1.0 160 1.0

70

71

72 75 1.0 82 1.0 101 1.0

73 118 1.0 122 1.0 134 1.0

74 86 1.0 110 1.0 114 1.0

75 76 1.0 77 1.0 78 1.0

76

77

78

79 80 1.0 81 1.0 159 1.0

80

81

82 83 1.0 84 1.0 85 1.0

83

84

85

86 87 1.0 88 1.0 89 1.0

87

88

89

90 91 1.0 92 1.0 157 1.0

91

92

93 94 1.0 95 1.0 96 1.0

94

95

96

97 98 1.0 99 1.0 100 1.0

98

99

100

101 102 1.0 103 1.0 104 1.0

102

103

104

105 126 1.0 130 1.0 138 1.0

106 107 1.0 108 1.0 109 1.0

107

108

109

110 111 1.0 112 1.0 113 1.0

111

112

113

114 115 1.0 116 1.0 117 1.0

115

116

117

118 119 1.0 120 1.0 121 1.0

119

120

121

122 123 1.0 124 1.0 125 1.0

123

124

125

126 127 1.0 128 1.0 129 1.0

127

128

129

130 131 1.0 132 1.0 133 1.0

131

132

133

134 135 1.0 136 1.0 137 1.0

135

136

137

138 139 1.0 140 1.0 141 1.0

139

140

141

142

143 144 1.0 145 1.0 146 1.0

144 150 1.0 151 1.0 152 1.0

145 153 1.0 154 1.0 155 1.0

146 147 1.0 148 1.0 149 1.0

147

148

149

150

151

152

153

154

155

156

157

158

159

160

161 162 1.0 163 1.0 164 1.0

162

163

164

165 166 1.0 167 1.0 168 1.0

166

167

168

169 170 1.0 171 1.0

170

171

172 173 1.0 174 1.0

173

174

175 176 1.0 180 1.0 190 1.0 191 1.0

176 177 1.0 192 1.0 196 1.0

177

178 179 1.5 183 1.5 184 1.0

179 180 1.5 185 1.0

180 181 1.5

181 182 1.5 186 1.0

182 183 1.5 187 1.0

183 193 1.0

184

185

186

187

188 196 1.0

189 196 1.0

190

191

192

193 194 1.5 195 1.5

194

195

196 197 1.0

197 198 1.0 199 1.0 200 1.0

198

199

200 201 1.0 202 1.0 203 1.0

201

202

203 204 1.0 205 1.0 206 1.0

204

205

206

___________________________

**0 imaginary frequency**

**Energy: -3942.0866 Hartree**

(*eso-p-nitro-benzyl* )-**3f^+^**⊂**1a**

**Figure S53.** DFT-optimized structure of the (*eso-p-nitro-benzyl*)-**3f^+^**⊂**1a** complex.

O	-2.45029000	3.90352100	2.19228700

O	0.90044600	2.88985100	2.13015100

O	-3.62134900	0.16256600	1.72901000

O	-2.46270900	-4.17077600	1.60810500

O	1.25014400	-3.58355200	1.83078000

O	3.01889600	-0.47116900	2.67742700

C	1.74485700	3.66145400	1.31624600

C	1.82417200	-5.36697700	-1.33474300

H	1.26892200	-6.02751300	-1.99145700

C	3.10367600	3.33723900	1.18742700

C	-1.32374700	4.43465800	-1.27155300

H	-0.48879100	4.90414000	-1.78383600

C	1.16837400	-4.87360100	-0.19935000

C	3.89672800	4.17234100	0.38146800

H	4.95813600	3.96878000	0.33483500

C	1.88789200	-4.05582100	0.68751800

C	1.18907800	4.77965900	0.66362600

C	-2.37687100	-3.81133200	0.26656100

C	-5.17148500	1.36357500	0.31444300

C	2.02087500	5.54935100	-0.15014700

H	1.59011300	6.41946100	-0.63728300

C	4.29487100	0.98835200	1.23371100

C	-3.46600500	3.27278200	0.09253200

C	-1.28236300	-3.93250800	-1.87294700

H	-0.47247700	-4.32405500	-2.47609200

C	3.73891500	2.20193200	1.98437500

H	4.56084900	2.63868600	2.56784100

H	3.00800500	1.83414800	2.70109500

C	-1.33624500	4.47364500	0.12510400

C	-2.42376100	3.89162200	0.80181600

C	-2.35272500	3.84305400	-2.02076400

C	3.91609400	-0.31232000	1.62057400

C	-3.40522800	3.25634400	-1.30763700

H	-4.21543900	2.77510700	-1.84136300

C	-1.32442200	-4.30125500	-0.52005100

C	-6.21047500	1.32842900	-0.62552000

H	-6.64231500	2.27468300	-0.93164300

C	3.22841900	-3.70769300	0.43537400

C	-2.23436000	-3.08800400	-2.45062700

C	-0.28170700	-5.24917200	0.06691000

H	-0.45735800	-5.31613400	1.13957600

C	3.38672000	5.27127100	-0.31268600

C	-4.62363100	0.14820400	0.74491100

C	-3.33299700	-2.92029400	-0.26096100

C	4.43264600	-1.45221500	0.98303600

C	3.83316800	-4.23273900	-0.71162500

H	4.87898600	-4.00482000	-0.88760100

C	-0.25032300	5.21594500	0.89118300

H	-0.48900100	5.15701800	1.95236700

C	-5.06760200	-1.07873200	0.22650300

C	4.02457400	-2.85746500	1.42452700

H	3.44060000	-2.76686500	2.33683400

H	4.93953100	-3.40881800	1.67524700

C	-3.23971200	-2.57809700	-1.61103400

H	-3.98294200	-1.90253300	-2.01921800

C	4.25814900	6.19094500	-1.18864400

C	-6.10920800	-1.05578400	-0.70709600

H	-6.46525500	-2.00923600	-1.08705800

C	-3.15982600	5.01027600	2.76453700

H	-3.10092600	4.89162800	3.84823800

H	-2.70511600	5.96580100	2.47800500

C	5.40746400	-1.26438600	-0.00566900

H	5.84756600	-2.14327400	-0.45885600

C	3.16205400	-5.06805800	-1.61634800

C	5.85790000	0.00243300	-0.38678900

C	-6.70913400	0.13142100	-1.15096400

C	5.26430000	1.10995500	0.23566100

H	5.59592200	2.10144200	-0.04542500

C	1.39913300	-4.43471900	2.97347100

H	0.82412200	-3.97787500	3.78046300

H	2.44948900	-4.51878300	3.27619700

C	-2.32005000	3.90217400	-3.56042500

C	7.01396000	0.21093900	-1.38615100

C	3.90918100	-5.64412200	-2.83500700

C	-3.49542200	3.13873800	-4.19962400

H	-3.48959200	2.07793800	-3.92724200

H	-3.42243500	3.19942300	-5.28960100

H	-4.46360500	3.56240800	-3.91388000

C	-3.23653800	-5.35035900	1.86180700

H	-4.27248300	-5.22960600	1.52546800

H	-2.80041700	-6.22350600	1.36365700

C	-1.00367800	3.28945000	-4.09117600

H	-0.12294100	3.82157600	-3.72021900

H	-0.98002100	3.33913100	-5.18489000

H	-0.90696300	2.23939300	-3.79813300

C	4.44464900	-4.48834700	-3.71280100

H	4.99452500	-4.88687300	-4.57203900

H	5.12586900	-3.83547100	-3.15883400

H	3.62374200	-3.87267100	-4.09641300

C	0.79070500	3.37915700	3.47987100

H	1.64201600	3.04494300	4.08454700

H	0.75901300	4.47116800	3.49983400

C	4.22813300	7.62773600	-0.61408400

H	3.21480200	8.03924600	-0.59233400

H	4.84284900	8.29417600	-1.22822600

H	4.62094700	7.65024100	0.40741100

C	5.72596400	5.72860300	-1.24681300

H	5.82084800	4.72142800	-1.66663300

H	6.29888800	6.40516200	-1.88783400

H	6.19850600	5.73726500	-0.25924100

C	-2.40675100	5.38309200	-4.00351000

H	-2.38694400	5.45461900	-5.09623600

H	-1.56988900	5.97192000	-3.61614000

H	-3.33367300	5.84601200	-3.65014300

C	-2.23213400	-2.72691500	-3.94863300

C	3.69944100	6.20501700	-2.63137300

H	3.72088900	5.20257100	-3.07209500

H	4.30185400	6.86443500	-3.26491700

H	2.66719300	6.56565300	-2.66729500

C	5.09760100	-6.50624200	-2.34565100

H	4.74945300	-7.33676500	-1.72340600

H	5.81043900	-5.92261500	-1.75570600

H	5.63940500	-6.92640600	-3.19974700

C	3.00368300	-6.52940600	-3.71203700

H	2.62478700	-7.39752000	-3.16320200

H	3.57432800	-6.90657000	-4.56601300

H	2.14811200	-5.97287000	-4.10918300

C	8.21781200	0.82396700	-0.62932400

H	7.96679800	1.79304400	-0.18747400

H	8.54965300	0.16447400	0.17882400

H	9.06115600	0.97607800	-1.31165700

C	7.47503700	-1.10880100	-2.03357700

H	7.86787200	-1.81356400	-1.29390600

H	8.27847200	-0.90743300	-2.74856600

H	6.66253800	-1.59988600	-2.58008500

C	-3.51845500	-3.28909700	-4.60063500

H	-3.54040000	-3.04761400	-5.66877500

H	-4.42065200	-2.86914200	-4.14558400

H	-3.56719300	-4.37793000	-4.49843400

C	-2.20187500	-1.19017400	-4.12424600

H	-1.29608100	-0.76281900	-3.68226700

H	-3.06795000	-0.70804000	-3.66052200

H	-2.21292400	-0.93253700	-5.18861500

C	6.58908400	1.17308200	-2.51894700

H	7.42782500	1.34617700	-3.20136200

H	6.27080200	2.14735500	-2.13672800

H	5.76311000	0.75591700	-3.10328600

C	-1.01715500	-3.31524600	-4.69061800

H	-0.07189900	-2.94207200	-4.28304800

H	-1.05740900	-3.02842300	-5.74579400

H	-1.00368300	-4.40920000	-4.65051400

H	-0.45274200	-6.25016400	-0.34856600

C	-7.86829000	0.07892200	-2.16337400

C	-9.04195200	-0.72580500	-1.55549100

C	-8.38606900	1.48186200	-2.53220500

C	-7.38828500	-0.61483600	-3.46052700

H	-8.20340400	-0.65904700	-4.19035200

H	-6.55712700	-0.06608800	-3.91587000

H	-7.05518800	-1.64083900	-3.27698000

H	-9.87384500	-0.77641400	-2.26574200

H	-8.75168700	-1.75209200	-1.31206100

H	-9.40831300	-0.25433200	-0.63801400

H	-9.20449700	1.39295500	-3.25265200

H	-8.77509900	2.01707300	-1.65996700

H	-7.60761000	2.09722200	-2.99551200

H	-0.31360700	6.27615500	0.61970100

H	-0.14014300	2.98662200	3.89075100

H	-4.21189900	5.01452300	2.45793500

H	-3.22335600	-5.50634600	2.94215800

H	1.01323600	-5.44164700	2.77640800

C	3.66594600	-0.59076300	3.95159000

H	4.35738900	-1.44052800	3.96797100

H	4.22439300	0.31966600	4.20036300

H	2.87955100	-0.75049400	4.69112000

C	-4.15047300	0.13331800	3.06907400

H	-4.67399200	-0.80944500	3.25719200

H	-3.30261200	0.22021200	3.74869500

H	-4.84267900	0.96433500	3.23380400

C	-4.45374400	-2.40068800	0.64092100

H	-5.25457700	-3.15127400	0.66828600

H	-4.06493200	-2.34158100	1.65850100

C	-4.66421500	2.69252800	0.83901700

H	-5.48886400	3.41453600	0.78687300

H	-4.39122500	2.60803200	1.89081900

C	-0.17194800	-0.79397500	1.78589200

N	-0.78481600	0.46001800	1.19004500

C	-0.91071600	0.44338900	-0.33229300

H	-1.75715200	0.55302600	1.53772300

C	0.86105400	-0.95671800	-1.45603700

C	2.02414300	-1.07896800	-2.21456100

C	2.72038500	0.06310500	-2.61081600

C	2.26191200	1.32605600	-2.23492400

C	1.10040600	1.44905900	-1.47369700

C	0.38839700	0.30818500	-1.07952700

H	0.30988700	-1.84808300	-1.17630800

H	2.38146700	-2.06493000	-2.49211000

H	2.80296400	2.21687400	-2.53709100

H	0.73503600	2.43377000	-1.20180800

C	-0.11267500	-0.09167000	5.53347600

C	0.14740200	-0.00947300	4.16850200

C	-0.45121600	-0.90107700	3.26636700

C	-1.29691700	-1.90725300	3.75515800

C	-1.55515200	-2.01078000	5.12201800

C	-0.96707700	-1.09383800	5.98791700

H	0.33623100	0.58843700	6.24663900

H	0.84250000	0.73238100	3.79577900

H	-1.74053400	-2.62412600	3.07025800

H	-2.20131500	-2.78151100	5.52344400

H	-1.42076200	1.37534400	-0.58197500

H	-1.58332000	-0.38889900	-0.55106400

H	0.89948200	-0.76116900	1.59220600

H	-0.59933000	-1.64132000	1.25176000

H	-0.24845300	1.29453900	1.48660500

H	3.61623400	-0.03191200	-3.21522300

N	-1.24833200	-1.19025000	7.43365100

O	-2.00219100	-2.08680900	7.80526800

O	-0.71223000	-0.36492100	8.17031200

1 30 1.0 59 1.0

2 7 1.0 90 1.0

3 43 1.0 165 1.0

4 18 1.0 79 1.0

5 16 1.0 69 1.0

6 32 1.0 161 1.0

7 10 1.5 17 1.5

8 9 1.0 13 1.5 64 1.5

9

10 14 1.5 26 1.0

11 12 1.0 29 1.5 31 1.5

12

13 16 1.5 40 1.0

14 15 1.0 42 1.5

15

16 38 1.5

17 20 1.5 48 1.0

18 35 1.5 44 1.5

19 36 1.5 43 1.5 172 1.0

20 21 1.0 42 1.5

21

22 26 1.0 32 1.5 67 1.5

23 30 1.5 33 1.5 172 1.0

24 25 1.0 35 1.5 39 1.5

25

26 27 1.0 28 1.0

27

28

29 30 1.5 48 1.0

30

31 33 1.5 72 1.0

32 45 1.5

33 34 1.0

34

35 40 1.0

36 37 1.0 66 1.5

37

38 46 1.5 51 1.0

39 54 1.5 105 1.0

40 41 1.0 142 1.0

41

42 56 1.0

43 50 1.5

44 54 1.5 169 1.0

45 51 1.0 62 1.5

46 47 1.0 64 1.5

47

48 49 1.0 156 1.0

49

50 57 1.5 169 1.0

51 52 1.0 53 1.0

52

53

54 55 1.0

55

56 93 1.0 97 1.0 106 1.0

57 58 1.0 66 1.5

58

59 60 1.0 61 1.0 158 1.0

60

61

62 63 1.0 65 1.5

63

64 74 1.0

65 67 1.5 73 1.0

66 143 1.0

67 68 1.0

68

69 70 1.0 71 1.0 160 1.0

70

71

72 75 1.0 82 1.0 101 1.0

73 118 1.0 122 1.0 134 1.0

74 86 1.0 110 1.0 114 1.0

75 76 1.0 77 1.0 78 1.0

76

77

78

79 80 1.0 81 1.0 159 1.0

80

81

82 83 1.0 84 1.0 85 1.0

83

84

85

86 87 1.0 88 1.0 89 1.0

87

88

89

90 91 1.0 92 1.0 157 1.0

91

92

93 94 1.0 95 1.0 96 1.0

94

95

96

97 98 1.0 99 1.0 100 1.0

98

99

100

101 102 1.0 103 1.0 104 1.0

102

103

104

105 126 1.0 130 1.0 138 1.0

106 107 1.0 108 1.0 109 1.0

107

108

109

110 111 1.0 112 1.0 113 1.0

111

112

113

114 115 1.0 116 1.0 117 1.0

115

116

117

118 119 1.0 120 1.0 121 1.0

119

120

121

122 123 1.0 124 1.0 125 1.0

123

124

125

126 127 1.0 128 1.0 129 1.0

127

128

129

130 131 1.0 132 1.0 133 1.0

131

132

133

134 135 1.0 136 1.0 137 1.0

135

136

137

138 139 1.0 140 1.0 141 1.0

139

140

141

142

143 144 1.0 145 1.0 146 1.0

144 150 1.0 151 1.0 152 1.0

145 153 1.0 154 1.0 155 1.0

146 147 1.0 148 1.0 149 1.0

147

148

149

150

151

152

153

154

155

156

157

158

159

160

161 162 1.0 163 1.0 164 1.0

162

163

164

165 166 1.0 167 1.0 168 1.0

166

167

168

169 170 1.0 171 1.0

170

171

172 173 1.0 174 1.0

173

174

175 176 1.0 191 1.0 201 1.0 202 1.0

176 177 1.0 178 1.0 203 1.0

177 184 1.0 199 1.0 200 1.0

178

179 180 1.5 184 1.5 185 1.0

180 181 1.5 186 1.0

181 182 1.5 204 1.0

182 183 1.5 187 1.0

183 184 1.5 188 1.0

184

185

186

187

188

189 190 1.5 194 1.5 195 1.0

190 191 1.5 196 1.0

191 192 1.5

192 193 1.5 197 1.0

193 194 1.5 198 1.0

194 205 1.0

195

196

197

198

199

200

201

202

203

204

205 206 1.5 207 1.5

206

207

___________________________

**0 imaginary frequency**

**Energy: -4055.1902 Hartree**

(*endo-p-nitro-benzyl* )-**3f^+^**⊂**1a**

**Figure S54.** DFT-optimized structure of the (*endo-p-nitro-benzyl*)-**3f^+^**⊂**1a** complex.

O	3.03682000	-3.31249800	2.49695100

O	-0.74054700	-2.74140100	2.55640400

O	4.13571800	0.45173200	2.04963500

O	2.43117700	4.33728100	1.99685900

O	-1.49197100	3.47245700	2.43289300

O	-2.81939300	0.27901600	2.81049400

C	-1.34437800	-3.50153000	1.54449700

C	-2.03239700	5.13578000	-0.80604900

H	-1.48543700	5.79578300	-1.47313400

C	-2.70393700	-3.31339100	1.25259800

C	1.96725300	-3.98294800	-0.96072600

H	1.16033800	-4.49900400	-1.47148800

C	-1.38619600	4.69882100	0.35275700

C	-3.26718400	-4.08860200	0.22820200

H	-4.32018000	-3.96055700	0.01334700

C	-2.10881500	3.88963500	1.25159700

C	-0.56514400	-4.45051300	0.85736800

C	2.32023500	3.97584500	0.65950300

C	5.64893400	-0.69879400	0.56152100

C	-1.18626500	-5.20623700	-0.13996300

H	-0.59843000	-5.97352900	-0.63536500

C	-4.25659700	-1.18483300	1.52486100

C	4.04441200	-2.69019500	0.39046700

C	1.03233100	3.85929200	-1.37126000

H	0.12056600	4.09102600	-1.91062100

C	-3.57378300	-2.41029100	2.12607800

H	-4.36489600	-3.04277600	2.54895600

H	-2.96314100	-2.07221000	2.95794500

C	1.95743400	-3.97086100	0.43682300

C	3.01856500	-3.32932200	1.10418100

C	2.98484400	-3.37721000	-1.71427100

C	-3.89629900	0.11037700	1.92689200

C	3.99940900	-2.72180100	-1.00905600

H	4.79334900	-2.22170100	-1.54900000

C	1.13585200	4.27591700	-0.04101100

C	6.63168100	-0.62018700	-0.42923500

H	7.11199700	-1.54331200	-0.74197600

C	-3.42764600	3.49849000	0.97569500

C	2.06342000	3.17113300	-2.02877300

C	0.04713500	5.11022500	0.63373500

H	0.23906900	5.09103700	1.70539000

C	-2.53713300	-5.04345800	-0.48659500

C	5.05570200	0.49468300	1.00233000

C	3.36855100	3.27236600	0.04527100

C	-4.59973600	1.24555500	1.48873300

C	-4.01854800	3.94839300	-0.21502500

H	-5.04088100	3.65300000	-0.41490700

C	0.88597200	-4.71443400	1.23039100

H	1.04211700	-4.48425900	2.28248500

C	5.37808000	1.72476000	0.41740300

C	-4.23028900	2.64931800	1.95742700

H	-3.66751900	2.58046300	2.88528900

H	-5.16180800	3.18201600	2.18174500

C	3.21594000	2.88158800	-1.29054800

H	4.03091300	2.33942600	-1.75256400

C	-3.17370800	-5.95452400	-1.55467500

C	6.36589800	1.75012900	-0.57761000

H	6.62201900	2.71287500	-1.00606800

C	3.76785500	-4.39238500	3.08929200

H	3.70156500	-4.25993400	4.17127600

H	3.33652300	-5.36249900	2.81576900

C	-5.69673800	1.04601000	0.64720200

H	-6.27730800	1.91383700	0.35105900

C	-3.35245600	4.77711400	-1.12176000

C	-6.09995500	-0.22541100	0.21201400

C	7.02544800	0.59498000	-1.01127400

C	-5.35602000	-1.31993000	0.66502400

H	-5.66181800	-2.31982900	0.38427900

C	-1.67649000	4.39101500	3.51874200

H	-1.31948900	5.39361800	3.25937800

H	-1.09313900	4.01269500	4.36068300

C	2.98365900	-3.49545000	-3.25113200

C	-7.36277600	-0.37712900	-0.65843900

C	-4.00994500	5.31178400	-2.40801200

C	1.65027600	-2.96896800	-3.82903200

H	0.79179900	-3.53995300	-3.46332400

H	1.65299000	-3.05003000	-4.92103100

H	1.49347600	-1.91688000	-3.56941400

C	3.03661500	5.61392300	2.22667800

H	4.05827300	5.65106100	1.83171600

H	2.45208100	6.42214700	1.77108700

C	3.14933900	-4.98565500	-3.63656700

H	4.09130100	-5.38835100	-3.25082400

H	3.15213700	-5.09970500	-4.72593400

H	2.33554100	-5.59987900	-3.23931300

C	-3.18972700	4.85320300	-3.63715700

H	-3.64553200	5.22912400	-4.55905500

H	-3.15296600	3.76052400	-3.70197500

H	-2.16084300	5.22436900	-3.60402800

C	-0.82615400	-3.34596400	3.85973500

H	-1.87027000	-3.48173200	4.15992800

H	-0.32867800	-4.32085100	3.87231400

C	-2.35968500	-5.88919600	-2.86842900

H	-2.37848500	-4.88092000	-3.29278400

H	-2.79264200	-6.56864600	-3.60991800

H	-1.31669300	-6.18752400	-2.72468100

C	-3.17497900	-7.40988000	-1.02662900

H	-2.16146700	-7.77056300	-0.82575400

H	-3.62691200	-8.08036000	-1.76523300

H	-3.74969400	-7.49145800	-0.09835100

C	4.13186400	-2.69974900	-3.90008400

H	4.08096800	-2.80296100	-4.98817700

H	5.11377000	-3.06532900	-3.58359700

H	4.07259400	-1.63155700	-3.66587500

C	1.91437100	2.80005800	-3.51743700

C	-4.62484700	-5.55096700	-1.87825800

H	-4.68404800	-4.52539500	-2.25623400

H	-5.02243600	-6.21015000	-2.65562600

H	-5.28232800	-5.64502900	-1.00769600

C	-5.45531500	4.80807200	-2.58028900

H	-6.10173700	5.13238400	-1.75819100

H	-5.50467400	3.71615000	-2.64896600

H	-5.87663100	5.21197700	-3.50555700

C	-4.04026900	6.85838000	-2.36123600

H	-4.62040700	7.21420200	-1.50383600

H	-4.50232700	7.25541800	-3.27118200

H	-3.03600900	7.28580600	-2.28673800

C	-7.56031300	-1.82223000	-1.15467200

H	-7.71512000	-2.52490000	-0.32937500

H	-6.70941700	-2.16734800	-1.75116000

H	-8.44832500	-1.87193800	-1.79155600

C	-8.59692800	0.02270400	0.18654500

H	-8.69605100	-0.61881400	1.06823300

H	-9.51134100	-0.07451500	-0.40826800

H	-8.53277600	1.05916900	0.53205200

C	1.76290300	4.09752400	-4.34789800

H	1.65991100	3.85906900	-5.41199400

H	2.63765200	4.74463200	-4.22815700

H	0.87994700	4.67041200	-4.04895700

C	3.13546500	2.02857900	-4.05299200

H	3.29243000	1.08831100	-3.51360000

H	4.05466400	2.61909900	-3.98501400

H	2.98097600	1.78287400	-5.10804900

C	-7.27211300	0.54298700	-1.89818500

H	-8.17551100	0.43726100	-2.50774100

H	-6.41260200	0.28236600	-2.52254800

H	-7.18972800	1.59894800	-1.62307300

C	0.66186000	1.91746200	-3.72232300

H	0.74391200	0.98052200	-3.16151400

H	0.54425000	1.66746200	-4.78219900

H	-0.25355400	2.42099500	-3.39888800

H	0.16815000	6.15235200	0.30973300

C	8.14052100	0.61585800	-2.07356700

C	7.73096800	-0.27055800	-3.27354500

C	8.41993500	2.03606400	-2.60023300

C	9.44682100	0.06538800	-1.45282000

H	10.25115200	0.06838900	-2.19616600

H	9.76716400	0.67877900	-0.60457200

H	9.32831500	-0.96224100	-1.09671600

H	8.51707400	-0.26400000	-4.03605200

H	7.56854200	-1.31115000	-2.97713000

H	6.80829600	0.09551400	-3.73595200

H	9.21738600	1.99997700	-3.34849900

H	7.53924800	2.47505500	-3.08076700

H	8.74895600	2.71052500	-1.80309800

H	1.06398200	-5.79093300	1.11286500

H	-0.32621800	-2.67262000	4.55492400

H	4.82128900	-4.37587900	2.78771700

H	3.06471000	5.75498100	3.30904500

H	-2.73177800	4.45840900	3.80815300

C	-3.20048100	0.27232200	4.19715100

H	-3.86475900	1.11188500	4.42639100

H	-3.71128000	-0.66057100	4.45642000

H	-2.28243500	0.36017700	4.77907500

C	4.75822300	0.62423200	3.33169900

H	5.48105000	-0.17570700	3.52988900

H	5.27647400	1.58758700	3.39506200

H	3.96485500	0.58808200	4.07936900

C	4.65808600	2.99626800	0.81671400

H	5.34274600	3.84093800	0.66422500

H	4.41333200	2.98599100	1.87974000

C	5.21598900	-2.03846200	1.12446600

H	6.07282800	-2.72333500	1.08561800

H	4.93975700	-1.93465300	2.17354300

C	0.93522700	0.83872600	2.85113700

N	-0.05790700	0.13431500	1.93590300

C	0.26805800	0.45556700	0.48654300

H	-0.05223800	-0.88938400	2.09799800

C	-0.63234800	-1.29556100	-1.09036900

C	-1.55223500	-1.72851400	-2.04075200

C	-2.58812200	-0.87278100	-2.40358200

C	-2.73076800	0.39704400	-1.85095500

C	-1.80734400	0.81306400	-0.89749200

C	-0.75747900	-0.02902400	-0.50549100

H	0.19146000	-1.94593400	-0.81577200

H	-1.47364700	-2.70503300	-2.49749500

H	-3.54580000	1.03528200	-2.16441200

H	-1.89922100	1.80450600	-0.46920200

C	0.29794100	0.65309700	6.60402300

C	0.26552600	1.07610400	5.27426600

C	0.91427200	0.34073300	4.27351600

C	1.61012700	-0.82640500	4.62660000

C	1.64255700	-1.24719500	5.95770500

C	0.98672600	-0.51119200	6.94726600

H	-0.20151800	1.23727800	7.37078000

H	-0.25266300	1.99321200	5.00931600

H	2.13204800	-1.40383500	3.86625100

H	2.19030200	-2.14613400	6.22420100

H	1.02229900	-0.83728400	7.98211200

H	1.24623100	0.00825200	0.29829600

H	0.37466700	1.54093600	0.43700500

H	0.68449500	1.89959600	2.79021700

H	1.91983600	0.68663900	2.40128800

H	-1.02752400	0.41996400	2.17728900

N	-3.56201300	-1.32286000	-3.41184700

O	-3.47582900	-2.48242600	-3.81622400

O	-4.40678000	-0.51346800	-3.79288100

1 30 1.0 59 1.0

2 7 1.0 90 1.0

3 43 1.0 165 1.0

4 18 1.0 79 1.0

5 16 1.0 69 1.0

6 32 1.0 161 1.0

7 10 1.5 17 1.5

8 9 1.0 13 1.5 64 1.5

9

10 14 1.5 26 1.0

11 12 1.0 29 1.5 31 1.5

12

13 16 1.5 40 1.0

14 15 1.0 42 1.5

15

16 38 1.5

17 20 1.5 48 1.0

18 35 1.5 44 1.5

19 36 1.5 43 1.5 172 1.0

20 21 1.0 42 1.5

21

22 26 1.0 32 1.5 67 1.5

23 30 1.5 33 1.5 172 1.0

24 25 1.0 35 1.5 39 1.5

25

26 27 1.0 28 1.0

27

28

29 30 1.5 48 1.0

30

31 33 1.5 72 1.0

32 45 1.5

33 34 1.0

34

35 40 1.0

36 37 1.0 66 1.5

37

38 46 1.5 51 1.0

39 54 1.5 105 1.0

40 41 1.0 142 1.0

41

42 56 1.0

43 50 1.5

44 54 1.5 169 1.0

45 51 1.0 62 1.5

46 47 1.0 64 1.5

47

48 49 1.0 156 1.0

49

50 57 1.5 169 1.0

51 52 1.0 53 1.0

52

53

54 55 1.0

55

56 93 1.0 97 1.0 106 1.0

57 58 1.0 66 1.5

58

59 60 1.0 61 1.0 158 1.0

60

61

62 63 1.0 65 1.5

63

64 74 1.0

65 67 1.5 73 1.0

66 143 1.0

67 68 1.0

68

69 70 1.0 71 1.0 160 1.0

70

71

72 75 1.0 82 1.0 101 1.0

73 118 1.0 122 1.0 134 1.0

74 86 1.0 110 1.0 114 1.0

75 76 1.0 77 1.0 78 1.0

76

77

78

79 80 1.0 81 1.0 159 1.0

80

81

82 83 1.0 84 1.0 85 1.0

83

84

85

86 87 1.0 88 1.0 89 1.0

87

88

89

90 91 1.0 92 1.0 157 1.0

91

92

93 94 1.0 95 1.0 96 1.0

94

95

96

97 98 1.0 99 1.0 100 1.0

98

99

100

101 102 1.0 103 1.0 104 1.0

102

103

104

105 126 1.0 130 1.0 138 1.0

106 107 1.0 108 1.0 109 1.0

107

108

109

110 111 1.0 112 1.0 113 1.0

111

112

113

114 115 1.0 116 1.0 117 1.0

115

116

117

118 119 1.0 120 1.0 121 1.0

119

120

121

122 123 1.0 124 1.0 125 1.0

123

124

125

126 127 1.0 128 1.0 129 1.0

127

128

129

130 131 1.0 132 1.0 133 1.0

131

132

133

134 135 1.0 136 1.0 137 1.0

135

136

137

138 139 1.0 140 1.0 141 1.0

139

140

141

142

143 144 1.0 145 1.0 146 1.0

144 150 1.0 151 1.0 152 1.0

145 153 1.0 154 1.0 155 1.0

146 147 1.0 148 1.0 149 1.0

147

148

149

150

151

152

153

154

155

156

157

158

159

160

161 162 1.0 163 1.0 164 1.0

162

163

164

165 166 1.0 167 1.0 168 1.0

166

167

168

169 170 1.0 171 1.0

170

171

172 173 1.0 174 1.0

173

174

175 176 1.0 191 1.0 202 1.0 203 1.0

176 177 1.0 178 1.0 204 1.0

177 184 1.0 200 1.0 201 1.0

178

179 180 1.5 184 1.5 185 1.0

180 181 1.5 186 1.0

181 182 1.5 205 1.0

182 183 1.5 187 1.0

183 184 1.5 188 1.0

184

185

186

187

188

189 190 1.5 194 1.5 195 1.0

190 191 1.5 196 1.0

191 192 1.5

192 193 1.5 197 1.0

193 194 1.5 198 1.0

194 199 1.0

195

196

197

198

199

200

201

202

203

204

205 206 1.5 207 1.5

206

207

___________________________

**0 imaginary frequency**

**Energy: -4055.1951 Hartree**

**DFT structure of 3a-f**⊂**2 complexes**

**3a^+^**⊂**2**

**Figure S55.** DFT-optimized structure of the **3a^+^**⊂**2** complex.

C	3.28293000	3.77327400	0.57984100

H	4.28559200	4.19324700	0.71354500

H	2.80645400	4.30578300	-0.24951500

C	3.34793100	2.26581900	0.31788400

H	2.70906200	3.98556300	1.48884300

H	3.90142100	1.77294500	1.12884800

C	1.96533200	1.61158300	0.19117700

H	3.92333500	2.07310600	-0.59760900

C	2.06807900	0.10489600	-0.08510500

H	1.39973300	1.78116500	1.11472000

H	1.40002200	2.09969900	-0.61175900

C	0.70600200	-0.57623000	-0.14247400

H	2.60085400	-0.05671100	-1.02719600

H	2.66854300	-0.35901500	0.70507400

N	0.81811700	-2.07646200	-0.29825100

H	0.14907900	-0.41365100	0.78323600

H	0.10498900	-0.21897100	-0.98213700

C	-0.52866600	-2.76382000	-0.29217700

C	-0.45490700	-4.26978300	-0.07179800

H	-1.10424900	-2.30573700	0.51390300

H	-1.01243200	-2.52531700	-1.24026100

C	0.28781200	-5.06958000	-1.14647100

H	-0.03517700	-4.47531500	0.92156300

H	-1.50022000	-4.58512000	-0.02736900

C	0.12224100	-6.58532600	-0.96940600

H	-0.07506600	-4.77243500	-2.14065700

H	1.35664200	-4.82258200	-1.12429100

C	0.87154800	-7.41058000	-2.01915800

H	0.46325800	-6.87228400	0.03473000

H	-0.94712200	-6.83444700	-1.00364700

H	0.71881100	-8.48319400	-1.86674200

H	0.53078400	-7.16868700	-3.03223900

H	1.95067900	-7.22323900	-1.97829300

H	1.32969700	-2.30944100	-1.16511800

H	1.41594200	-2.46332000	0.45586900

O	-3.24880500	-3.55278000	-1.81491400

O	-3.21029300	-3.97397600	1.52088500

O	0.05871200	-2.86140900	-4.67978400

O	2.82497400	-2.92326000	-2.42507600

O	2.87640300	-3.32166400	1.27329200

O	0.30671700	-3.94538700	4.04110300

C	-3.95226300	-2.91688900	2.03527000

C	5.97106200	-1.34008100	1.39578600

H	6.81530900	-1.11242300	0.75430700

C	-3.56757100	-2.35162900	3.26503000

C	-5.95942400	-1.07121100	-1.56657100

H	-6.80927000	-0.86819800	-0.92596100

C	4.95362700	-2.15342800	0.88313000

C	-4.37101700	-1.34629500	3.80877300

C	3.90332800	-2.50296900	1.75155800

C	-5.06213600	-2.43374800	1.32801300

C	3.74948400	-1.88958200	-2.59969600

C	-1.35429800	-1.04413500	-3.94065400

C	-5.82435400	-1.41186500	1.91237100

H	-6.69996900	-1.06532200	1.37668600

C	-1.06958800	-1.97070700	3.82429700

C	-3.77173200	-1.65222700	-3.20304100

C	5.70027200	-0.66520500	-1.92923200

H	6.53424000	-0.54534700	-1.25106100

C	-2.32421700	-2.82970600	3.99198300

H	-2.55476000	-2.88044900	5.06485300

H	-2.07217500	-3.84298600	3.68029300

C	-5.14187100	-2.16241700	-1.23941600

C	-4.05943200	-2.45372900	-2.08287300

C	-5.74094700	-0.27304900	-2.69363700

C	0.20598100	-2.56386100	3.91853800

C	-4.63319800	-0.59049700	-3.49421000

C	4.80729500	-1.72409200	-1.69534700

C	-1.45532800	0.28048800	-3.51506300

H	-2.42947800	0.63347500	-3.20494600

C	3.84993000	-2.03153300	3.07275300

C	5.56868600	0.21488200	-3.00664300

C	5.03105100	-2.70420600	-0.54030600

H	4.30861800	-3.51323100	-0.63340400

C	-5.51784300	-0.86144900	3.16036000

C	-0.08135200	-1.53334200	-4.29982500

C	3.56373100	-1.02224400	-3.69336600

C	1.38147100	-1.79324500	3.85159000

C	4.88392200	-1.19969300	3.51900700

H	4.84101700	-0.85199200	4.54488900

C	-5.46434700	-3.02346100	-0.02079300

H	-4.97847600	-3.99003600	-0.13555400

C	1.05472400	-0.70716400	-4.23955400

C	2.74838500	-2.44438100	4.03563700

H	2.61718400	-3.52657300	4.00889000

H	3.09826800	-2.20961200	5.04835900

C	4.48168200	0.01200100	-3.87305800

H	4.35342500	0.66287500	-4.73322900

C	-2.58070900	-1.94675800	-4.09644800

H	-2.25154900	-2.97606000	-3.95583400

H	-2.91556800	-1.87045800	-5.14017200

C	0.89471000	0.62312300	-3.84096300

H	1.77720700	1.25278000	-3.83325900

C	-3.69023300	-4.76049500	-2.44304200

H	-2.98143700	-5.54071000	-2.15760000

H	-4.69493400	-5.04286600	-2.10712100

C	1.26231900	-0.40840000	3.69585200

H	2.16518800	0.19203600	3.63630400

C	5.96414900	-0.84014400	2.70500500

C	0.01100500	0.21854400	3.60584000

C	-0.35284800	1.15385300	-3.47779400

C	-1.13810300	-0.58610900	3.65881600

H	-2.11207600	-0.11381100	3.60470300

C	2.93059900	-4.71382100	1.65153200

H	2.76784300	-5.31511300	0.75300400

H	2.14836400	-4.92964800	2.38402700

C	2.42253400	-1.22008500	-4.67978800

H	2.69672000	-0.70684900	-5.60918900

H	2.32139400	-2.27680200	-4.92616800

C	3.16441200	-4.12692000	-3.13714800

H	3.92741300	-4.69653800	-2.59388600

H	2.25307700	-4.71581200	-3.22845300

C	-3.66969200	-5.25997700	1.94815800

H	-4.69686900	-5.44984900	1.61429000

H	-3.00095700	-5.99769100	1.49926800

H	6.02685800	-3.14625100	-0.66998000

H	-6.54618800	-3.19878000	-0.00767700

H	-4.43677700	-0.00801000	-4.39013800

C	6.92401400	0.50984900	4.65522200

C	7.27232100	1.28105600	2.30399800

C	7.13145700	0.03205800	3.20548600

H	6.01630200	1.11304800	4.76097700

H	6.86594800	-0.32707100	5.35859700

H	6.36012300	1.88629600	2.32354300

H	7.47584400	1.01348600	1.26298900

C	8.44145500	-0.79013900	3.14994200

H	8.67358400	-1.12174700	2.13342900

H	8.37474300	-1.67912700	3.78524700

C	-7.38032600	0.51373300	-4.42776000

C	-6.67978100	0.88035300	-3.09694200

C	-7.76521800	1.14816100	-2.03715900

H	-6.65813600	0.35077000	-5.23354400

H	-7.97334100	-0.40011600	-4.32020600

C	-5.87054700	2.18399100	-3.29307600

H	-8.42548200	0.28612600	-1.89866100

H	-7.33044000	1.40861100	-1.06617100

H	-5.12124100	2.08642500	-4.08441700

H	-5.35427400	2.48160700	-2.37477600

C	7.66301200	1.45179800	-2.20884300

C	7.27432000	1.05892300	-4.64931000

C	5.84139500	2.70063100	-3.37677300

H	5.34708200	2.93986800	-2.42967700

C	6.57784100	1.34266500	-3.29622800

H	5.08250300	2.71129600	-4.16462000

H	7.23230200	1.66210500	-1.22421400

H	8.26485400	0.53992900	-2.13674000

H	7.81179300	0.10559000	-4.62242600

H	7.99721600	1.84944300	-4.87727400

C	-7.58939000	0.62966700	2.96915700

C	-6.93875100	-0.35296400	5.17470100

C	-5.54296900	1.47042700	4.13052800

H	-4.69086000	1.25383800	4.78124000

C	-6.39102200	0.20999000	3.84107800

H	-5.15473500	1.90226600	3.20186700

H	-7.26925400	1.05602200	2.01238500

H	-8.26006200	-0.21082000	2.76373000

H	-6.13354000	-0.62813300	5.86224600

H	-7.55063500	-1.24411300	5.00212300

H	-4.09721000	-0.94626200	4.78127000

H	-3.63429600	-5.35758900	3.03978400

H	3.54605500	-3.89248600	-4.13479000

H	3.91086800	-4.95561400	2.06974700

H	-3.70260600	-4.66470500	-3.53544200

C	-0.12954700	-3.11136500	-6.07795400

C	0.28720200	-4.43407200	5.38829200

H	0.36528500	-5.52115700	5.32569000

H	1.13165900	-4.04170200	5.96705300

H	-0.64535100	-4.16775800	5.89764200

H	0.01291500	-4.18390800	-6.22348400

H	-1.13760200	-2.83404100	-6.40543100

H	0.60219600	-2.56240000	-6.68248600

H	7.76976800	1.13338500	4.95996100

H	8.10137400	1.90649200	2.65152700

H	9.28379500	-0.18498100	3.50151900

H	8.34479200	2.27236100	-2.45155600

H	6.55775000	1.01582300	-5.47494700

H	6.55366200	3.50274100	-3.59714800

H	-8.05287200	1.32000600	-4.74018200

H	-8.39025500	1.98814700	-2.35503200

H	-6.53968300	3.00201900	-3.58073900

H	-8.17356100	1.39417200	3.49030800

H	-7.56250000	0.39504400	5.67588400

H	-6.15233300	2.23303300	4.62719600

C	0.59525100	3.41864800	-2.87099100

C	0.44386900	4.74144200	-2.45988200

C	-0.83156800	5.27473000	-2.19885700

C	-1.95075100	4.45969000	-2.44673900

C	-1.78668700	3.14391300	-2.87988400

C	-0.51425000	2.57966000	-3.06936800

H	1.61177300	3.07445700	-3.01637400

H	-2.69482300	2.58184800	-3.05562200

C	-1.20837700	3.63629900	2.51393400

C	-1.09795000	2.25209400	2.64026900

C	-0.10886900	1.69486900	3.47352200

C	0.75803600	2.54465400	4.16088300

C	0.64542900	3.93974800	4.05226100

C	-0.35055300	4.49737300	3.23616900

H	-1.75395200	1.59975500	2.07888600

H	1.51728100	2.15527200	4.82951200

O	1.50863300	4.65841500	4.82514200

O	-0.47948000	5.86157400	3.10361800

O	-2.11790500	4.26683000	1.71711400

O	1.58363800	5.47824500	-2.23198700

O	-1.00210500	6.55612700	-1.74184200

O	-3.23156800	4.88446000	-2.18703900

C	-0.57337000	6.77831000	-0.38021000

H	-1.10440100	6.11683400	0.30911900

H	0.50461500	6.62384000	-0.28298200

H	-0.81856000	7.81841700	-0.15976600

C	1.75878200	6.62441700	-3.07948200

H	0.94001300	7.33829200	-2.95742700

H	2.70067900	7.08342300	-2.77524000

H	1.82630300	6.31773100	-4.13004200

C	-3.69250400	6.02372400	-2.93001300

H	-3.09701800	6.91102600	-2.70619900

H	-3.66285800	5.81717600	-4.00659400

H	-4.72753300	6.18203200	-2.62262300

C	-2.98197400	3.47008700	0.91201400

H	-2.41101900	2.80257500	0.25694100

H	-3.54443700	4.16808400	0.29308800

H	-3.66566900	2.87614300	1.53092300

C	-1.58582800	6.42670800	3.82227100

H	-1.49681400	6.22708600	4.89667800

H	-1.54397800	7.50363700	3.64831200

H	-2.53652000	6.03356300	3.45061600

C	2.04627900	5.89893200	4.35380100

H	2.29423500	5.84566200	3.28915900

H	1.35824900	6.73099500	4.51483100

H	2.95829000	6.05722400	4.93351100

1 2 1.0 3 1.0 4 1.0 5 1.0

2

3

4 6 1.0 7 1.0 8 1.0

5

6

7 9 1.0 10 1.0 11 1.0

8

9 12 1.0 13 1.0 14 1.0

10

11

12 15 1.0 16 1.0 17 1.0

13

14

15 18 1.0 34 1.0 35 1.0

16

17

18 19 1.0 20 1.0 21 1.0

19 22 1.0 23 1.0 24 1.0

20

21

22 25 1.0 26 1.0 27 1.0

23

24

25 28 1.0 29 1.0 30 1.0

26

27

28 31 1.0 32 1.0 33 1.0

29

30

31

32

33

34

35

36 64 1.0 94 1.0

37 42 1.0 113 1.0

38 76 1.0 164 1.0

39 52 1.0 110 1.0

40 50 1.0 104 1.0

41 66 1.0 165 1.0

42 45 1.5 51 1.5

43 44 1.0 48 1.5 99 1.5

44

45 49 1.5 60 1.0

46 47 1.0 63 1.5 65 1.5

47

48 50 1.5 73 1.0

49 75 1.5 159 1.0

50 71 1.5

51 54 1.5 81 1.0

52 68 1.5 77 1.5

53 69 1.5 76 1.5 89 1.0

54 55 1.0 75 1.5

55

56 60 1.0 66 1.5 102 1.5

57 64 1.5 67 1.5 89 1.0

58 59 1.0 68 1.5 72 1.5

59

60 61 1.0 62 1.0

61

62

63 64 1.5 81 1.0

64

65 67 1.5 130 1.0

66 78 1.5

67 118 1.0

68 73 1.0

69 70 1.0 101 1.5

70

71 79 1.5 84 1.0

72 87 1.5 143 1.0

73 74 1.0 116 1.0

74

75 153 1.0

76 83 1.5

77 87 1.5 107 1.0

78 84 1.0 97 1.5

79 80 1.0 99 1.5

80

81 82 1.0 117 1.0

82

83 92 1.5 107 1.0

84 85 1.0 86 1.0

85

86

87 88 1.0

88

89 90 1.0 91 1.0

90

91

92 93 1.0 101 1.5

93

94 95 1.0 96 1.0 163 1.0

95

96

97 98 1.0 100 1.5

98

99 121 1.0

100 102 1.5 194 1.0

101 189 1.0

102 103 1.0

103

104 105 1.0 106 1.0 162 1.0

105

106

107 108 1.0 109 1.0

108

109

110 111 1.0 112 1.0 161 1.0

111

112

113 114 1.0 115 1.0 160 1.0

114

115

116

117

118

119 121 1.0 122 1.0 123 1.0 172 1.0

120 121 1.0 124 1.0 125 1.0 173 1.0

121 126 1.0

122

123

124

125

126 127 1.0 128 1.0 174 1.0

127

128

129 130 1.0 132 1.0 133 1.0 178 1.0

130 131 1.0 134 1.0

131 135 1.0 136 1.0 179 1.0

132

133

134 137 1.0 138 1.0 180 1.0

135

136

137

138

139 143 1.0 145 1.0 146 1.0 175 1.0

140 143 1.0 147 1.0 148 1.0 176 1.0

141 142 1.0 143 1.0 144 1.0 177 1.0

142

143

144

145

146

147

148

149 153 1.0 155 1.0 156 1.0 181 1.0

150 153 1.0 157 1.0 158 1.0 182 1.0

151 152 1.0 153 1.0 154 1.0 183 1.0

152

153

154

155

156

157

158

159

160

161

162

163

164 169 1.0 170 1.0 171 1.0

165 166 1.0 167 1.0 168 1.0

166

167

168

169

170

171

172

173

174

175

176

177

178

179

180

181

182

183

184 185 1.5 189 1.5 190 1.0

185 186 1.5 203 1.0

186 187 1.5 204 1.0

187 188 1.5 205 1.0

188 189 1.5 191 1.0

189

190

191

192 193 1.5 197 1.5 202 1.0

193 194 1.5 198 1.0

194 195 1.5

195 196 1.5 199 1.0

196 197 1.5 200 1.0

197 201 1.0

198

199

200 226 1.0

201 222 1.0

202 218 1.0

203 210 1.0

204 206 1.0

205 214 1.0

206 207 1.0 208 1.0 209 1.0

207

208

209

210 211 1.0 212 1.0 213 1.0

211

212

213

214 215 1.0 216 1.0 217 1.0

215

216

217

218 219 1.0 220 1.0 221 1.0

219

220

221

222 223 1.0 224 1.0 225 1.0

223

224

225

226 227 1.0 228 1.0 229 1.0

227

228

229

___________________________

**0 imaginary frequency**

**Energy: -4537.8394 Hartree**

**3b^+^**⊂**2**

**Figure S56.** DFT-optimized structure of the **3b^+^**⊂**2** complex.

O	-1.12394800	2.64572700	3.08949100

O	2.11994300	3.70662200	3.36655100

O	-3.43136300	-0.84101100	3.48254300

O	-1.46868100	-3.75999100	2.48512200

O	2.26464800	-4.26345200	2.45277600

O	3.41914400	-0.02299000	2.79186300

C	2.15511200	3.79012400	1.97854700

C	1.27291800	-4.05658300	-1.08469000

H	0.50647300	-4.46483600	-1.73545600

C	3.22710700	3.21598500	1.27562500

C	-2.29647500	5.45946100	1.02702800

H	-1.88530800	6.37639300	0.61443000

C	1.24326300	-4.41706500	0.26585100

C	3.22951400	3.29862500	-0.12336000

C	2.24955900	-3.90900500	1.10696900

C	1.10068000	4.42367100	1.29659600

C	-2.03933400	-4.26505200	1.32278200

C	-4.37590500	0.72611100	1.90894500

C	1.15450900	4.48827400	-0.09826700

H	0.34578600	4.99979600	-0.61275000

C	4.93195300	1.29637300	1.44139100

C	-3.32403000	3.11384200	2.15725800

C	-1.84228600	-5.57145500	-0.69411700

H	-1.23475600	-6.20800900	-1.32790600

C	4.38938400	2.58111800	2.03642500

H	5.21145900	3.30672800	2.08128500

H	4.06224000	2.41521200	3.06253800

C	-1.44772300	4.64678100	1.78026800

C	-1.97586800	3.46180200	2.32886200

C	-3.65495700	5.16437300	0.83464900

C	4.41865600	0.04174500	1.80957500

C	-4.13955600	3.99842800	1.43064100

C	-1.25377400	-5.05597700	0.46852400

C	-5.10836500	0.93361000	0.74005100

H	-5.34026300	1.94393400	0.43101600

C	3.22301100	-3.01686900	0.62547400

C	-3.17796000	-5.32529400	-1.03071100

C	0.19290300	-5.38592700	0.80404300

H	0.31663100	-5.44247000	1.88440100

C	2.21429000	3.94394200	-0.83991400

C	-4.12823300	-0.60489400	2.30060500

C	-3.38136900	-3.97188500	1.01600000

C	4.89558700	-1.14704900	1.23383000

C	3.19908300	-2.67797900	-0.73329400

H	3.95428100	-1.99484600	-1.09962300

C	-0.02420600	5.10757200	2.06035700

H	0.18626000	5.01245200	3.12526500

C	-4.54372600	-1.69888200	1.51901200

C	4.30561800	-2.50067100	1.57579300

H	3.88873500	-2.49149000	2.58334600

H	5.12608500	-3.23046600	1.59269800

C	-3.91970200	-4.52305300	-0.15136500

H	-4.96846700	-4.34195600	-0.36443100

C	-3.92826500	1.87924300	2.81199600

H	-3.21802100	1.47322700	3.52955900

H	-4.80128300	2.21021100	3.39184200

C	-5.28063600	-1.43271800	0.36230900

H	-5.60965200	-2.26089600	-0.25536600

C	-1.06792900	3.01339000	4.47915700

H	-1.98680100	2.71560800	4.99731000

H	-0.21229200	2.49595000	4.91303700

C	5.94217000	-1.05476400	0.30932400

H	6.31849600	-1.96850000	-0.14189300

C	2.24962500	-3.20426100	-1.61925800

C	6.50587100	0.17277900	-0.06514300

C	-5.58872900	-0.12734900	-0.03974100

C	5.97539400	1.33600600	0.51212600

H	6.41497500	2.29682200	0.26058600

C	3.03544200	-5.43277300	2.75106600

H	4.08916600	-5.29896200	2.48018700

H	2.64339300	-6.31113200	2.22565500

C	-4.24666500	-3.13411200	1.95330900

H	-5.20387200	-3.65514700	2.08044500

H	-3.76693600	-3.09490600	2.92823000

C	-1.62405200	-4.60200800	3.63291800

H	-2.68088200	-4.73186600	3.89486200

H	-1.18505400	-5.59272300	3.46588100

C	2.80780900	4.76640700	4.04257500

H	3.87371500	4.78124700	3.78800000

H	2.37391100	5.74313300	3.79778900

H	0.41100900	-6.38445800	0.40467700

H	0.01845100	6.17888700	1.83290200

H	-5.19585800	3.77076700	1.36375700

C	3.35461900	-1.87979600	-3.51113800

C	0.91334500	-2.39235300	-3.61728100

C	2.28315400	-2.92061600	-3.13419900

H	3.18269200	-0.91929300	-3.01358500

H	4.36507200	-2.21832200	-3.26153600

H	0.64947500	-1.45602600	-3.11722700

H	0.10642200	-3.10698400	-3.43098900

C	2.60800900	-4.24097500	-3.87476100

H	1.85421600	-5.00960400	-3.67887800

H	3.57959300	-4.63813800	-3.56356000

C	-4.54460700	7.51907300	0.70343400

C	-4.54587300	6.12712400	0.02729000

C	-3.98747500	6.25494200	-1.41012100

H	-4.93771700	7.46042900	1.72350000

H	-3.53997100	7.94895000	0.75591200

C	-6.00401500	5.63959800	-0.06708300

H	-2.96059300	6.63279600	-1.41853600

H	-3.99623300	5.28724000	-1.92254100

H	-6.47409800	5.56016500	0.91867600

H	-6.08256500	4.66917500	-0.56815200

C	-2.87533900	-6.76542800	-3.12480500

C	-5.03014500	-6.82051400	-1.85904900

C	-4.37848300	-4.76975500	-3.17892600

H	-5.10779700	-4.14435800	-2.65547100

C	-3.84883800	-5.91647000	-2.28553300

H	-3.56072500	-4.12212700	-3.51281200

H	-2.02196300	-6.17791800	-3.47962600

H	-2.49234900	-7.62387700	-2.56360000

H	-4.68469500	-7.64610600	-1.22869500

H	-5.78749600	-6.26590600	-1.29703300

C	2.39759600	5.62393000	-2.69574700

C	3.44655500	3.37321500	-3.00932000

C	0.95712200	3.57828100	-3.00954400

H	0.82217400	2.51272200	-2.80049900

C	2.25786800	4.11667500	-2.37086200

H	0.07067400	4.10500300	-2.64412300

H	1.55527800	6.20215300	-2.30403700

H	3.31585100	6.03578700	-2.26501700

H	4.40883900	3.75576800	-2.65404500

H	3.40926900	2.29667800	-2.81117200

H	4.06035600	2.85061000	-0.65372400

H	2.69583000	4.57842100	5.11233300

H	-1.10324300	-4.10709800	4.45375600

H	2.95713400	-5.59218300	3.82836300

H	-0.93485800	4.09173400	4.59601200

C	-4.26797700	-0.98122700	4.63621900

C	3.95129100	-0.12181300	4.12716400

H	4.57579700	0.74533600	4.36399000

H	3.09997400	-0.16438500	4.80655000

H	4.55014600	-1.03181700	4.23656900

H	-3.60372000	-1.17723900	5.48010900

H	-4.84290600	-0.06686100	4.82629800

H	-4.96672900	-1.81796400	4.52143500

C	0.82298400	0.26207000	0.70732900

N	0.57561500	0.26233200	2.21268300

C	-0.10697600	-0.98736400	2.74820400

H	1.51780900	0.33513300	2.63393800

C	-0.67129600	-0.39251900	5.14416200

C	-0.48066200	-0.53300100	6.51895000

C	0.46373800	-1.43978900	7.00540600

C	1.20883400	-2.20830400	6.10997000

C	1.02710300	-2.06008700	4.73223100

C	0.09051500	-1.14156600	4.23702400

H	-1.43519700	0.27555500	4.76491500

H	-1.07697000	0.05436800	7.21109100

H	0.60715200	-1.55590200	8.07537900

H	1.93004900	-2.92950600	6.48319000

H	1.59068200	-2.67921600	4.03962900

C	-2.08982600	1.37785500	-1.46356200

C	-1.00381300	1.41098900	-0.59038700

C	-0.41295800	0.21915500	-0.14964100

C	-0.93486100	-1.00527400	-0.58860300

C	-2.02661700	-1.03655700	-1.45510800

C	-2.59915400	0.15478600	-1.90180000

H	-2.53112800	2.30848500	-1.80683200

H	-0.59839600	2.36440500	-0.26761900

H	-0.47385800	-1.93631600	-0.27506600

H	-2.42308300	-1.99177900	-1.78309100

H	-3.44524700	0.13149300	-2.58066400

H	0.32370100	-1.83335400	2.21429100

H	-1.16176700	-0.91586500	2.48822500

H	1.46381400	-0.60313700	0.52447300

H	1.40346500	1.16701600	0.51760200

H	0.03172200	1.10249300	2.47987300

H	3.32860200	-1.70145500	-4.59040200

H	0.94302900	-2.20594100	-4.69613900

H	2.64096500	-4.07275800	-4.95668100

H	-3.39337700	-7.15548900	-4.00613800

H	-5.51886600	-7.24866400	-2.74084300

H	-4.86971200	-5.17674200	-4.06930200

H	-5.17311500	8.21501700	0.13786500

H	-4.60081800	6.94921500	-1.99401600

H	-6.59130700	6.35441700	-0.65111000

H	2.43267500	5.77769500	-3.77965300

H	3.42413300	3.50960400	-4.09465400

H	0.99047200	3.70934200	-4.09629000

C	-7.45118000	-0.72942400	-1.61378500

C	-8.22556400	-0.47626000	-2.74827400

C	-7.97143200	0.66100700	-3.54661000

C	-6.92022900	1.51706600	-3.19260100

C	-6.14339700	1.25151200	-2.06013600

C	-6.40258400	0.13701600	-1.25495900

H	-7.68117300	-1.57504600	-0.97837700

H	-5.33045500	1.93233400	-1.83536400

C	9.70176000	-0.65966100	-1.92872500

C	8.64364000	-0.72816800	-1.02004000

C	7.62739800	0.24441700	-1.03573400

C	7.68332300	1.27273300	-1.98071600

C	8.74212300	1.35015700	-2.89118200

C	9.76390000	0.39100200	-2.87095500

H	8.61955600	-1.50986000	-0.27143600

H	6.90324800	2.02269400	-2.04780700

O	8.76964800	2.41604000	-3.75185500

O	10.78507800	0.45222400	-3.78383100

O	10.73980000	-1.54092900	-1.97674100

O	-9.26974300	-1.24903900	-3.16037300

O	-8.71385200	0.90670800	-4.67389000

O	-6.65005000	2.66295100	-3.89665300

C	-10.01937500	1.44944100	-4.43318600

H	-9.95144200	2.40498300	-3.90037700

H	-10.46605900	1.61191600	-5.41560800

H	-10.63660200	0.74841600	-3.86349500

C	-9.58750000	-2.41222700	-2.41007500

H	-10.42761900	-2.87941000	-2.92487700

H	-8.74514900	-3.11475200	-2.38185800

H	-9.88470300	-2.16412200	-1.38341200

C	-6.25355100	2.49494700	-5.26431600

H	-6.06412200	3.49972300	-5.64656100

H	-5.33036500	1.90468600	-5.32911200

H	-7.03826900	2.01445600	-5.85220300

C	10.74450700	-2.63014100	-1.06542100

H	10.79159300	-2.28721300	-0.02409800

H	9.86097200	-3.26736900	-1.19607800

H	11.64068700	-3.20746500	-1.29472800

C	12.03142600	0.94013200	-3.26797500

H	12.41882000	0.27816600	-2.48772400

H	12.72404100	0.95276900	-4.11119500

H	11.91774300	1.95635700	-2.87339900

C	8.70291100	2.11036200	-5.15167000

H	8.71680800	3.07184100	-5.66803400

H	9.55406700	1.50450300	-5.46937600

H	7.76769100	1.58721500	-5.38802400

1 29 1.0 59 1.0

2 7 1.0 78 1.0

3 41 1.0 129 1.0

4 17 1.0 75 1.0

5 15 1.0 69 1.0

6 31 1.0 130 1.0

7 10 1.5 16 1.5

8 9 1.0 13 1.5 64 1.5

9

10 14 1.5 25 1.0

11 12 1.0 28 1.5 30 1.5

12

13 15 1.5 38 1.0

14 40 1.5 124 1.0

15 36 1.5

16 19 1.5 46 1.0

17 33 1.5 42 1.5

18 34 1.5 41 1.5 54 1.0

19 20 1.0 40 1.5

20

21 25 1.0 31 1.5 67 1.5

22 29 1.5 32 1.5 54 1.0

23 24 1.0 33 1.5 37 1.5

24

25 26 1.0 27 1.0

26

27

28 29 1.5 46 1.0

29

30 32 1.5 95 1.0

31 43 1.5

32 83 1.0

33 38 1.0

34 35 1.0 66 1.5

35

36 44 1.5 49 1.0

37 52 1.5 108 1.0

38 39 1.0 81 1.0

39

40 118 1.0

41 48 1.5

42 52 1.5 72 1.0

43 49 1.0 62 1.5

44 45 1.0 64 1.5

45

46 47 1.0 82 1.0

47

48 57 1.5 72 1.0

49 50 1.0 51 1.0

50

51

52 53 1.0

53

54 55 1.0 56 1.0

55

56

57 58 1.0 66 1.5

58

59 60 1.0 61 1.0 128 1.0

60

61

62 63 1.0 65 1.5

63

64 86 1.0

65 67 1.5 190 1.0

66 185 1.0

67 68 1.0

68

69 70 1.0 71 1.0 127 1.0

70

71

72 73 1.0 74 1.0

73

74

75 76 1.0 77 1.0 126 1.0

76

77

78 79 1.0 80 1.0 125 1.0

79

80

81

82

83

84 86 1.0 87 1.0 88 1.0 168 1.0

85 86 1.0 89 1.0 90 1.0 169 1.0

86 91 1.0

87

88

89

90

91 92 1.0 93 1.0 170 1.0

92

93

94 95 1.0 97 1.0 98 1.0 174 1.0

95 96 1.0 99 1.0

96 100 1.0 101 1.0 175 1.0

97

98

99 102 1.0 103 1.0 176 1.0

100

101

102

103

104 108 1.0 110 1.0 111 1.0 171 1.0

105 108 1.0 112 1.0 113 1.0 172 1.0

106 107 1.0 108 1.0 109 1.0 173 1.0

107

108

109

110

111

112

113

114 118 1.0 120 1.0 121 1.0 177 1.0

115 118 1.0 122 1.0 123 1.0 178 1.0

116 117 1.0 118 1.0 119 1.0 179 1.0

117

118

119

120

121

122

123

124

125

126

127

128

129 134 1.0 135 1.0 136 1.0

130 131 1.0 132 1.0 133 1.0

131

132

133

134

135

136

137 138 1.0 154 1.0 165 1.0 166 1.0

138 139 1.0 140 1.0 167 1.0

139 146 1.0 163 1.0 164 1.0

140

141 142 1.5 146 1.5 147 1.0

142 143 1.5 148 1.0

143 144 1.5 149 1.0

144 145 1.5 150 1.0

145 146 1.5 151 1.0

146

147

148

149

150

151

152 153 1.5 157 1.5 158 1.0

153 154 1.5 159 1.0

154 155 1.5

155 156 1.5 160 1.0

156 157 1.5 161 1.0

157 162 1.0

158

159

160

161

162

163

164

165

166

167

168

169

170

171

172

173

174

175

176

177

178

179

180 181 1.5 185 1.5 186 1.0

181 182 1.5 199 1.0

182 183 1.5 200 1.0

183 184 1.5 201 1.0

184 185 1.5 187 1.0

185

186

187

188 189 1.5 193 1.5 198 1.0

189 190 1.5 194 1.0

190 191 1.5

191 192 1.5 195 1.0

192 193 1.5 196 1.0

193 197 1.0

194

195

196 222 1.0

197 218 1.0

198 214 1.0

199 206 1.0

200 202 1.0

201 210 1.0

202 203 1.0 204 1.0 205 1.0

203

204

205

206 207 1.0 208 1.0 209 1.0

207

208

209

210 211 1.0 212 1.0 213 1.0

211

212

213

214 215 1.0 216 1.0 217 1.0

215

216

217

218 219 1.0 220 1.0 221 1.0

219

220

221

222 223 1.0 224 1.0 225 1.0

223

224

225

___________________________

**0 imaginary frequency**

**Energy: -4685.4166 Hartree**

**3c^+^**⊂**2**

**Figure S57.** DFT-optimized structure of the **3c^+^**⊂**2** complex.

O	-2.10931700	1.36522500	-4.99862000

O	-4.67732300	1.19995700	-1.69269400

O	0.18618200	-1.52294400	-4.32496800

O	-0.24278500	-3.91157600	-1.32133100

O	-2.57862100	-3.83406600	1.35551400

O	-5.52838100	-0.99706800	1.86971700

C	-4.78513400	2.52530500	-1.28328600

C	0.37192400	-4.11485600	3.54223400

C	-5.32519800	2.81004400	-0.02298200

C	-1.39796000	3.97154200	-2.49626600

C	-0.46267900	-4.29062400	2.42863200

C	-5.49991200	4.15384700	0.33609900

C	-1.71710800	-3.66029100	2.43069600

C	-4.35219400	3.54892200	-2.14114500

C	1.04101600	-4.17333100	-0.82090700

C	1.76947600	0.29716100	-4.33965300

C	-4.55993400	4.87263300	-1.74166100

C	-4.50692400	1.18560700	1.78334000

C	-0.00648500	2.15872300	-4.11258400

C	2.45891800	-5.05294900	0.90215800

C	-5.67866400	1.69915500	0.94654500

C	-2.12314700	3.13216900	-3.34972500

C	-1.41226700	2.23071000	-4.16280400

C	-4.48571600	-0.14975200	2.22893600

C	0.66639400	3.03108000	-3.25085100

C	1.16878700	-4.77055800	0.44691700

C	3.04968900	0.76184400	-4.02924400

C	-2.11447300	-2.83430400	3.50083200

C	-0.04583100	-5.19357900	1.26982900

C	-5.14801500	5.20984600	-0.51250200

C	1.45299000	-1.03206700	-3.99962800

C	2.16079800	-3.86251400	-1.60822400

C	-3.41333200	-0.65733400	2.98821000

C	-1.25182700	-2.70907300	4.59227800

C	-3.64742900	3.23083500	-3.44695700

C	2.35854400	-1.86100900	-3.30953600

C	-3.44633000	-2.10486100	3.48241000

C	3.42905800	-4.17381500	-1.09638400

C	0.76414200	1.20649700	-5.03198500

C	3.60082300	-1.32157400	-2.96721800

C	-2.40791200	1.89732200	-6.29387800

C	-2.37863100	0.21808000	3.33264100

C	-3.45122200	2.02110100	2.15353800

C	-3.45316200	-4.95964800	1.50188900

C	2.00856100	-3.32365200	-3.02739100

C	-0.82670800	-5.01407400	-2.03705000

C	-5.83257200	0.75486700	-2.42069200

C	-5.38405100	6.68858000	-0.15132500

C	0.10700500	-2.15138000	-5.61256000

C	-6.62531900	-1.01247100	2.78927900

C	-2.37056800	-0.95134400	-2.43168600

N	-1.08847300	-1.15488100	-1.62021500

C	-1.00038800	-0.35915400	-0.32532400

C	0.40185900	-0.35149500	0.22779000

C	-3.40068100	-2.03352300	-2.24689100

H	1.33214600	-4.61581800	3.53982900

H	-1.95508300	4.65480100	-1.86901300

H	-4.25659200	5.66201600	-2.42409100

H	2.56175100	-5.55465900	1.85931700

H	-6.45880200	2.07004200	1.62421700

H	-6.11079400	0.84729100	0.41865900

H	3.33627600	1.75273300	-4.36688900

H	-0.90317900	-5.29503900	0.60955000

H	-1.58214300	-2.10674600	5.43389100

H	-4.01701800	2.29298800	-3.86242600

H	-4.16011500	-2.64605600	2.86254600

H	-3.84885700	-2.10755200	4.50413500

H	4.29086600	-3.98651600	-1.72544400

H	0.03656300	0.60786900	-5.57696900

H	1.30659700	1.80866700	-5.77142000

H	4.28920700	-1.92697000	-2.39083900

H	-1.49205300	2.14496100	-6.84406500

H	-2.95458400	1.12015200	-6.83182600

H	-1.56158900	-0.14742300	3.94458400

H	-3.44887000	3.04726200	1.80212600

H	-2.88790600	-5.89518200	1.59316100

H	-4.07259300	-4.99289000	0.60519800

H	2.64089900	-3.93984900	-3.68002300

H	0.98164100	-3.48040300	-3.34958700

H	-0.26113100	-5.23142600	-2.95013600

H	-0.84751400	-5.91413200	-1.41565400

H	-5.65046600	-0.28267300	-2.69944400

H	-6.73374100	0.81308700	-1.79947800

H	0.16815400	-6.18902900	1.67753900

H	-3.89641300	4.01595500	-4.17313500

H	1.75001100	2.98947500	-3.22716900

H	-5.93822700	4.36122600	1.30618100

H	-5.98929600	1.35874200	-3.32190800

H	-1.84502900	-4.72110400	-2.29205100

H	-4.09526800	-4.85524900	2.38484200

H	-3.03228700	2.79532100	-6.22527000

H	-0.92186100	-2.16452500	-1.43121800

H	-1.71284200	-0.80472200	0.36988800

H	-1.33993900	0.64888400	-0.57506600

H	-2.04902300	-0.88866700	-3.47047600

H	-2.77426800	0.01908100	-2.13788900

H	-0.30677800	-0.87562800	-2.22026800

C	-0.00062400	3.95740600	-2.43641900

C	3.61188800	-4.76272500	0.15829600

C	-0.00034400	-3.34364100	4.64772100

C	-2.38680200	1.56669800	2.94637500

C	3.98052500	-0.01984300	-3.32752300

C	1.14419900	-1.70212500	6.19282200

C	2.23867400	-3.90703100	5.75353600

C	0.87912400	-3.19863400	5.90455100

C	0.13969000	-3.82594700	7.11112300

C	-0.10900500	5.82627500	-0.68850000

C	0.80130400	4.93884100	-1.55872300

C	1.74864800	4.16357200	-0.61507900

C	1.63873100	5.86001700	-2.47856000

C	6.14355700	-4.68242500	-0.21060700

C	5.20813500	-4.50845700	2.09900700

C	5.07725900	-6.69083900	0.83552700

C	4.99830500	-5.15040700	0.70714700

C	-4.03822300	7.45107100	-0.16585300

C	-6.34024200	7.32059400	-1.19115400

C	-6.01433000	6.85616600	1.24404700

C	5.31396100	0.51877800	-2.95052600

C	-1.31670200	2.49844300	3.39248400

H	1.65777900	-1.22007700	5.35456600

H	0.21947600	-1.14807200	6.37787400

H	2.82463200	-3.50021100	4.92226400

H	2.12359500	-4.98473000	5.59843700

H	-0.05348200	-4.89037700	6.94324400

H	-0.82109400	-3.33695700	7.29743800

H	-0.77251100	6.45125000	-1.29444100

H	-0.72353100	5.23214500	-0.00446100

H	1.18907100	3.50961700	0.06052400

H	2.46133700	3.54279100	-1.16720700

H	0.99478100	6.43220800	-3.15400700

H	2.34076900	5.28873100	-3.09441900

H	4.31254100	-7.08287200	1.51291600

H	4.94234300	-7.17455500	-0.13742700

H	6.09281800	-5.14590700	-1.20148400

H	6.14867300	-3.59463600	-0.33365700

H	5.15923200	-3.41711200	2.04272600

H	4.45982800	-4.84537100	2.82282800

H	-6.99216600	6.36886100	1.31281200

H	-5.37296400	6.44994600	2.03371700

H	-3.33595100	7.02446500	0.55838900

H	-3.56179000	7.42193900	-1.15025900

H	-5.92968200	7.27233600	-2.20402300

H	-7.30729500	6.80768400	-1.19737400

H	1.77427000	-1.59402900	7.08202900

H	2.82679200	-3.77065700	6.66613000

H	0.74395000	-3.72978600	8.01964300

H	7.10416400	-4.96581500	0.22984600

H	6.19181900	-4.78336500	2.49408800

H	6.05471500	-6.98898300	1.22958000

H	0.50591600	6.49613600	-0.07984100

H	2.32537800	4.86456900	-0.00183400

H	2.21827600	6.57142800	-1.87997700

H	-4.19469800	8.50376800	0.09289100

H	-6.51707100	8.37522900	-0.95406700

H	-6.16256100	7.91945600	1.45552800

H	0.74100100	-3.04390900	-5.65572700

H	-0.93619600	-2.43765400	-5.75987900

H	0.40954200	-1.46173900	-6.40773000

H	-7.36905600	-1.69761000	2.37750700

H	-6.31265700	-1.36977900	3.77747900

H	-7.07190700	-0.01728000	2.89718900

C	1.40628700	0.41186000	-0.38394300

C	2.70454200	0.41143500	0.11923600

C	3.03910900	-0.33898000	1.25940600

C	2.02274100	-1.08484100	1.87556500

C	0.72654400	-1.09528100	1.36689200

H	1.17461700	1.02365400	-1.25200800

H	3.46620700	1.01399300	-0.36450700

H	2.25369600	-1.69195900	2.74379800

H	-0.03338000	-1.68976200	1.85928000

C	-4.62680200	-3.93448300	-3.14381100

C	-3.62411700	-2.97430800	-3.26222400

C	-4.21752800	-2.07761500	-1.10909700

C	-5.21852600	-3.03686600	-0.99008400

C	-5.44493300	-3.98702100	-2.00170000

H	-4.80322300	-4.62711100	-3.96076700

H	-3.02462900	-2.93745400	-4.16847300

H	-4.09855200	-1.34435300	-0.31993400

H	-5.82327800	-3.04417700	-0.08985100

C	6.41089900	-0.33172000	-2.77390700

C	7.64531400	0.16561900	-2.34413000

C	7.82067000	1.53793900	-2.11368100

C	6.73289600	2.40893300	-2.34605000

C	5.49287600	1.89907200	-2.73659100

O	8.63904000	-0.74195700	-2.08406600

O	9.04530100	2.00578800	-1.72072400

O	6.98955200	3.72992700	-2.13185500

C	9.11788800	2.55472400	-0.39602500

C	9.83841100	-0.62989900	-2.86584100

C	5.95611800	4.67302100	-2.37772800

H	6.34233300	-1.39616800	-2.96530000

H	4.65374700	2.57201800	-2.85218400

H	10.17489200	2.75438600	-0.21309000

H	8.54902200	3.48494200	-0.32339600

H	8.74746900	1.83580800	0.34343400

H	10.48440100	-1.44621700	-2.53848800

H	9.61376100	-0.75249800	-3.93233600

H	10.33407400	0.32861400	-2.70000100

H	6.39550500	5.65282800	-2.18848000

H	5.61046500	4.62669100	-3.41777300

H	5.10235300	4.52415300	-1.70461800

C	-0.66145800	4.70088600	4.19671100

C	-1.64594400	3.82096000	3.74394700

C	0.01538300	2.08307500	3.48615500

C	1.00578000	2.95828600	3.94557100

C	0.67801600	4.27072300	4.31298200

O	2.28326700	2.47226400	4.07633500

O	1.65308500	5.13564400	4.74571300

O	-0.89093000	5.98946600	4.58140100

C	-2.21895800	6.48646000	4.51454900

C	1.67826200	5.35344800	6.16251600

C	3.29513000	3.12378900	3.29505100

H	-2.68011500	4.13755600	3.70154800

H	0.31795900	1.08471500	3.19321400

H	-2.60671200	6.46286900	3.48796600

H	-2.89688400	5.92273000	5.16771800

H	-2.16869000	7.52124500	4.85518200

H	2.51241700	6.03167200	6.35127600

H	0.74786300	5.81665000	6.50473800

H	1.84492100	4.41194500	6.69864900

H	4.22460600	2.59417000	3.50807500

H	3.06988000	3.03225900	2.22528800

H	3.39007200	4.17726400	3.56582700

C	7.05370800	-0.48088300	2.79457700

C	5.97105900	-0.36775400	3.66976700

C	4.66827900	-0.31248300	3.17442800

C	4.42580000	-0.36304600	1.79068000

C	5.52459900	-0.46044600	0.92109100

C	6.82715800	-0.52566200	1.41728600

H	8.06607800	-0.53351000	3.18387500

H	6.14056000	-0.31748700	4.74135800

H	3.83364700	-0.18821900	3.85808400

H	5.36012100	-0.50869500	-0.15019500

H	7.65748100	-0.62732400	0.72405000

C	-8.55237500	-6.93824600	-1.62891100

C	-8.75355700	-5.64201900	-1.15145800

C	-7.74684600	-4.68596500	-1.27127300

C	-6.51792300	-5.00548700	-1.87320400

C	-6.33026600	-6.31393900	-2.34999800

C	-7.33656800	-7.27050300	-2.22843700

H	-9.33694200	-7.68273700	-1.53562700

H	-9.70017700	-5.37200200	-0.69299000

H	-7.92411700	-3.67352500	-0.92102900

H	-5.37991700	-6.59053600	-2.79700500

H	-7.16837500	-8.27817100	-2.59644500

1 23 1.0 41 1.0

2 7 1.0 47 1.0

3 31 1.0 49 1.0

4 15 1.0 46 1.0

5 13 1.0 44 1.0

6 24 1.0 50 1.0

7 9 1.5 14 1.5

8 11 1.5 56 1.0 100 1.5

9 12 1.5 21 1.0

10 22 1.5 57 1.0 98 1.5

11 13 1.5 29 1.0

12 30 1.5 87 1.0

13 28 1.5

14 17 1.5 35 1.0

15 26 1.5 32 1.5

16 27 1.5 31 1.5 39 1.0

17 30 1.5 58 1.0

18 21 1.0 24 1.5 43 1.5

19 23 1.5 25 1.5 39 1.0

20 26 1.5 59 1.0 99 1.5

21 60 1.0 61 1.0

22 23 1.5 35 1.0

23

24 33 1.5

25 86 1.0 98 1.5

26 29 1.0

27 62 1.0 102 1.5

28 34 1.5 37 1.0

29 63 1.0 84 1.0

30 48 1.0

31 36 1.5

32 38 1.5 45 1.0

33 37 1.0 42 1.5

34 64 1.0 100 1.5

35 65 1.0 85 1.0

36 40 1.5 45 1.0

37 66 1.0 67 1.0

38 68 1.0 99 1.5

39 69 1.0 70 1.0

40 71 1.0 102 1.5

41 72 1.0 73 1.0 91 1.0

42 74 1.0 101 1.5

43 75 1.0 101 1.5

44 76 1.0 77 1.0 90 1.0

45 78 1.0 79 1.0

46 80 1.0 81 1.0 89 1.0

47 82 1.0 83 1.0 88 1.0

48 115 1.0 116 1.0 117 1.0

49 156 1.0 157 1.0 158 1.0

50 159 1.0 160 1.0 161 1.0

51 52 1.0 55 1.0 95 1.0 96 1.0

52 53 1.0 92 1.0 97 1.0

53 54 1.0 93 1.0 94 1.0

54 162 1.5 166 1.5

55 172 1.5 173 1.5

56

57

58

59

60

61

62

63

64

65

66

67

68

69

70

71

72

73

74

75

76

77

78

79

80

81

82

83

84

85

86

87

88

89

90

91

92

93

94

95

96

97

98 108 1.0

99 114 1.0

100 105 1.0

101 119 1.0

102 118 1.0

103 105 1.0 120 1.0 121 1.0 144 1.0

104 105 1.0 122 1.0 123 1.0 145 1.0

105 106 1.0

106 124 1.0 125 1.0 146 1.0

107 108 1.0 126 1.0 127 1.0 150 1.0

108 109 1.0 110 1.0

109 128 1.0 129 1.0 151 1.0

110 130 1.0 131 1.0 152 1.0

111 114 1.0 134 1.0 135 1.0 147 1.0

112 114 1.0 136 1.0 137 1.0 148 1.0

113 114 1.0 132 1.0 133 1.0 149 1.0

114

115 140 1.0 141 1.0 153 1.0

116 142 1.0 143 1.0 154 1.0

117 138 1.0 139 1.0 155 1.0

118 180 1.5 184 1.5

119 203 1.5 204 1.5

120

121

122

123

124

125

126

127

128

129

130

131

132

133

134

135

136

137

138

139

140

141

142

143

144

145

146

147

148

149

150

151

152

153

154

155

156

157

158

159

160

161

162 163 1.5 167 1.0

163 164 1.5 168 1.0

164 165 1.5 227 1.0

165 166 1.5 169 1.0

166 170 1.0

167

168

169

170

171 172 1.5 175 1.5 176 1.0

172 177 1.0

173 174 1.5 178 1.0

174 175 1.5 179 1.0

175 238 1.0

176

177

178

179

180 181 1.5 191 1.0

181 182 1.5 185 1.0

182 183 1.5 186 1.0

183 184 1.5 187 1.0

184 192 1.0

185 189 1.0

186 188 1.0

187 190 1.0

188 193 1.0 194 1.0 195 1.0

189 196 1.0 197 1.0 198 1.0

190 199 1.0 200 1.0 201 1.0

191

192

193

194

195

196

197

198

199

200

201

202 203 1.5 206 1.5 209 1.0

203 213 1.0

204 205 1.5 214 1.0

205 206 1.5 207 1.0

206 208 1.0

207 212 1.0

208 211 1.0

209 210 1.0

210 215 1.0 216 1.0 217 1.0

211 218 1.0 219 1.0 220 1.0

212 221 1.0 222 1.0 223 1.0

213

214

215

216

217

218

219

220

221

222

223

224 225 1.5 229 1.5 230 1.0

225 226 1.5 231 1.0

226 227 1.5 232 1.0

227 228 1.5

228 229 1.5 233 1.0

229 234 1.0

230

231

232

233

234

235 236 1.5 240 1.5 241 1.0

236 237 1.5 242 1.0

237 238 1.5 243 1.0

238 239 1.5

239 240 1.5 244 1.0

240 245 1.0

241

242

243

244

245

___________________________

**0 imaginary frequency**

**Energy: -5147.5408 Hartree**

(*endo-benzyl*)- **3d^+^**⊂**2**

**Figure S58.** DFT-optimized structure of the **3d^+^**⊂**2** complex.

C	-0.94115800	-0.48667300	-1.18559700

N	-0.41000500	-0.94712300	-2.53593100

C	-1.40915000	-0.75850500	-3.64654500

H	0.45830300	-0.42796000	-2.76415400

H	-0.14994200	-1.94326600	-2.49492100

C	-1.43348800	-1.77556500	-7.42338900

C	1.03081900	-1.94141600	1.72609700

C	0.10736500	-1.81040000	0.68970600

C	0.07462000	-0.64313900	-0.08482300

C	0.98126800	0.38773500	0.19035100

C	1.91270900	0.25072500	1.21929400

C	1.93458600	-0.91118000	1.99146700

H	1.03903000	-2.84461300	2.32688600

H	-0.59983800	-2.60980900	0.49441000

H	0.94952200	1.30544600	-0.38788900

H	2.61482900	1.05344600	1.41923300

H	2.64985900	-1.01067200	2.80239900

H	-1.83783400	-1.07992600	-0.99773200

H	-1.23618300	0.55580000	-1.32305700

C	-0.90745600	-1.34665400	-4.96250300

H	-1.60010300	0.31493100	-3.71652900

H	-2.33141200	-1.24462600	-3.31957700

C	-1.91498600	-1.16037500	-6.10588100

H	0.04534400	-0.88176200	-5.23996500

H	-0.70415300	-2.41428400	-4.82209700

H	-2.87575900	-1.60902000	-5.82084800

H	-2.10494900	-0.08924500	-6.25051200

H	-2.16810400	-1.62663700	-8.21966900

H	-1.26859300	-2.85405600	-7.32288700

H	-0.49145400	-1.32384800	-7.75286700

O	-1.51900800	-3.98363100	-2.37902000

O	1.64176200	-3.02104800	-3.29002000

O	-5.31719600	-2.13868200	-2.26759000

O	-3.92436000	1.37354700	-2.95634800

O	-0.31052700	3.39940200	-4.04543000

O	1.92976700	0.75179200	-3.40843300

C	2.42688100	-3.28836100	-2.16101900

C	-1.47941500	4.25969800	-0.65848400

H	-2.41969000	4.44983600	-0.15846700

C	3.50260000	-2.44013700	-1.85641300

C	-0.21223800	-5.96001200	0.45074100

H	0.70238700	-6.47476500	0.71881500

C	-1.51581500	3.97835300	-2.02947400

C	4.27829500	-2.74492800	-0.73048000

C	-0.30304600	3.71327900	-2.68772100

C	2.11445300	-4.40095500	-1.36002200

C	-4.54787100	2.30514000	-2.12842800

C	-4.17065600	-2.64031400	-0.20394100

C	2.93517400	-4.66569000	-0.26222800

H	2.72277400	-5.54715800	0.33372100

C	3.96253800	0.11804800	-2.24268000

C	-2.57712000	-4.68662300	-0.32101400

C	-4.75595900	4.54485900	-1.27356300

H	-4.39035400	5.56577100	-1.26186000

C	3.88211300	-1.29828000	-2.79883200

H	4.86590700	-1.53539800	-3.22464300

H	3.17461900	-1.29938700	-3.62476200

C	-0.25192100	-5.29926500	-0.78618900

C	-1.44471000	-4.66200200	-1.15695600

C	-1.30644200	-6.00554500	1.31912400

C	3.02804500	1.09939400	-2.61131300

C	-2.48015400	-5.36200200	0.89827400

C	-4.06487600	3.61612000	-2.06777200

C	-3.76790600	-2.22291100	1.06680800

H	-3.20341800	-2.90588700	1.69195500

C	0.91808600	3.73682300	-1.99147200

C	-5.88414700	4.20005400	-0.52364200

C	-2.82016100	4.02825500	-2.82553000

H	-2.69370100	3.40523300	-3.71138500

C	4.02916100	-3.85802200	0.07760000

C	-4.93567400	-1.75411300	-0.98769700

C	-5.65840000	1.90415700	-1.36703600

C	3.16138200	2.44156000	-2.20903400

C	0.90135400	4.03925000	-0.62680900

H	1.85219900	4.07494100	-0.10266100

C	0.96113300	-5.33739200	-1.71022500

H	0.63513400	-5.12172400	-2.72488900

C	-5.28017800	-0.47562000	-0.51896200

C	2.23838100	3.53597900	-2.72404100

H	2.01603300	3.36470100	-3.77638100

H	2.79809600	4.47746900	-2.67492000

C	-6.30321100	2.86077200	-0.58124400

H	-7.17037700	2.54493100	-0.00764400

C	-3.88855500	-4.04467600	-0.73219000

H	-3.96553000	-4.00178700	-1.81793600

H	-4.69884200	-4.70404500	-0.39302800

C	-4.85345800	-0.09886900	0.75670800

H	-5.08760100	0.90022400	1.11001800

C	-1.97224100	-4.81089100	-3.46024100

H	-2.94205300	-5.26541800	-3.23318200

H	-2.07646400	-4.16640000	-4.33506900

C	4.23425400	2.77364400	-1.37909400

H	4.37307800	3.81215000	-1.09310500

C	-0.28476100	4.31197400	0.06906700

C	5.17766700	1.82220700	-0.96183600

C	-4.10628400	-0.95788100	1.57414400

C	5.02975500	0.50917300	-1.42527300

H	5.77588700	-0.22652300	-1.14521200

C	-0.27032300	4.53550400	-4.91647700

H	0.64172700	5.12449600	-4.76169100

H	-1.13884500	5.18718400	-4.77099400

C	-6.12420400	0.46225700	-1.37444300

H	-7.15983900	0.43366300	-1.01272200

H	-6.13761600	0.06792500	-2.39129000

C	-4.50418400	1.32855800	-4.26461300

H	-4.34329900	2.26913600	-4.80585800

H	-4.01778700	0.51316900	-4.80245600

C	2.11317700	-3.66568200	-4.48173200

H	3.12611300	-3.33407500	-4.73552100

H	2.11847400	-4.75538200	-4.36876800

H	-2.96264400	5.05672500	-3.18400400

H	1.34820300	-6.36300500	-1.70680400

H	-3.36487300	-5.39980100	1.52737500

C	0.48072500	3.60069200	2.37520400

C	-1.64487500	4.89212100	2.15868600

C	-0.23836600	4.69979800	1.56000400

H	-0.03922500	2.64138500	2.29196000

H	1.51245000	3.45413100	2.04197800

H	-2.24600600	3.97964700	2.08943500

H	-2.19121300	5.70339400	1.66654700

C	0.53706000	6.03142400	1.70667800

H	0.04742800	6.83490500	1.14699700

H	1.56414700	5.94599600	1.33916200

C	-1.61229300	-5.75071600	3.80841400

C	-1.27612900	-6.74381300	2.67043000

C	-2.32594600	-7.88077500	2.65613500

H	-0.88560600	-4.93269800	3.84989200

H	-2.60536200	-5.30840500	3.68731700

C	0.10153400	-7.36539500	2.96668700

H	-3.33876000	-7.49942000	2.49638600

H	-2.11116100	-8.60459200	1.86335100

H	0.89059500	-6.60688100	3.01003400

H	0.38317300	-8.11484100	2.21973700

C	-8.12204200	5.29896500	-0.16120600

C	-6.65046100	4.74730800	1.81461600

C	-6.04783600	6.62470900	0.27786800

H	-5.01829000	6.64067700	0.65066800

C	-6.66022700	5.21282200	0.33869500

H	-6.05128700	7.02789400	-0.73997700

H	-8.16105000	5.63526200	-1.20223900

H	-8.63151200	4.33259000	-0.10298200

H	-7.12134200	3.76668100	1.93461000

H	-5.62664900	4.67943300	2.19741700

C	5.61715100	-5.59923100	0.95074200

C	6.03357600	-3.19957900	1.52717700

C	4.09159500	-4.38869000	2.55226900

H	3.60444800	-3.44320700	2.80919200

C	4.93403100	-4.24583300	1.26349000

H	3.31390000	-5.15186900	2.45352500

H	4.88534200	-6.39833700	0.79876000

H	6.22938700	-5.53018700	0.04580200

H	6.71534100	-3.09894900	0.67635300

H	5.61388500	-2.21310400	1.74987100

H	5.11538700	-2.10114800	-0.49815500

H	1.42952800	-3.38801500	-5.28642200

H	-5.58136200	1.13310300	-4.22041200

H	-0.28093400	4.14803300	-5.93758800

H	-1.25477300	-5.60980000	-3.68002200

C	-6.59690800	-2.77521000	-2.34366300

C	2.11308500	0.94805200	-4.82225600

H	1.14918200	1.24811100	-5.23727300

H	2.84470800	1.73691200	-5.01268500

H	2.46391800	0.02553200	-5.30000300

H	-6.76427400	-3.01490000	-3.39581200

H	-6.62202600	-3.69932400	-1.75407100

H	-7.39582900	-2.11129200	-1.99305700

H	0.51437900	3.87891600	3.43402900

H	-1.55967300	5.15173300	3.21823500

H	0.58385900	6.33088300	2.75931100

H	-8.69038700	6.01127100	0.44648300

H	-7.20069900	5.45791200	2.44069200

H	-6.63287000	7.30667700	0.90232200

H	-1.59644400	-6.26435300	4.77546700

H	-2.31990900	-8.41388100	3.61273300

H	0.07645100	-7.86699000	3.93863400

H	6.26864800	-5.89743900	1.77920800

H	6.63360800	-3.50597800	2.38927300

H	4.73164400	-4.67902800	3.39200200

C	-4.55292100	0.23668800	3.73433100

C	-4.17771300	0.61907100	5.02375900

C	-2.93359200	0.21110400	5.55242400

C	-2.08051500	-0.56665000	4.75862000

C	-2.45753700	-0.93692800	3.46469700

C	-3.69528600	-0.54936600	2.94279600

H	-5.52498100	0.51404200	3.34733300

H	-1.75081200	-1.50988200	2.87691700

C	7.18604800	3.50158600	1.77966100

C	6.12153800	3.12242700	0.95522400

C	6.31377200	2.21184600	-0.08759900

C	7.59352500	1.66488100	-0.29202300

C	8.66021600	2.03555100	0.52956000

C	8.46524900	2.96778100	1.57280300

H	5.14526300	3.54622100	1.16254400

H	7.75533100	0.98429800	-1.11829000

O	9.93643900	1.57711500	0.39569300

O	9.50179700	3.31815100	2.39950600

O	6.94382200	4.44446000	2.74397600

O	-4.94849800	1.36461300	5.86620600

O	-2.54149800	0.60311600	6.80796700

O	-0.88148300	-1.03263600	5.24001400

C	-3.15177400	-0.12011600	7.88485100

H	-4.23621800	0.02552300	7.89045300

H	-2.91944100	-1.18926200	7.81671700

H	-2.72584500	0.28633800	8.80393200

C	-6.21432100	1.81215800	5.40370500

H	-6.64174600	2.39506100	6.22030200

H	-6.11607500	2.45052700	4.51660700

H	-6.88129000	0.97238800	5.17111400

C	0.10481300	-0.03813600	5.54071600

H	-0.25329200	0.66330200	6.29782200

H	0.97215500	-0.58065800	5.92290400

H	0.39214900	0.50765900	4.63287500

C	7.15118600	4.03909500	4.10393200

H	6.89207900	4.90471500	4.71612500

H	6.48873900	3.20324200	4.36263900

H	8.19011200	3.75393400	4.28211700

C	10.43042100	4.25803200	1.84095500

H	9.92473100	5.19351800	1.57558400

H	11.17004200	4.45247400	2.61946900

H	10.92866800	3.84069800	0.96076100

C	10.21206000	0.63556900	-0.63095800

H	9.62399100	-0.28197100	-0.50352900

H	10.01781300	1.05316000	-1.62695600

H	11.27244500	0.39766600	-0.54132400

1 2 1.0 9 1.0 18 1.0 19 1.0

2 3 1.0 4 1.0 5 1.0

3 20 1.0 21 1.0 22 1.0

4

5

6 23 1.0 28 1.0 29 1.0 30 1.0

7 8 1.5 12 1.5 13 1.0

8 9 1.5 14 1.0

9 10 1.5

10 11 1.5 15 1.0

11 12 1.5 16 1.0

12 17 1.0

13

14

15

16

17

18

19

20 23 1.0 24 1.0 25 1.0

21

22

23 26 1.0 27 1.0

24

25

26

27

28

29

30

31 59 1.0 89 1.0

32 37 1.0 108 1.0

33 71 1.0 159 1.0

34 47 1.0 105 1.0

35 45 1.0 99 1.0

36 61 1.0 160 1.0

37 40 1.5 46 1.5

38 39 1.0 43 1.5 94 1.5

39

40 44 1.5 55 1.0

41 42 1.0 58 1.5 60 1.5

42

43 45 1.5 68 1.0

44 70 1.5 154 1.0

45 66 1.5

46 49 1.5 76 1.0

47 63 1.5 72 1.5

48 64 1.5 71 1.5 84 1.0

49 50 1.0 70 1.5

50

51 55 1.0 61 1.5 97 1.5

52 59 1.5 62 1.5 84 1.0

53 54 1.0 63 1.5 67 1.5

54

55 56 1.0 57 1.0

56

57

58 59 1.5 76 1.0

59

60 62 1.5 125 1.0

61 73 1.5

62 113 1.0

63 68 1.0

64 65 1.0 96 1.5

65

66 74 1.5 79 1.0

67 82 1.5 138 1.0

68 69 1.0 111 1.0

69

70 148 1.0

71 78 1.5

72 82 1.5 102 1.0

73 79 1.0 92 1.5

74 75 1.0 94 1.5

75

76 77 1.0 112 1.0

77

78 87 1.5 102 1.0

79 80 1.0 81 1.0

80

81

82 83 1.0

83

84 85 1.0 86 1.0

85

86

87 88 1.0 96 1.5

88

89 90 1.0 91 1.0 158 1.0

90

91

92 93 1.0 95 1.5

93

94 116 1.0

95 97 1.5 189 1.0

96 184 1.0

97 98 1.0

98

99 100 1.0 101 1.0 157 1.0

100

101

102 103 1.0 104 1.0

103

104

105 106 1.0 107 1.0 156 1.0

106

107

108 109 1.0 110 1.0 155 1.0

109

110

111

112

113

114 116 1.0 117 1.0 118 1.0 167 1.0

115 116 1.0 119 1.0 120 1.0 168 1.0

116 121 1.0

117

118

119

120

121 122 1.0 123 1.0 169 1.0

122

123

124 125 1.0 127 1.0 128 1.0 173 1.0

125 126 1.0 129 1.0

126 130 1.0 131 1.0 174 1.0

127

128

129 132 1.0 133 1.0 175 1.0

130

131

132

133

134 138 1.0 140 1.0 141 1.0 170 1.0

135 138 1.0 142 1.0 143 1.0 171 1.0

136 137 1.0 138 1.0 139 1.0 172 1.0

137

138

139

140

141

142

143

144 148 1.0 150 1.0 151 1.0 176 1.0

145 148 1.0 152 1.0 153 1.0 177 1.0

146 147 1.0 148 1.0 149 1.0 178 1.0

147

148

149

150

151

152

153

154

155

156

157

158

159 164 1.0 165 1.0 166 1.0

160 161 1.0 162 1.0 163 1.0

161

162

163

164

165

166

167

168

169

170

171

172

173

174

175

176

177

178

179 180 1.5 184 1.5 185 1.0

180 181 1.5 198 1.0

181 182 1.5 199 1.0

182 183 1.5 200 1.0

183 184 1.5 186 1.0

184

185

186

187 188 1.5 192 1.5 197 1.0

188 189 1.5 193 1.0

189 190 1.5

190 191 1.5 194 1.0

191 192 1.5 195 1.0

192 196 1.0

193

194

195 221 1.0

196 217 1.0

197 213 1.0

198 205 1.0

199 201 1.0

200 209 1.0

201 202 1.0 203 1.0 204 1.0

202

203

204

205 206 1.0 207 1.0 208 1.0

206

207

208

209 210 1.0 211 1.0 212 1.0

210

211

212

213 214 1.0 215 1.0 216 1.0

214

215

216

217 218 1.0 219 1.0 220 1.0

218

219

220

221 222 1.0 223 1.0 224 1.0

222

223

224

**___________________________**

**0 imaginary frequency**

**Energy: -4572.3081 Hartree**

(*endo-butyl*)-**3d^+^**⊂**2**

**Figure S59.** DFT-optimized structure of the (*endo-butyl*)-**3d^+^**⊂**2** complex.

C	-0.45502300	-3.22401000	0.24694400

N	0.82239400	-2.41216000	0.16617900

C	0.55481800	-0.92922000	0.05612800

H	1.42410100	-2.60977700	0.98580000

H	1.39969500	-2.73676100	-0.63008900

C	2.82996300	2.22336600	-0.24575600

C	0.15593100	-6.66314000	1.76756400

C	-0.06192800	-5.28860500	1.64600100

C	-0.18977100	-4.70226100	0.37815800

C	-0.10087600	-5.51104600	-0.76428700

C	0.11698700	-6.88525800	-0.63873500

C	0.24614000	-7.46288200	0.62620400

H	0.24492300	-7.11078700	2.75301700

H	-0.14236500	-4.66950300	2.53703000

H	-0.21110600	-5.06580400	-1.75072000

H	0.17587600	-7.50602400	-1.52789700

H	0.40687200	-8.53234600	0.72235600

H	-1.02846800	-3.00911900	-0.65567300

H	-1.01897900	-2.85117500	1.10331100

C	1.83619000	-0.10935000	-0.03961400

H	-0.02775700	-0.65603800	0.93983500

H	-0.07101900	-0.79450000	-0.82988500

C	1.54645000	1.39448500	-0.14614600

H	2.45831000	-0.29650900	0.84201700

H	2.41225800	-0.43023400	-0.91400000

H	0.92026700	1.58637400	-1.02380000

H	0.96956900	1.71988100	0.72579600

H	3.41645500	1.94326000	-1.12774200

H	2.60006000	3.28988600	-0.32282500

H	3.46536700	2.07709500	0.63500600

O	3.04433600	-3.12101600	1.92076200

O	2.98984500	-3.42039300	-1.51421900

O	0.03348500	-2.93330400	4.61868500

O	-3.14638100	-3.20239200	2.09981600

O	-3.24397700	-3.61394700	-1.40251300

O	-0.06521900	-3.76335900	-4.09835100

C	3.86005300	-2.44473100	-2.01328400

C	-5.87619400	-1.03913700	-1.54677100

H	-6.68556300	-0.67605400	-0.92601900

C	3.59713300	-1.89802800	-3.28191100

C	5.86615200	-0.75666900	1.70604600

H	6.70766900	-0.56000500	1.05339000

C	-5.08013100	-2.07850700	-1.04303400

C	4.46007000	-0.91161300	-3.76278300

C	-4.04952100	-2.57355300	-1.85510200

C	4.94513300	-2.01522500	-1.23745800

C	-3.96829600	-2.11547600	2.38182500

C	1.25083800	-0.92326100	4.07020000

C	5.77806400	-1.02011500	-1.77124300

H	6.63169700	-0.70675800	-1.18324000

C	1.09916100	-1.65577300	-3.92441900

C	3.71741600	-1.33707200	3.39663700

C	-5.88105100	-0.74867400	1.88968200

H	-6.74927600	-0.54994600	1.26975900

C	2.42936700	-2.37863600	-4.12393100

H	2.71559400	-2.27534800	-5.17864800

H	2.25452400	-3.44108600	-3.95641400

C	5.00105600	-1.80603100	1.36094500

C	3.93553900	-2.08629400	2.22701100

C	5.69877000	0.01265500	2.86095600

C	-0.10791600	-2.37883500	-3.97950300

C	4.60965800	-0.30364600	3.68902300

C	-5.05778300	-1.82400000	1.54178000

C	1.22194700	0.42018300	3.69073100

H	2.16198500	0.92629400	3.50117700

C	-3.79970200	-2.02802400	-3.12779300

C	-5.65525200	0.05215800	3.01796700

C	-5.37538800	-2.68842600	0.32489900

H	-4.81958800	-3.61883100	0.40902700

C	5.56305000	-0.44735100	-3.02792700

C	0.02573000	-1.58502000	4.27758800

C	-3.68814000	-1.32840500	3.50897500

C	-1.35316700	-1.73201400	-3.87117000

C	-4.63244700	-1.00494500	-3.58572500

H	-4.45679800	-0.61405900	-4.58435600

C	5.26641600	-2.64790700	0.11506400

H	4.71047500	-3.57818600	0.20156400

C	-1.20394700	-0.92084800	4.10862400

C	-2.65262900	-2.52791400	-3.98654500

H	-2.42760000	-3.56785200	-3.75185900

H	-2.97572000	-2.50505700	-5.03588800

C	-4.54223000	-0.25842300	3.80697000

H	-4.33382500	0.31936700	4.70083400

C	2.56656400	-1.65229700	4.33453200

H	2.35710100	-2.72162000	4.32950100

H	2.89104800	-1.40562600	5.35383100

C	-1.18198000	0.42716400	3.74182300

H	-2.12659800	0.95044500	3.64406100

C	3.42664400	-4.40465800	2.44368100

H	2.65109800	-5.10959500	2.14377000

H	4.39271800	-4.71961500	2.03559300

C	-1.36261400	-0.34464000	-3.72195200

H	-2.32032700	0.15757900	-3.66847300

C	-5.68309600	-0.48427900	-2.81449000

C	-0.18427200	0.41420100	-3.65994900

C	0.01757000	1.12553500	3.53459600

C	1.03820700	-0.27021200	-3.75284100

H	1.96828900	0.28237700	-3.67696500

C	-3.73651800	-4.91810900	-1.73033400

H	-3.00989700	-5.63279800	-1.34014900

H	-3.83266700	-5.04970100	-2.81489000

C	-2.51245600	-1.64579500	4.41474600

H	-2.30717900	-2.71545800	4.39769700

H	-2.80293800	-1.39791200	5.44432800

C	-3.59403500	-4.43464700	2.67685600

H	-3.63867000	-4.37189000	3.77078600

H	-4.58692400	-4.71327500	2.30508500

C	3.36555900	-4.77183900	-1.82907900

H	3.40922100	-4.92582700	-2.91214400

H	4.34287900	-5.01497700	-1.39915500

H	-6.44253400	-2.93935200	0.35878000

H	6.33111000	-2.90959500	0.11290800

H	4.45455200	0.25613500	4.60703400

C	-7.65017500	1.09515800	-2.38354800

C	-7.28378300	0.12870900	-4.66078700

C	-6.57254800	0.63701500	-3.38401700

H	-8.33406900	0.28247600	-2.11811800

H	-7.20890500	1.48985800	-1.46206500

H	-7.91950100	-0.73420400	-4.43808900

H	-6.56968700	-0.17271000	-5.43283700

C	-5.69665900	1.86214400	-3.74089500

H	-4.94249900	1.61936600	-4.49542000

H	-5.17596200	2.24753900	-2.85763700

C	7.78769500	1.34782800	2.22638300

C	6.66242800	1.14654900	3.25865700

C	5.87686500	2.47395100	3.37963400

H	8.41279800	0.45498200	2.12221000

H	7.39405400	1.61113300	1.23886700

C	7.31262400	0.81027000	4.62222700

H	5.41385700	2.74509400	2.42490300

H	5.08548700	2.41600000	4.13266200

H	6.56711600	0.70226700	5.41546900

H	7.88105300	-0.12367400	4.56685500

C	-6.18892100	1.99067100	4.60158800

C	-6.74585300	2.16731000	2.17212000

C	-8.03069100	0.58554200	3.65837200

H	-8.42569000	0.03673900	2.79829900

C	-6.63600100	1.18870800	3.36475600

H	-7.98764500	-0.10655600	4.50534600

H	-6.14393600	1.36671700	5.49986100

H	-5.20671200	2.45330600	4.45667600

H	-5.77178800	2.60844900	1.93564100

H	-7.11638600	1.67379000	1.26889600

C	5.67352000	1.89051300	-3.97130800

C	7.62271200	1.02727200	-2.66621500

C	7.13587900	0.06670000	-4.92420300

H	7.72880600	-0.82940000	-4.71464500

C	6.49235100	0.62276800	-3.63108300

H	6.38366400	-0.19710000	-5.67351500

H	4.87882700	1.68468300	-4.69441400

H	6.32453900	2.65485000	-4.40848100

H	8.27538600	0.18289200	-2.42152500

H	8.24579200	1.79596100	-3.13292100

H	4.26980000	-0.51006500	-4.75409700

H	2.60098200	-5.41848600	-1.39795900

H	-2.86990200	-5.19691700	2.38515100

H	-4.71445600	-5.10299700	-1.27038400

H	3.49747400	-4.37849000	3.53597800

C	0.03741000	-3.19667900	6.02699200

C	-0.12028600	-4.25476400	-5.44302600

H	-1.05210700	-3.96035300	-5.93874800

H	-0.07481600	-5.34362000	-5.37881200

H	0.72738600	-3.89054400	-6.03499200

H	0.04825500	-4.28239700	6.14091600

H	0.92544700	-2.77249600	6.50965300

H	-0.85769900	-2.79088600	6.51166200

H	-8.25026700	1.89427300	-2.82933700

H	-7.91691200	0.91633100	-5.08342200

H	-6.31956400	2.66732800	-4.14569600

H	-6.90549500	2.79387400	4.79838100

H	-7.43827000	2.98151700	2.41108000

H	-8.74518200	1.37863100	3.90423500

H	8.43874300	2.16598900	2.54838100

H	6.55005400	3.28679200	3.67181000

H	8.00042500	1.60839600	4.92082600

H	5.21006800	2.31566100	-3.07514900

H	7.23167800	1.44408200	-1.73193800

H	7.79924500	0.81559500	-5.36964700

C	1.09303100	3.39149500	3.53381800

C	1.12128500	4.73917400	3.16455700

C	0.06765000	5.29285400	2.42646100

C	-1.02630800	4.47465600	2.07359500

C	-1.03967700	3.12419100	2.42966500

C	0.02223900	2.56733900	3.16788100

H	1.90994600	3.02269300	4.14282000

H	-1.85682700	2.49214600	2.10715900

C	-1.41056600	3.88332700	-2.71773400

C	-1.35620100	2.50305900	-2.89708900

C	-0.23954800	1.89117100	-3.48999700

C	0.81107600	2.71575300	-3.92072200

C	0.78192700	4.09676200	-3.71591100

C	-0.32502700	4.69742000	-3.08778800

H	-2.19738800	1.92243800	-2.53678200

H	1.67868400	2.30980100	-4.42791400

O	1.88899500	4.81932500	-4.07591100

O	-0.34183600	6.05178600	-2.85845400

O	-2.51699200	4.40962800	-2.09043900

O	2.16393500	5.51020500	3.61235700

O	0.11521900	6.60515800	2.02187200

O	-2.01726400	5.10359400	1.38125400

C	-0.65470900	7.50814200	2.82947200

H	-1.71873000	7.25524900	2.79124700

H	-0.30392400	7.49430200	3.86727100

H	-0.50111400	8.50253200	2.40661200

C	3.02474400	6.05643000	2.60574700

H	2.48002400	6.72329500	1.93310100

H	3.79388600	6.61704100	3.14007300

H	3.50004700	5.25440600	2.02619200

C	-3.20008500	4.37133800	1.07981100

H	-3.88314400	5.08789500	0.62361900

H	-3.00364900	3.56814100	0.36328500

H	-3.65427500	3.96356300	1.99126800

C	-3.28853600	5.33367300	-2.87358100

H	-4.12111300	5.64521500	-2.24026800

H	-2.69425700	6.20451200	-3.15972300

H	-3.68163600	4.84135500	-3.77111700

C	-0.05522500	6.42419100	-1.49462000

H	-0.08869500	7.51432700	-1.46747100

H	-0.79740100	6.01513500	-0.80461300

H	0.94579600	6.08433800	-1.20852600

C	1.69188900	5.84909700	-5.05550000

H	2.67580600	6.28825800	-5.22890000

H	1.31725600	5.42182800	-5.99387800

H	1.00097400	6.61489900	-4.69644300

1 2 1.0 9 1.0 18 1.0 19 1.0

2 3 1.0 4 1.0 5 1.0

3 20 1.0 21 1.0 22 1.0

4

5

6 23 1.0 28 1.0 29 1.0 30 1.0

7 8 1.5 12 1.5 13 1.0

8 9 1.5 14 1.0

9 10 1.5

10 11 1.5 15 1.0

11 12 1.5 16 1.0

12 17 1.0

13

14

15

16

17

18

19

20 23 1.0 24 1.0 25 1.0

21

22

23 26 1.0 27 1.0

24

25

26

27

28

29

30

31 59 1.0 89 1.0

32 37 1.0 108 1.0

33 71 1.0 159 1.0

34 47 1.0 105 1.0

35 45 1.0 99 1.0

36 61 1.0 160 1.0

37 40 1.5 46 1.5

38 39 1.0 43 1.5 94 1.5

39

40 44 1.5 55 1.0

41 42 1.0 58 1.5 60 1.5

42

43 45 1.5 68 1.0

44 70 1.5 154 1.0

45 66 1.5

46 49 1.5 76 1.0

47 63 1.5 72 1.5

48 64 1.5 71 1.5 84 1.0

49 50 1.0 70 1.5

50

51 55 1.0 61 1.5 97 1.5

52 59 1.5 62 1.5 84 1.0

53 54 1.0 63 1.5 67 1.5

54

55 56 1.0 57 1.0

56

57

58 59 1.5 76 1.0

59

60 62 1.5 125 1.0

61 73 1.5

62 113 1.0

63 68 1.0

64 65 1.0 96 1.5

65

66 74 1.5 79 1.0

67 82 1.5 138 1.0

68 69 1.0 111 1.0

69

70 148 1.0

71 78 1.5

72 82 1.5 102 1.0

73 79 1.0 92 1.5

74 75 1.0 94 1.5

75

76 77 1.0 112 1.0

77

78 87 1.5 102 1.0

79 80 1.0 81 1.0

80

81

82 83 1.0

83

84 85 1.0 86 1.0

85

86

87 88 1.0 96 1.5

88

89 90 1.0 91 1.0 158 1.0

90

91

92 93 1.0 95 1.5

93

94 116 1.0

95 97 1.5 189 1.0

96 184 1.0

97 98 1.0

98

99 100 1.0 101 1.0 157 1.0

100

101

102 103 1.0 104 1.0

103

104

105 106 1.0 107 1.0 156 1.0

106

107

108 109 1.0 110 1.0 155 1.0

109

110

111

112

113

114 116 1.0 117 1.0 118 1.0 167 1.0

115 116 1.0 119 1.0 120 1.0 168 1.0

116 121 1.0

117

118

119

120

121 122 1.0 123 1.0 169 1.0

122

123

124 125 1.0 127 1.0 128 1.0 173 1.0

125 126 1.0 129 1.0

126 130 1.0 131 1.0 174 1.0

127

128

129 132 1.0 133 1.0 175 1.0

130

131

132

133

134 138 1.0 140 1.0 141 1.0 170 1.0

135 138 1.0 142 1.0 143 1.0 171 1.0

136 137 1.0 138 1.0 139 1.0 172 1.0

137

138

139

140

141

142

143

144 148 1.0 150 1.0 151 1.0 176 1.0

145 148 1.0 152 1.0 153 1.0 177 1.0

146 147 1.0 148 1.0 149 1.0 178 1.0

147

148

149

150

151

152

153

154

155

156

157

158

159 164 1.0 165 1.0 166 1.0

160 161 1.0 162 1.0 163 1.0

161

162

163

164

165

166

167

168

169

170

171

172

173

174

175

176

177

178

179 180 1.5 184 1.5 185 1.0

180 181 1.5 198 1.0

181 182 1.5 199 1.0

182 183 1.5 200 1.0

183 184 1.5 186 1.0

184

185

186

187 188 1.5 192 1.5 197 1.0

188 189 1.5 193 1.0

189 190 1.5

190 191 1.5 194 1.0

191 192 1.5 195 1.0

192 196 1.0

193

194

195 221 1.0

196 217 1.0

197 213 1.0

198 205 1.0

199 201 1.0

200 209 1.0

201 202 1.0 203 1.0 204 1.0

202

203

204

205 206 1.0 207 1.0 208 1.0

206

207

208

209 210 1.0 211 1.0 212 1.0

210

211

212

213 214 1.0 215 1.0 216 1.0

214

215

216

217 218 1.0 219 1.0 220 1.0

218

219

220

221 222 1.0 223 1.0 224 1.0

222

223

224

___________________________

**0 imaginary frequency**

**Energy: -4572.3219 Hartree**

(*endo-butyl*)-**3e^+^**⊂**2**

**Figure S60.** DFT-optimized structure of the (*endo-butyl*)-**3e^+^**⊂**2** complex.

O	2.62230100	2.60358000	-2.76613200

O	-0.73086500	3.17641100	-3.98657400

O	5.55834200	-0.56058600	-1.69264300

O	2.91008900	-3.56904500	-1.52305700

O	-0.59498400	-4.11769600	-2.74825900

O	-1.94087300	-0.17540800	-3.27561400

C	-1.11700000	3.63859300	-2.73067600

C	-0.83855000	-4.64496000	0.88474000

H	-0.28566300	-5.15490200	1.66592600

C	-2.33706700	3.21585500	-2.17977200

C	2.70974600	5.79523200	-0.92156100

H	2.14317300	6.72142800	-0.88922100

C	-0.33759300	-4.70821700	-0.41744100

C	-2.69376700	3.67893500	-0.90593500

C	-1.06281500	-4.06577000	-1.43638100

C	-0.26490100	4.50177400	-2.01818100

C	3.05846000	-4.18602600	-0.28440000

C	4.65325200	1.06386100	-0.16266900

C	-0.66496400	4.93425000	-0.75314200

H	-0.00442100	5.60483800	-0.21337400

C	-3.79837300	1.07460400	-2.36089300

C	4.16796700	3.42242000	-1.08796000

C	2.34321200	-5.79821500	1.35326300

H	1.64566900	-6.57729400	1.63983200

C	-3.29493400	2.35902600	-2.99843900

H	-4.17393700	2.97033700	-3.23602900

H	-2.80777500	2.13171300	-3.94425900

C	2.27478800	4.79657300	-1.79605200

C	3.02320000	3.60998000	-1.87146100

C	3.84784900	5.65497800	-0.11284700

C	-3.14167500	-0.15562700	-2.54621000

C	4.55545700	4.45465000	-0.21991900

C	2.13231100	-5.15421100	0.12495100

C	4.06570100	1.34247900	1.07293400

H	3.83987700	2.37231800	1.32652300

C	-2.23393400	-3.34192600	-1.15310600

C	3.42065700	-5.48994500	2.18979800

C	0.92774900	-5.50382000	-0.73271000

H	1.16586700	-5.35329100	-1.78429600

C	-1.88306100	4.55263900	-0.17180800

C	4.99218200	-0.27082600	-0.45559900

C	4.14052600	-3.82276700	0.53522000

C	-3.67272700	-1.35758600	-2.04643500

C	-2.68604800	-3.30220200	0.17192100

H	-3.58697500	-2.74339900	0.38768600

C	1.03143500	5.00830900	-2.63970400

H	1.15052300	4.52903600	-3.60958800

C	4.71953400	-1.31435300	0.44658900

C	-3.01315400	-2.70123700	-2.30237500

H	-2.33603600	-2.62228000	-3.15116100

H	-3.80428300	-3.40075200	-2.60540900

C	4.29812400	-4.48407000	1.75559100

H	5.15221000	-4.21542600	2.37148300

C	4.99977500	2.15977400	-1.16691900

H	4.94607000	1.72819500	-2.16725000

H	6.05081500	2.44154400	-1.02049900

C	4.12010900	-0.99035500	1.66742700

H	3.87876400	-1.79238600	2.35717800

C	3.15078700	2.78476900	-4.09265800

H	4.24074400	2.67694200	-4.09433500

H	2.70708300	2.01279500	-4.72173800

C	-4.88359700	-1.29624100	-1.34540100

H	-5.31576500	-2.21953600	-0.97167800

C	-2.01983400	-3.96516500	1.21045400

C	-5.55776900	-0.09048000	-1.11329700

C	3.80272200	0.33178200	2.01227800

C	-4.99446600	1.08160100	-1.63881800

H	-5.53524700	2.01717500	-1.53123800

C	-1.08145000	-5.23291800	-3.50421500

H	-2.17276500	-5.20297700	-3.60548900

H	-0.79556500	-6.18333000	-3.03955800

C	5.11972700	-2.74722600	0.10563200

H	6.08624900	-2.95657500	0.58221100

H	5.28634900	-2.79718100	-0.97067000

C	3.56110000	-4.27339800	-2.58863000

H	4.64027200	-4.35268700	-2.41313300

H	3.15354300	-5.28392400	-2.70725100

C	-1.13732700	4.01438600	-5.07635900

H	-2.22894400	4.10034400	-5.12916800

H	-0.71008400	5.02008800	-4.98961900

H	0.70858200	-6.57061800	-0.59960800

H	0.92843100	6.08389500	-2.82653100

H	5.45180800	4.30097300	0.37051600

C	-3.78386700	-3.09966500	2.85358800

C	-1.48844100	-3.64236400	3.67649900

C	-2.57474200	-4.03186800	2.64765600

H	-3.53327400	-2.05338700	2.64959900

H	-4.63105900	-3.37790600	2.21889500

H	-1.15448500	-2.60990300	3.53376100

H	-0.60827000	-4.28865500	3.61691900

C	-3.02963000	-5.48611800	2.92562000

H	-2.19761200	-6.19154400	2.83836100

H	-3.80635100	-5.79654600	2.21937100

C	4.57124800	8.06564900	-0.01527400

C	4.27492300	6.79982900	0.82391200

C	3.13056300	7.10093600	1.82114300

H	5.38280600	7.88431000	-0.72725000

H	3.69575400	8.39563300	-0.58227900

C	5.53808500	6.45138400	1.63349500

H	2.21380500	7.41002800	1.30981400

H	2.89708600	6.22202200	2.43096500

H	6.40032500	6.26259500	0.98565700

H	5.38647600	5.57415700	2.27100100

C	2.60106300	-7.26862300	3.83788100

C	5.05008900	-6.92183000	3.47080400

C	3.68198500	-5.17776200	4.67840200

H	4.46520500	-4.42505000	4.54433900

C	3.67667700	-6.21110300	3.52653300

H	2.72065000	-4.65717700	4.74324100

H	1.60260100	-6.82587200	3.91805400

H	2.56870100	-8.05480900	3.07680200

H	5.07349000	-7.66674000	2.66910000

H	5.86791700	-6.21664800	3.29540200

C	-2.49440500	6.67212000	1.02243500

C	-3.62077400	4.54720700	1.70880100

C	-1.19982300	4.88821100	2.24512400

H	-1.03576800	3.81882000	2.40832800

C	-2.29966300	5.14480700	1.18860800

H	-0.24096900	5.33109000	1.96161900

H	-1.57340900	7.16503800	0.69681800

H	-3.27123500	6.89144300	0.28296600

H	-4.45876000	4.75764900	1.03651100

H	-3.55398600	3.46336500	1.84763300

H	-3.64196200	3.35112900	-0.49655000

H	-0.76723300	3.54248800	-5.98897200

H	3.38307400	-3.70109500	-3.49949300

H	-0.62408500	-5.16446000	-4.49356200

H	2.89427000	3.77165800	-4.48825600

C	6.99145400	-0.56017700	-1.71627900

C	-2.12770300	-0.33661000	-4.69383200

H	-2.84595300	0.39081400	-5.08178700

H	-1.15680400	-0.17455900	-5.16321800

H	-2.48599100	-1.34478100	-4.92805100

H	7.28604800	-0.79606700	-2.74057000

H	7.39615500	0.41976800	-1.43884700

H	7.39990600	-1.31832800	-1.03888900

N	0.77079100	0.36676500	-2.09018200

C	1.62875400	-0.87226800	-2.26914400

H	-0.13385000	0.24392600	-2.58223200

C	3.23877700	-0.71098900	-4.21292300

C	3.57960200	-0.95099900	-5.54310100

C	2.66201500	-1.60507900	-6.36018300

C	1.43140900	-2.05048600	-5.88374200

C	1.10570800	-1.80966200	-4.55135800

C	1.99388300	-1.11904200	-3.71249800

H	3.96092900	-0.24668000	-3.54888300

H	4.53687800	-0.65209200	-5.95144500

H	0.76517200	-2.58354000	-6.55040800

H	0.17995600	-2.20077300	-4.14200100

H	1.06888400	-1.71626800	-1.87205100

H	2.51631700	-0.72900000	-1.65257800

H	-0.17703700	1.57860400	-0.67440200

H	1.25960600	1.18193200	-2.50236000

H	-4.12363800	-3.16220900	3.89186500

H	-1.88817400	-3.72639700	4.69251700

H	-3.43780400	-5.57177500	3.93857900

H	2.82400600	-7.74935300	4.79516500

H	5.25136100	-7.43577100	4.41687100

H	3.86321700	-5.67749100	5.63604500

H	4.87240200	8.89093100	0.63848400

H	3.42069500	7.91340200	2.49563400

H	5.79777000	7.29001900	2.28632400

H	-2.79543300	7.12268500	1.97433400

H	-3.86618600	4.98938600	2.67918100

H	-1.49129000	5.33410500	3.20219200

C	3.61864500	-0.05788800	4.48268800

C	3.09305300	0.26346200	5.73390300

C	2.16591300	1.32193300	5.87824700

C	1.74971700	2.01882800	4.73432900

C	2.28814300	1.69096700	3.48087400

C	3.22411500	0.66391200	3.34112000

H	4.36265700	-0.83777400	4.38891000

H	1.93667000	2.25696100	2.62566500

C	-9.00207800	-1.03005800	0.16791000

C	-7.79485200	-1.07062300	-0.53283000

C	-6.84041400	-0.05085700	-0.36593700

C	-7.11007600	0.99691700	0.51879800

C	-8.31881800	1.04641600	1.22079800

C	-9.27846600	0.03949500	1.04860400

H	-7.60355100	-1.86798600	-1.23968600

H	-6.38725400	1.78465900	0.69914100

O	-8.54690600	2.13120600	2.02630200

O	-10.44969300	0.07488800	1.76034800

O	-9.99316800	-1.95827900	0.05725300

O	3.42881500	-0.36447000	6.89563200

O	1.65583700	1.63006900	7.11588100

O	0.85689400	3.05245800	4.74052500

C	2.55944300	2.32048600	7.99023900

H	2.88402300	3.27012700	7.54774700

H	2.00096200	2.51869400	8.90689800

H	3.43175400	1.70312500	8.22144500

C	4.36117300	-1.43528100	6.84143800

H	4.46576400	-1.79144800	7.86674100

H	3.99578500	-2.25395200	6.20936200

H	5.33916500	-1.10200800	6.47233600

C	-0.26147500	3.02764400	5.63638100

H	-1.00547600	3.69006900	5.18911100

H	-0.67536800	2.01851700	5.72600900

H	0.00508600	3.38714800	6.63150600

C	-9.78825600	-3.06668700	-0.80690100

H	-9.66155000	-2.74953500	-1.84969900

H	-8.91681700	-3.65900900	-0.50113100

H	-10.68610900	-3.67983900	-0.72405800

C	-11.60317900	0.48416000	1.01201000

H	-11.81353900	-0.21860700	0.20035600

H	-12.43627700	0.48589400	1.71684800

H	-11.46557100	1.49312400	0.60660500

C	-8.72533600	1.86487000	3.42429900

H	-8.87290500	2.83758300	3.89689000

H	-9.59473300	1.22855100	3.60215400

H	-7.82862800	1.39183700	3.84434400

N	3.01382300	-1.85608400	-7.77222300

O	2.18614700	-2.44380400	-8.46526300

O	4.10891000	-1.45748000	-8.16204300

H	1.44048800	1.01948100	-0.20515500

C	0.48535900	0.71107100	-0.63826500

C	-0.12868900	-0.45310000	0.13524800

H	-0.97105000	-0.87190500	-0.42743700

H	0.61226400	-1.25104900	0.24284800

C	-0.61143700	-0.04815200	1.53924600

H	-0.78769200	-0.97341900	2.09617600

H	0.19314900	0.47264200	2.07181900

C	-1.89022900	0.79349800	1.55389600

H	-2.18965700	1.01887100	2.58244400

H	-1.76605900	1.74766200	1.03371100

H	-2.71881100	0.26223200	1.07303400

1 29 1.0 59 1.0

2 7 1.0 78 1.0

3 41 1.0 129 1.0

4 17 1.0 75 1.0

5 15 1.0 69 1.0

6 31 1.0 130 1.0

7 10 1.5 16 1.5

8 9 1.0 13 1.5 64 1.5

9

10 14 1.5 25 1.0

11 12 1.0 28 1.5 30 1.5

12

13 15 1.5 38 1.0

14 40 1.5 124 1.0

15 36 1.5

16 19 1.5 46 1.0

17 33 1.5 42 1.5

18 34 1.5 41 1.5 54 1.0

19 20 1.0 40 1.5

20

21 25 1.0 31 1.5 67 1.5

22 29 1.5 32 1.5 54 1.0

23 24 1.0 33 1.5 37 1.5

24

25 26 1.0 27 1.0

26

27

28 29 1.5 46 1.0

29

30 32 1.5 95 1.0

31 43 1.5

32 83 1.0

33 38 1.0

34 35 1.0 66 1.5

35

36 44 1.5 49 1.0

37 52 1.5 108 1.0

38 39 1.0 81 1.0

39

40 118 1.0

41 48 1.5

42 52 1.5 72 1.0

43 49 1.0 62 1.5

44 45 1.0 64 1.5

45

46 47 1.0 82 1.0

47

48 57 1.5 72 1.0

49 50 1.0 51 1.0

50

51

52 53 1.0

53

54 55 1.0 56 1.0

55

56

57 58 1.0 66 1.5

58

59 60 1.0 61 1.0 128 1.0

60

61

62 63 1.0 65 1.5

63

64 86 1.0

65 67 1.5 176 1.0

66 171 1.0

67 68 1.0

68

69 70 1.0 71 1.0 127 1.0

70

71

72 73 1.0 74 1.0

73

74

75 76 1.0 77 1.0 126 1.0

76

77

78 79 1.0 80 1.0 125 1.0

79

80

81

82

83

84 86 1.0 87 1.0 88 1.0 154 1.0

85 86 1.0 89 1.0 90 1.0 155 1.0

86 91 1.0

87

88

89

90

91 92 1.0 93 1.0 156 1.0

92

93

94 95 1.0 97 1.0 98 1.0 160 1.0

95 96 1.0 99 1.0

96 100 1.0 101 1.0 161 1.0

97

98

99 102 1.0 103 1.0 162 1.0

100

101

102

103

104 108 1.0 110 1.0 111 1.0 157 1.0

105 108 1.0 112 1.0 113 1.0 158 1.0

106 107 1.0 108 1.0 109 1.0 159 1.0

107

108

109

110

111

112

113

114 118 1.0 120 1.0 121 1.0 163 1.0

115 118 1.0 122 1.0 123 1.0 164 1.0

116 117 1.0 118 1.0 119 1.0 165 1.0

117

118

119

120

121

122

123

124

125

126

127

128

129 134 1.0 135 1.0 136 1.0

130 131 1.0 132 1.0 133 1.0

131

132

133

134

135

136

137 138 1.0 139 1.0 153 1.0 216 1.0

138 145 1.0 150 1.0 151 1.0

139

140 141 1.5 145 1.5 146 1.0

141 142 1.5 147 1.0

142 143 1.5 212 1.0

143 144 1.5 148 1.0

144 145 1.5 149 1.0

145

146

147

148

149

150

151

152 216 1.0

153

154

155

156

157

158

159

160

161

162

163

164

165

166 167 1.5 171 1.5 172 1.0

167 168 1.5 185 1.0

168 169 1.5 186 1.0

169 170 1.5 187 1.0

170 171 1.5 173 1.0

171

172

173

174 175 1.5 179 1.5 184 1.0

175 176 1.5 180 1.0

176 177 1.5

177 178 1.5 181 1.0

178 179 1.5 182 1.0

179 183 1.0

180

181

182 208 1.0

183 204 1.0

184 200 1.0

185 192 1.0

186 188 1.0

187 196 1.0

188 189 1.0 190 1.0 191 1.0

189

190

191

192 193 1.0 194 1.0 195 1.0

193

194

195

196 197 1.0 198 1.0 199 1.0

197

198

199

200 201 1.0 202 1.0 203 1.0

201

202

203

204 205 1.0 206 1.0 207 1.0

205

206

207

208 209 1.0 210 1.0 211 1.0

209

210

211

212 213 1.5 214 1.5

213

214

215 216 1.0

216 217 1.0

217 218 1.0 219 1.0 220 1.0

218

219

220 221 1.0 222 1.0 223 1.0

221

222

223 224 1.0 225 1.0 226 1.0

224

225

226

___________________________

**0 imaginary frequency**

**Energy: -4776.8096 Hartree**

(*endo-p-nitro-benzyl* )- **3e^+^**⊂**2**

**Figure S61.** DFT-optimized structure of the (*endo-p-nitro-benzyl* )- **3e^+^**⊂**2** complex.

O	-4.52811700	-0.00721700	-2.78478400

O	-4.73392900	-4.02291300	-0.45472700

O	-2.13033000	2.32072500	-4.58776600

O	0.77914200	0.40522900	-4.22734800

O	1.43387500	-3.40594000	-2.90306800

O	-0.91228400	-5.37358400	-1.00384300

C	-4.20262300	-3.10201600	0.43766300

C	4.71016100	-2.17977700	-1.77014200

H	5.42119700	-1.40156400	-2.02332800

C	-3.14015400	-3.47711200	1.28177800

C	-6.78928500	0.84076400	0.00752900

H	-7.32538200	0.31943800	0.79269400

C	3.47938900	-2.20048000	-2.43422300

C	-2.62405000	-2.51038900	2.14933100

H	-1.81414800	-2.78103700	2.81595400

C	2.61788200	-3.28513700	-2.17568100

C	-4.70752400	-1.78499700	0.42544300

C	1.80992000	1.04797400	-3.52761600

C	-3.11479500	3.22787100	-2.57222300

C	-4.16323500	-0.85788500	1.31795900

H	-4.52706500	0.16363700	1.30720300

C	-1.09154500	-5.04077100	1.37896600

C	-5.35749000	2.12565700	-2.01582600

C	3.86065900	0.99345900	-2.29377000

H	4.69836500	0.44938300	-1.87895600

C	-2.60399700	-4.91156100	1.27635900

H	-3.05325900	-5.44883300	2.12191200

H	-2.95902600	-5.39528700	0.36712200

C	-5.96512500	0.07199100	-0.82953000

C	-5.28664200	0.73128500	-1.86208100

C	-6.94565100	2.22176900	-0.14483800

C	-0.29155900	-5.22424100	0.23295900

C	-6.19941200	2.83991700	-1.16077700

H	-6.28020300	3.91320400	-1.30771700

C	2.91290500	0.30752900	-3.06006000

C	-2.92988200	3.85099400	-1.33672000

H	-3.80143900	4.00671500	-0.71010400

C	2.93317300	-4.26599900	-1.22243800

C	3.76395900	2.36492500	-2.02065500

C	3.10639600	-1.15371100	-3.48063600

H	2.18699400	-1.47984300	-3.96180800

C	-3.12283300	-1.20256600	2.19493700

C	-1.98286600	3.00512800	-3.38254200

C	1.67487400	2.42750700	-3.28797800

C	1.10939500	-5.25646400	0.32223300

C	4.17037900	-4.17512200	-0.56966100

H	4.42923400	-4.95536200	0.13608300

C	-5.81104600	-1.41409300	-0.56964300

H	-5.61808800	-1.95596400	-1.49781400

C	-0.70449700	3.41242800	-2.96842800

C	1.98413200	-5.42348500	-0.92139200

H	1.32914600	-5.58104400	-1.77592400

H	2.58734500	-6.33091200	-0.79758100

C	2.67016600	3.06678200	-2.54605900

H	2.61622900	4.14241300	-2.40643900

C	-0.57987800	4.05135700	-1.72581900

H	0.40919400	4.37216100	-1.42153100

C	-5.30519400	-0.39600500	-3.93305100

H	-5.72523700	0.47997000	-4.43758800

H	-4.63301500	-0.91967700	-4.61568400

C	1.69340500	-5.15997500	1.59467700

H	2.77338200	-5.19275100	1.66032200

C	5.08678700	-3.15173600	-0.83143700

C	0.93916800	-5.03235100	2.76472300

C	-1.67279100	4.28206200	-0.88458100

C	-0.45614200	-4.97173700	2.62124300

H	-1.07761300	-4.87697600	3.50717800

C	1.59947700	-4.14667000	-4.11793900

H	0.61850500	-4.19946000	-4.59384900

H	1.95879300	-5.16260700	-3.91848700

C	-7.88937300	3.06124900	0.73600200

C	1.57503600	-4.95266100	4.16559600

C	6.47933600	-3.08620000	-0.17393200

C	-7.08668100	4.18104100	1.43947100

H	-6.29687500	3.76126700	2.07199400

H	-7.74769700	4.77929400	2.07518500

H	-6.61585400	4.86182000	0.72410600

C	0.96271400	0.33159500	-5.64953200

H	1.14988200	1.32170700	-6.07690100

H	1.79876900	-0.32840900	-5.90224500

C	-8.58473100	2.21525700	1.81904100

H	-9.20620200	1.42539000	1.38530900

H	-9.23988500	2.85359700	2.41920400

H	-7.86451200	1.74978200	2.50040200

C	6.66913400	-1.72179500	0.53118300

H	7.66450400	-1.67372600	0.98650600

H	5.92719600	-1.57580000	1.32134900

H	6.58955300	-0.88277600	-0.16718800

C	-5.81498500	-4.80485600	0.06554200

H	-5.48880800	-5.42677100	0.90723800

H	-6.64569900	-4.17028200	0.39593400

C	-8.98157800	3.69899900	-0.15608400

H	-9.66110600	4.30596400	0.45142900

H	-9.57443700	2.92992400	-0.66130300

H	-8.55228900	4.34930500	-0.92398900

C	6.68030300	-4.19475200	0.87648000

H	5.94519500	-4.12508100	1.68464900

H	7.67330000	-4.09770100	1.32567500

H	6.61937900	-5.19530600	0.43566600

C	7.56067200	-3.25328800	-1.26876200

H	7.50180600	-2.46378900	-2.02437400

H	7.45737900	-4.21536700	-1.78118900

H	8.56066600	-3.21207800	-0.82371800

C	1.20571300	-3.60136300	4.82374900

H	0.12324000	-3.47790900	4.92734500

H	1.58948400	-2.75884100	4.24001100

H	1.64073600	-3.53983200	5.82713800

C	1.03978600	-6.11344300	5.03737900

H	-0.04729300	-6.07216700	5.15469600

H	1.48172700	-6.06885900	6.03842800

H	1.29271200	-7.08419800	4.59868000

C	3.11135600	-5.05473800	4.11628600

H	3.51335600	-5.00461800	5.13278500

H	3.55478500	-4.23140800	3.54769100

H	3.44395700	-6.00240100	3.67941700

H	3.88791900	-1.15760800	-4.25371300

C	-1.54907300	4.98253600	0.48181900

C	-2.41012700	6.26844400	0.47560600

C	-2.05236600	4.03295900	1.59514800

C	-0.09709900	5.38071500	0.80691100

H	0.31223600	6.08076000	0.07131300

H	0.56500100	4.50956300	0.85946300

H	-0.06202800	5.87423400	1.78248200

H	-2.33520800	6.77780000	1.44201100

H	-2.07498200	6.96364900	-0.30083700

H	-3.46746500	6.05099700	0.29735300

H	-1.98071800	4.51984200	2.57314800

H	-3.09717100	3.74484500	1.44551400

H	-1.45722900	3.11502100	1.63634100

H	-6.76962400	-1.79622900	-0.19572000

H	-6.15348300	-5.44799300	-0.74888100

H	-6.12091400	-1.06476700	-3.64028200

H	0.03889700	-0.07340000	-6.06743700

H	2.30791800	-3.65315000	-4.79422500

C	-1.17820600	-6.74286500	-1.33216900

H	-1.67008800	-6.74523100	-2.30613800

H	-0.25123700	-7.32569600	-1.39029900

H	-1.83850300	-7.20963100	-0.59139800

C	-2.42488000	3.15568100	-5.71631900

H	-2.51251300	2.49433000	-6.58070300

H	-3.36723600	3.69750600	-5.57762800

H	-1.62287300	3.88143800	-5.89291400

C	0.51705100	3.23208200	-3.86671300

H	0.90184400	4.22896600	-4.11228900

H	0.18779100	2.78219300	-4.80117900

C	-4.51540500	2.84500200	-3.05327100

H	-5.04092300	3.76048200	-3.35146000

H	-4.41243000	2.23389000	-3.94952400

C	-1.65367500	-0.18726200	-1.20781800

N	-1.60439500	-0.36462000	-2.71665200

H	-2.52509600	-0.03854200	-3.05082400

C	1.31443500	-1.63148600	0.69240100

C	0.14902400	-1.54568600	-0.06408200

C	-0.32826300	-0.30258200	-0.49797900

C	0.38898100	0.85900600	-0.17558200

C	1.55885700	0.79037700	0.57419700

C	1.99364200	-0.45900300	1.00639600

H	1.68424800	-2.58327300	1.04635300

H	-0.39808600	-2.45460000	-0.28678100

H	0.02038300	1.82595400	-0.49906800

H	2.12492200	1.67556000	0.83269700

H	-1.23437300	-1.61488800	-4.33146400

H	-2.36926500	-0.92000500	-0.83693700

H	-2.08079500	0.80549100	-1.05542700

H	-0.87972400	0.24046600	-3.13674400

C	6.77789500	4.40407900	0.28804500

C	7.13187900	3.35909200	-0.58449300

C	6.15536800	2.70146700	-1.33116300

C	4.80365200	3.05992500	-1.21983800

C	4.45210900	4.08026700	-0.32487000

C	5.42192900	4.75021700	0.42331400

O	7.73840600	5.07728900	1.00669600

O	8.44841000	3.02663200	-0.79053500

H	6.48151500	1.93781800	-2.02861000

H	3.41761300	4.37340600	-0.18175200

O	5.01439300	5.78664800	1.22188900

C	-1.36110900	1.67969300	4.87317100

C	-2.75930200	1.66460600	4.65252400

C	-3.33921900	0.73561800	3.78800500

C	-2.52958500	-0.20293700	3.12183300

C	-1.15025600	-0.17466800	3.32336200

C	-0.55635600	0.75946500	4.18453500

O	-0.80999000	2.61074400	5.72169300

O	-3.45692200	2.59846500	5.36241600

H	-4.41289700	0.70499600	3.65367200

H	-0.49390300	-0.85646500	2.79746800

O	0.79968900	0.65984400	4.29720100

C	-0.98956900	2.33557100	7.11762600

H	-0.48183900	3.13997100	7.65322300

H	-2.05007600	2.33314200	7.38485400

H	-0.53729800	1.37399000	7.38935900

C	-4.87131300	2.61544200	5.24637900

H	-5.21569100	3.41928300	5.89798400

H	-5.18856300	2.82521100	4.21679300

H	-5.31646400	1.66729000	5.57293300

C	1.60455400	1.82155000	4.53669700

H	2.60163400	1.55842700	4.17814400

H	1.22862800	2.68587300	3.98116000

H	1.64396000	2.07641400	5.59763700

C	8.44593700	6.07218400	0.24762500

H	7.75705400	6.84448300	-0.11174200

H	9.17013300	6.51857300	0.93110800

H	8.96875800	5.61566200	-0.59845200

C	9.12010200	2.43468800	0.33156500

H	9.13458900	3.11152800	1.18974000

H	8.63830800	1.49078100	0.61446400

H	10.14031400	2.23456300	-0.00010200

C	5.27887400	5.65351500	2.62559700

H	4.86180200	6.54680500	3.09390000

H	4.77798500	4.76491800	3.02960500

H	6.35070300	5.59433300	2.82661000

N	3.20889100	-0.54526600	1.83016700

O	3.54375200	-1.65602400	2.24367100

O	3.82815900	0.49476300	2.05116400

H	-0.39576800	-2.09935000	-2.85971500

C	-1.35415500	-1.75629200	-3.25325800

C	-2.48684100	-2.73829700	-2.96961400

H	-3.44897700	-2.26728700	-3.19403800

H	-2.51082700	-3.01305100	-1.91143900

C	-2.33594700	-4.01909900	-3.80435800

H	-2.17174100	-3.75626400	-4.85918200

H	-1.44161800	-4.55066400	-3.46338000

C	-3.56034400	-4.93243600	-3.69393900

H	-3.40991800	-5.86479400	-4.24730100

H	-3.77177900	-5.17557400	-2.64899800

H	-4.45268100	-4.44677300	-4.10478400

1 30 1.0 58 1.0

2 7 1.0 89 1.0

3 43 1.0 139 1.0

4 18 1.0 78 1.0

5 16 1.0 68 1.0

6 32 1.0 135 1.0

7 10 1.5 17 1.5

8 9 1.0 13 1.5 63 1.5

9

10 14 1.5 26 1.0

11 12 1.0 29 1.5 31 1.5

12

13 16 1.5 40 1.0

14 15 1.0 42 1.5

15

16 38 1.5

17 20 1.5 48 1.0

18 35 1.5 44 1.5

19 36 1.5 43 1.5 146 1.0

20 21 1.0 42 1.5

21

22 26 1.0 32 1.5 66 1.5

23 30 1.5 33 1.5 146 1.0

24 25 1.0 35 1.5 39 1.5

25

26 27 1.0 28 1.0

27

28

29 30 1.5 48 1.0

30

31 33 1.5 71 1.0

32 45 1.5

33 34 1.0

34

35 40 1.0

36 37 1.0 65 1.5

37

38 46 1.5 51 1.0

39 54 1.5 169 1.0

40 41 1.0 116 1.0

41

42 180 1.0

43 50 1.5

44 54 1.5 143 1.0

45 51 1.0 61 1.5

46 47 1.0 63 1.5

47

48 49 1.0 130 1.0

49

50 56 1.5 143 1.0

51 52 1.0 53 1.0

52

53

54 55 1.0

55

56 57 1.0 65 1.5

57

58 59 1.0 60 1.0 132 1.0

59

60

61 62 1.0 64 1.5

62

63 73 1.0

64 66 1.5 72 1.0

65 117 1.0

66 67 1.0

67

68 69 1.0 70 1.0 134 1.0

69

70

71 74 1.0 81 1.0 92 1.0

72 104 1.0 108 1.0 112 1.0

73 85 1.0 96 1.0 100 1.0

74 75 1.0 76 1.0 77 1.0

75

76

77

78 79 1.0 80 1.0 133 1.0

79

80

81 82 1.0 83 1.0 84 1.0

82

83

84

85 86 1.0 87 1.0 88 1.0

86

87

88

89 90 1.0 91 1.0 131 1.0

90

91

92 93 1.0 94 1.0 95 1.0

93

94

95

96 97 1.0 98 1.0 99 1.0

97

98

99

100 101 1.0 102 1.0 103 1.0

101

102

103

104 105 1.0 106 1.0 107 1.0

105

106

107

108 109 1.0 110 1.0 111 1.0

109

110

111

112 113 1.0 114 1.0 115 1.0

113

114

115

116

117 118 1.0 119 1.0 120 1.0

118 124 1.0 125 1.0 126 1.0

119 127 1.0 128 1.0 129 1.0

120 121 1.0 122 1.0 123 1.0

121

122

123

124

125

126

127

128

129

130

131

132

133

134

135 136 1.0 137 1.0 138 1.0

136

137

138

139 140 1.0 141 1.0 142 1.0

140

141

142

143 144 1.0 145 1.0

144

145

146 147 1.0 148 1.0

147

148

149 150 1.0 154 1.0 163 1.0 164 1.0

150 151 1.0 165 1.0 216 1.0

151

152 153 1.5 157 1.5 158 1.0

153 154 1.5 159 1.0

154 155 1.5

155 156 1.5 160 1.0

156 157 1.5 161 1.0

157 212 1.0

158

159

160

161

162 216 1.0

163

164

165

166 167 1.5 171 1.5 172 1.0

167 168 1.5 173 1.0

168 169 1.5 174 1.0

169 170 1.5

170 171 1.5 175 1.0

171 176 1.0

172 200 1.0

173 204 1.0

174

175

176 208 1.0

177 178 1.5 182 1.5 183 1.0

178 179 1.5 184 1.0

179 180 1.5 185 1.0

180 181 1.5

181 182 1.5 186 1.0

182 187 1.0

183 188 1.0

184 192 1.0

185

186

187 196 1.0

188 189 1.0 190 1.0 191 1.0

189

190

191

192 193 1.0 194 1.0 195 1.0

193

194

195

196 197 1.0 198 1.0 199 1.0

197

198

199

200 201 1.0 202 1.0 203 1.0

201

202

203

204 205 1.0 206 1.0 207 1.0

205

206

207

208 209 1.0 210 1.0 211 1.0

209

210

211

212 213 1.5 214 1.5

213

214

215 216 1.0

216 217 1.0

217 218 1.0 219 1.0 220 1.0

218

219

220 221 1.0 222 1.0 223 1.0

221

222

223 224 1.0 225 1.0 226 1.0

224

225

226

___________________________

**0 imaginary frequency**

**Energy: -4776.8022 Hartree**

(*eso-p-nitro-benzyl* )-**3f^+^**⊂**2**

**Figure S62.** DFT-optimized structure of the (*eso-p-nitro-benzyl* )-**3f^+^**⊂**2** complex.

O	-1.12585700	1.90316300	3.42507700

O	2.11605600	2.89966500	3.92159700

O	-3.38053600	-1.58320200	2.99396900

O	-1.41534100	-4.18885700	1.28199900

O	2.30448400	-4.62154800	1.08060800

O	3.40892800	-0.54898400	2.43973200

C	2.13464500	3.31518000	2.59427400

C	1.28324600	-3.54160200	-2.28603300

H	0.51841600	-3.78437900	-3.01643800

C	3.20476500	2.94154500	1.76497200

C	-2.34078500	5.12408200	2.12090200

H	-1.93925600	6.11641700	1.93649900

C	1.26602200	-4.22956100	-1.06866100

C	3.19156600	3.35865100	0.42705400

C	2.27302000	-3.93749700	-0.13163900

C	1.06666300	4.08142100	2.09344700

C	-1.99908000	-4.37119700	0.03216500

C	-4.38426800	0.30275400	1.87017100

C	1.10401500	4.47944500	0.75436300

H	0.28504200	5.09037100	0.38500000

C	4.92591000	1.05666700	1.45222400

C	-3.34362300	2.56926600	2.66962500

C	-1.82700600	-5.11251800	-2.25551700

H	-1.22758400	-5.56254500	-3.03915200

C	4.38268000	2.15883600	2.34020000

H	5.19992500	2.86209700	2.54407700

H	4.07257900	1.75316500	3.30287500

C	-1.47779600	4.15986900	2.64403700

C	-1.99364600	2.87378600	2.89777400

C	-3.70110800	4.87493000	1.88221100

C	4.41415900	-0.25026300	1.50535800

C	-4.17290200	3.59706200	2.18825200

C	-1.22407800	-4.91221700	-1.00654800

C	-5.14908600	0.77582300	0.80362300

H	-5.40593800	1.82565100	0.75892200

C	3.23179700	-2.93936300	-0.37594400

C	-3.16717900	-4.79368200	-2.50060700

C	0.22708900	-5.31215100	-0.78672300

H	0.36575300	-5.64745600	0.23994300

C	2.16153000	4.14374100	-0.10523000

C	-4.10608400	-1.07786300	1.91699100

C	-3.34460500	-4.01237100	-0.17211200

C	4.89721200	-1.26552000	0.66454600

C	3.19502200	-2.27017600	-1.60572300

H	3.94037500	-1.50784600	-1.79129700

C	-0.05332500	4.55207800	3.01003000

H	0.17116600	4.20817500	4.01935700

C	-4.52508900	-1.95528400	0.89930100

C	4.31327500	-2.66382600	0.67061200

H	3.89882700	-2.90047700	1.65119800

H	5.13652400	-3.37277900	0.51108900

C	-3.89746800	-4.24711200	-1.43540700

H	-4.94901300	-4.02282800	-1.58210700

C	-3.93682200	1.21113800	3.01892200

H	-3.22078300	0.65251400	3.61841600

H	-4.80819200	1.39099100	3.66375500

C	-5.29547600	-1.42810000	-0.14018300

H	-5.63077400	-2.08563500	-0.93417200

C	-1.03842300	1.93205500	4.86147100

H	-1.92416000	1.47139500	5.31424500

H	-0.14277800	1.37654700	5.14030800

C	5.94488100	-0.95144000	-0.20866100

H	6.32514000	-1.72965300	-0.86430900

C	2.24719500	-2.57082200	-2.59337100

C	6.50457500	0.33177600	-0.27756000

C	-5.63327100	-0.07073100	-0.20268600

C	5.97008300	1.32088900	0.56149800

H	6.40723000	2.31504200	0.54904000

C	3.09180100	-5.81899400	1.06857600

H	4.14023000	-5.60658500	0.83051100

H	2.70192100	-6.54124900	0.34254600

C	-4.19777700	-3.44734100	0.96053500

H	-5.14447000	-4.00179800	0.97570700

H	-3.69423500	-3.64042000	1.90466200

C	-1.56841300	-5.30690000	2.16439400

H	-2.62425200	-5.49948800	2.38797700

H	-1.13312700	-6.21760100	1.73678700

C	2.80148400	3.77431300	4.82761400

H	3.86370600	3.86393800	4.57356600

H	2.35417100	4.77521100	4.82985200

H	0.44131100	-6.17086100	-1.43517300

H	-0.01989700	5.64698400	3.04469100

H	-5.22965500	3.38575900	2.08505000

C	3.31785900	-0.79323400	-4.09287300

C	0.89181900	-1.32684100	-4.34328400

C	2.27414300	-1.92196600	-3.99165000

H	3.12153700	0.00911500	-3.37348100

H	4.33717200	-1.15691100	-3.93006800

H	0.60128100	-0.54774200	-3.63258100

H	0.10360300	-2.08531900	-4.34603400

C	2.63594000	-3.01237000	-5.02990700

H	1.90227300	-3.82425200	-5.03424500

H	3.61699600	-3.44895600	-4.81683600

C	-4.60717500	7.18101200	2.34213800

C	-4.60752200	5.99804500	1.34432000

C	-4.06664000	6.48053500	-0.02271800

H	-4.98896700	6.87014600	3.31990800

H	-3.60456200	7.59321600	2.48962200

C	-6.06336100	5.53507400	1.14698900

H	-3.04307400	6.85998800	0.05205600

H	-4.07365000	5.66840900	-0.75728200

H	-6.52251200	5.21375000	2.08766900

H	-6.14107200	4.71546900	0.42501500

C	-2.89166700	-5.64956000	-4.89677700

C	-5.03001700	-6.03346400	-3.66043000

C	-4.39553200	-3.71127200	-4.42019300

H	-5.11792900	-3.24253400	-3.74530800

C	-3.85431900	-5.04655200	-3.85639500

H	-3.58208700	-2.99732500	-4.58867200

H	-2.04304300	-4.98799400	-5.10061500

H	-2.50149800	-6.62164500	-4.57845000

H	-4.67664000	-6.99148400	-3.26605200

H	-5.78059000	-5.64328300	-2.96658200

C	2.29674500	6.22419000	-1.50343500

C	3.38155900	4.13589300	-2.35511500

C	0.88881700	4.28628100	-2.29144100

H	0.77477400	3.19919300	-2.34427100

C	2.18656400	4.68058900	-1.54974700

H	-0.00295600	4.69218100	-1.80490900

H	1.44834900	6.67543800	-0.98015800

H	3.21181000	6.53819500	-0.99126000

H	4.34055500	4.43878200	-1.92257900

H	3.36437100	3.04289300	-2.42383400

H	4.02254500	3.06348300	-0.20132600

H	2.70488000	3.33213300	5.82120500

H	-1.04267700	-5.05200100	3.08570000

H	3.02827900	-6.24514500	2.07169900

H	-0.95335500	2.95709100	5.22962500

C	-4.19670000	-2.02025500	4.08786400

C	3.93533900	-0.95851300	3.71712200

H	4.58421500	-0.18495800	4.13862400

H	3.08221500	-1.12333900	4.37551700

H	4.50576300	-1.88774200	3.61950700

H	-3.51781400	-2.40767200	4.85006800

H	-4.78134300	-1.19178400	4.50506400

H	-4.88452300	-2.81498200	3.77755700

C	0.79349700	0.24260600	0.45865800

N	0.57654300	-0.15070900	1.91922700

C	-0.08450300	-1.50325200	2.11607100

H	1.52784300	-0.17656100	2.32927300

C	-0.60301000	-1.51237500	4.59340000

C	-0.40378900	-1.98763100	5.88651500

C	0.52653000	-3.00512000	6.08517100

C	1.24779100	-3.56155400	5.03313200

C	1.04599000	-3.06758400	3.74446600

C	0.12750300	-2.03240700	3.51515200

H	-1.35325000	-0.75475900	4.40671900

H	-0.95543000	-1.59866700	6.73329500

H	1.94685100	-4.36381700	5.23396400

H	1.58463400	-3.50869000	2.91070900

C	-2.20277200	1.79296100	-1.28992200

C	-1.09273700	1.63318100	-0.46236700

C	-0.46088000	0.38651300	-0.35869200

C	-0.96468300	-0.69844500	-1.08943700

C	-2.08064000	-0.53849900	-1.90968200

C	-2.69533200	0.70931400	-2.01835000

H	-2.67638600	2.76646700	-1.37037700

H	-0.70139800	2.48269900	0.08769500

H	-0.47186000	-1.66419500	-1.03908600

H	-2.46324900	-1.38809100	-2.46547100

H	-3.56077300	0.83607100	-2.66034600

H	0.35061800	-2.17967300	1.38178300

H	-1.14408400	-1.38568400	1.89371500

H	1.44941800	-0.53015100	0.05156900

H	1.35004400	1.18077000	0.50203300

H	0.03014700	0.58507300	2.40329400

H	3.28599500	-0.35344100	-5.09424000

H	0.91989300	-0.88159000	-5.34343200

H	2.66693700	-2.58364100	-6.03741400

H	-3.42109400	-5.80325400	-5.84188500

H	-5.52944300	-6.22382800	-4.61642200

H	-4.89851500	-3.87894000	-5.37857900

H	-5.24590200	7.98922700	1.97072400

H	-4.69223500	7.29039000	-0.41227500

H	-6.66157100	6.36539200	0.76023600

H	2.31867600	6.63419800	-2.51882600

H	3.34668600	4.53057500	-3.37491200

H	0.90851100	4.67515300	-3.31496700

C	-7.53120500	-0.30627400	-1.83057300

C	-8.34029200	0.20147800	-2.84967400

C	-8.12183800	1.50342800	-3.35272400

C	-7.07047600	2.26629400	-2.82724000

C	-6.25938700	1.74719300	-1.81264400

C	-6.48335400	0.46601300	-1.29723000

H	-7.73353900	-1.28497900	-1.41470300

H	-5.44885000	2.36773900	-1.44806900

C	9.70365500	-0.01847600	-2.28265900

C	8.64588800	-0.30659900	-1.41771200

C	7.62585800	0.63769200	-1.20151300

C	7.67716600	1.86222700	-1.87325500

C	8.73539300	2.15940300	-2.73819000

C	9.76149100	1.22744300	-2.94632800

H	8.62541300	-1.24461000	-0.87756000

H	6.89401200	2.60340700	-1.76002400

O	8.75713200	3.40021400	-3.31853900

O	10.78228800	1.50940300	-3.81668800

O	10.74501100	-0.85840000	-2.53910000

O	-9.38640500	-0.46630800	-3.41186600

O	-8.89842300	2.00282900	-4.36708400

O	-6.83259600	3.55343200	-3.23667700

C	-10.20478400	2.44352000	-3.97087500

H	-10.13571500	3.23973900	-3.22072500

H	-10.67929600	2.83376600	-4.87281800

H	-10.79681000	1.61169000	-3.57801600

C	-9.67203500	-1.78167400	-2.95833600

H	-10.52139200	-2.12375300	-3.55041600

H	-8.82241100	-2.45637100	-3.12197000

H	-9.94323700	-1.79408700	-1.89526600

C	-6.47842500	3.73253400	-4.61470700

H	-6.31096800	4.80341800	-4.74369900

H	-5.55209900	3.19208500	-4.84867200

H	-7.27674800	3.39725600	-5.27979100

C	10.75614500	-2.13312600	-1.91265700

H	10.80227800	-2.04709800	-0.81971100

H	9.87589200	-2.72561700	-2.19165500

H	11.65514300	-2.63458500	-2.27220400

C	12.02894200	1.85912900	-3.19865600

H	12.41775100	1.02810800	-2.60276300

H	12.72040300	2.07683800	-4.01430000

H	11.91445700	2.74912600	-2.56933700

C	8.69917700	3.43973900	-4.75133600

H	8.70585200	4.49732200	-5.02090200

H	9.55725100	2.93519100	-5.20010200

H	7.76993200	2.98108700	-5.11236200

N	0.74391800	-3.51678900	7.45256500

O	1.57013300	-4.41527700	7.59622000

O	0.08656400	-3.00805800	8.35814800

1 29 1.0 59 1.0

2 7 1.0 78 1.0

3 41 1.0 129 1.0

4 17 1.0 75 1.0

5 15 1.0 69 1.0

6 31 1.0 130 1.0

7 10 1.5 16 1.5

8 9 1.0 13 1.5 64 1.5

9

10 14 1.5 25 1.0

11 12 1.0 28 1.5 30 1.5

12

13 15 1.5 38 1.0

14 40 1.5 124 1.0

15 36 1.5

16 19 1.5 46 1.0

17 33 1.5 42 1.5

18 34 1.5 41 1.5 54 1.0

19 20 1.0 40 1.5

20

21 25 1.0 31 1.5 67 1.5

22 29 1.5 32 1.5 54 1.0

23 24 1.0 33 1.5 37 1.5

24

25 26 1.0 27 1.0

26

27

28 29 1.5 46 1.0

29

30 32 1.5 95 1.0

31 43 1.5

32 83 1.0

33 38 1.0

34 35 1.0 66 1.5

35

36 44 1.5 49 1.0

37 52 1.5 108 1.0

38 39 1.0 81 1.0

39

40 118 1.0

41 48 1.5

42 52 1.5 72 1.0

43 49 1.0 62 1.5

44 45 1.0 64 1.5

45

46 47 1.0 82 1.0

47

48 57 1.5 72 1.0

49 50 1.0 51 1.0

50

51

52 53 1.0

53

54 55 1.0 56 1.0

55

56

57 58 1.0 66 1.5

58

59 60 1.0 61 1.0 128 1.0

60

61

62 63 1.0 65 1.5

63

64 86 1.0

65 67 1.5 189 1.0

66 184 1.0

67 68 1.0

68

69 70 1.0 71 1.0 127 1.0

70

71

72 73 1.0 74 1.0

73

74

75 76 1.0 77 1.0 126 1.0

76

77

78 79 1.0 80 1.0 125 1.0

79

80

81

82

83

84 86 1.0 87 1.0 88 1.0 167 1.0

85 86 1.0 89 1.0 90 1.0 168 1.0

86 91 1.0

87

88

89

90

91 92 1.0 93 1.0 169 1.0

92

93

94 95 1.0 97 1.0 98 1.0 173 1.0

95 96 1.0 99 1.0

96 100 1.0 101 1.0 174 1.0

97

98

99 102 1.0 103 1.0 175 1.0

100

101

102

103

104 108 1.0 110 1.0 111 1.0 170 1.0

105 108 1.0 112 1.0 113 1.0 171 1.0

106 107 1.0 108 1.0 109 1.0 172 1.0

107

108

109

110

111

112

113

114 118 1.0 120 1.0 121 1.0 176 1.0

115 118 1.0 122 1.0 123 1.0 177 1.0

116 117 1.0 118 1.0 119 1.0 178 1.0

117

118

119

120

121

122

123

124

125

126

127

128

129 134 1.0 135 1.0 136 1.0

130 131 1.0 132 1.0 133 1.0

131

132

133

134

135

136

137 138 1.0 153 1.0 164 1.0 165 1.0

138 139 1.0 140 1.0 166 1.0

139 146 1.0 162 1.0 163 1.0

140

141 142 1.5 146 1.5 147 1.0

142 143 1.5 148 1.0

143 144 1.5 225 1.0

144 145 1.5 149 1.0

145 146 1.5 150 1.0

146

147

148

149

150

151 152 1.5 156 1.5 157 1.0

152 153 1.5 158 1.0

153 154 1.5

154 155 1.5 159 1.0

155 156 1.5 160 1.0

156 161 1.0

157

158

159

160

161

162

163

164

165

166

167

168

169

170

171

172

173

174

175

176

177

178

179 180 1.5 184 1.5 185 1.0

180 181 1.5 198 1.0

181 182 1.5 199 1.0

182 183 1.5 200 1.0

183 184 1.5 186 1.0

184

185

186

187 188 1.5 192 1.5 197 1.0

188 189 1.5 193 1.0

189 190 1.5

190 191 1.5 194 1.0

191 192 1.5 195 1.0

192 196 1.0

193

194

195 221 1.0

196 217 1.0

197 213 1.0

198 205 1.0

199 201 1.0

200 209 1.0

201 202 1.0 203 1.0 204 1.0

202

203

204

205 206 1.0 207 1.0 208 1.0

206

207

208

209 210 1.0 211 1.0 212 1.0

210

211

212

213 214 1.0 215 1.0 216 1.0

214

215

216

217 218 1.0 219 1.0 220 1.0

218

219

220

221 222 1.0 223 1.0 224 1.0

222

223

224

225 226 1.5 227 1.5

226

227

___________________________

**0 imaginary frequency**

**Energy: -4889.9109 Hartree**

(*endo-p-nitro-benzyl* )-**3f^+^**⊂**2**

**Figure S63.** DFT-optimized structure of the (*endo-p-nitro-benzyl* )-**3f^+^**⊂**2** complex.

O	-3.88854600	-3.09532000	0.14072800

O	-1.64821600	-3.57970100	3.91996200

O	-3.07517000	-2.97167300	-3.19665400

O	0.61329300	-3.42171900	-4.34751600

O	2.82211100	-3.88332400	-0.62695500

O	2.00843400	-3.94051900	2.80293200

C	-1.58213900	-2.19248400	3.86718100

C	5.23793100	-1.66994700	-2.29858400

H	5.40493800	-1.25834300	-3.29014200

C	-0.40605500	-1.53767800	4.27495600

C	-5.66944200	-0.56560500	2.14854100

H	-5.73005900	-0.26386500	3.18806100

C	4.11679800	-2.48242400	-2.10808400

C	-0.37666100	-0.14215500	4.21077900

H	0.50583200	0.37628400	4.56489600

C	3.91956300	-3.05093600	-0.83679600

C	-2.68344500	-1.47504700	3.35304400

C	0.87950200	-2.07233000	-4.13268500

C	-4.58077000	-1.09771600	-2.90856500

C	-2.59439700	-0.08392600	3.28734100

H	-3.42156400	0.47603000	2.86676900

C	2.13539600	-2.00002000	4.23406000

C	-5.51060900	-1.42306200	-0.50931600

C	2.34623700	-0.33607900	-3.34024700

H	3.27659500	-0.04253200	-2.86916400

C	0.77686100	-2.32456000	4.84001500

H	0.83053400	-2.13122000	5.91899400

H	0.56236300	-3.38413300	4.71347400

C	-4.77782600	-1.59975400	1.82454500

C	-4.72785300	-2.02907400	0.48943700

C	-6.48157400	0.05757300	1.19609700

C	2.70490500	-2.81492900	3.23648000

C	-6.37425700	-0.39324500	-0.12820000

H	-7.00796400	0.04894000	-0.89087100

C	2.11191800	-1.69387200	-3.56021700

C	-4.92564200	0.21794400	-3.24875300

H	-5.79776300	0.64985100	-2.77401500

C	4.79715500	-2.78749700	0.22567600

C	1.41245500	0.65157000	-3.69589400

C	3.16613500	-2.76099500	-3.25895500

H	2.64486400	-3.70421700	-3.09573900

C	-1.45818500	0.60950800	3.73606900

C	-3.45305600	-1.66428100	-3.52145300

C	-0.10793200	-1.11965600	-4.44687300

C	3.94457600	-2.49941300	2.65845200

C	5.90579300	-1.96476400	-0.02329500

H	6.60359200	-1.79715100	0.78838800

C	-3.93277100	-2.24119000	2.91146200

H	-3.61155200	-3.23707200	2.60610700

C	-2.67951600	-0.94019400	-4.44979000

C	4.58574800	-3.40286600	1.60500400

H	3.97059000	-4.29377400	1.50448700

H	5.56405700	-3.72248000	1.98377800

C	0.18961700	0.22872700	-4.23188400

H	-0.54168600	0.97255700	-4.52138300

C	-3.09632600	0.35201100	-4.78000500

H	-2.53516000	0.89001500	-5.53798500

C	-4.51196700	-4.38575500	0.27902000

H	-4.83722100	-4.54941800	1.31094200

H	-5.38034600	-4.47214800	-0.38316100

C	4.61412100	-1.35380100	3.11263700

H	5.57297100	-1.11804200	2.66884300

C	6.15392300	-1.38911800	-1.27348300

C	4.10374900	-0.53122000	4.12051400

C	-4.21243400	0.96527700	-4.18987000

C	2.86111000	-0.88767400	4.66744100

H	2.44597500	-0.28887800	5.47275100

C	3.08775400	-5.27260600	-0.86043000

H	3.38337200	-5.45244000	-1.90075700

H	2.16183600	-5.80848300	-0.64522100

C	-7.47726000	1.17852800	1.54914800

C	4.84743300	0.71433400	4.63915200

C	7.37319000	-0.49162100	-1.55540400

C	-8.91119000	0.72494000	1.18471000

H	-9.01253100	0.50131400	0.11858400

H	-9.63054900	1.51423900	1.42690400

H	-9.19312700	-0.17299600	1.74373000

C	1.03659000	-3.92233700	-5.62232600

H	0.52627900	-3.40214600	-6.44112400

H	2.11888900	-3.81623000	-5.75330100

C	-7.12199600	2.45223400	0.74552800

H	-6.11187500	2.79965200	0.98673100

H	-7.82355600	3.25835500	0.98454900

H	-7.17042100	2.28216700	-0.33433400

C	6.88546000	0.89535700	-2.03881100

H	7.74031800	1.55651400	-2.21637800

H	6.23675800	1.36710300	-1.29393800

H	6.32712900	0.82661000	-2.97751200

C	-2.18172400	-4.10070200	5.14212400

H	-1.55413900	-3.82867500	5.99902100

H	-3.20099700	-3.74008200	5.32294200

C	-7.45214600	1.53424800	3.04759600

H	-8.17469700	2.33108400	3.24716500

H	-6.46986000	1.89759200	3.36742000

H	-7.72584700	0.68056900	3.67597600

C	8.24759600	-0.28315400	-0.30466000

H	8.65599000	-1.22667100	0.07225300

H	7.69301700	0.20203700	0.50529500

H	9.09499400	0.36252000	-0.55383900

C	8.24797400	-1.14164000	-2.65359800

H	8.61827800	-2.12096400	-2.33340800

H	9.11340500	-0.50752700	-2.87357100

H	7.69490300	-1.28154000	-3.58716900

C	3.98592600	1.97313200	4.37895400

H	3.01287700	1.91645900	4.87582100

H	3.81492800	2.11327900	3.30678500

H	4.49422600	2.86672600	4.75630700

C	5.10188400	0.56853500	6.15820000

H	4.17031800	0.47386300	6.72392300

H	5.63047100	1.44881000	6.53904900

H	5.71460700	-0.31334400	6.37155100

C	6.20589300	0.91611800	3.94109100

H	6.69666600	1.80635800	4.34580700

H	6.09006900	1.06848200	2.86292800

H	6.87951200	0.06806800	4.10323000

H	3.77085100	-2.89875500	-4.16566100

C	-4.61619200	2.39253400	-4.60625700

C	-3.44569400	3.36619200	-4.33087800

C	-4.94960300	2.40729800	-6.11745100

C	-5.85115400	2.89753100	-3.83636500

H	-6.09380800	3.91418200	-4.15944900

H	-6.73317800	2.27632000	-4.02381500

H	-5.67419300	2.93008000	-2.75593300

H	-3.72213400	4.38439600	-4.62324200

H	-3.18498800	3.38269700	-3.26758800

H	-2.54608300	3.10069800	-4.89328300

H	-5.23684400	3.41626000	-6.43148500

H	-4.09465600	2.10245900	-6.72812100

H	-5.78115200	1.73215800	-6.34363400

H	-4.57511200	-2.38436800	3.79103300

H	-2.19891700	-5.18747300	5.03727200

H	-3.76319000	-5.12968700	0.00840800

H	0.77282300	-4.98148100	-5.64424400

H	3.88335000	-5.64206800	-0.20383200

C	2.32768700	-5.13757400	3.51995300

H	1.74297100	-5.93987100	3.06536300

H	3.39427100	-5.37866700	3.44186900

H	2.06636700	-5.05161500	4.58152400

C	-3.66363100	-3.97229100	-4.04071100

H	-3.32385000	-4.93971000	-3.66676700

H	-4.75750000	-3.93106000	-3.99935400

H	-3.34536600	-3.85039000	-5.08172000

C	-1.43310200	-1.53695400	-5.08849700

H	-1.41428700	-1.23578700	-6.14335500

H	-1.48743100	-2.62419600	-5.06875800

C	-5.46620500	-1.90034700	-1.95599300

H	-6.48908500	-1.87250800	-2.34862700

H	-5.14478500	-2.93953800	-1.97749000

C	-1.11888200	-1.70325000	0.29886300

N	-1.28886500	-2.50203400	-0.98093900

C	-0.32447300	-3.66439100	-1.20355200

H	-2.25976000	-2.86034000	-0.98818300

C	-0.54552500	-5.14348500	0.86064100

C	-0.98704600	-6.32160400	1.46572800

C	-1.62205900	-7.31063400	0.71238700

C	-1.81832700	-7.11936700	-0.65681400

C	-1.39921800	-5.93233800	-1.25877400

C	-0.76781000	-4.92823000	-0.50850000

H	-0.00505400	-4.41686100	1.45933400

H	-0.82477000	-6.46549000	2.52919200

H	-2.29197200	-7.89238400	-1.25430300

H	-1.54218700	-5.78776000	-2.32619800

C	2.23778800	0.10123100	0.48799800

C	1.28248400	-0.91204700	0.51897300

C	-0.05739500	-0.63596800	0.21713100

C	-0.43487700	0.67590000	-0.10806900

C	0.50318900	1.70127500	-0.13086100

C	1.82856400	1.39216700	0.16659600

H	3.27480200	-0.09844500	0.72239400

H	1.58821600	-1.91414400	0.79744200

H	-1.47527700	0.90277100	-0.32191000

H	0.22989000	2.71816800	-0.37522700

H	0.66307300	-3.33578100	-0.87812400

H	-0.29307400	-3.81350400	-2.28293000

H	-0.92377200	-2.42088400	1.09336500

H	-2.09009900	-1.25256000	0.49628500

H	-1.21008300	-1.87111400	-1.78502000

H	-1.95095200	-8.23068500	1.18593700

C	2.27102100	4.86864200	-3.31889700

C	0.94292600	4.40517900	-3.29044000

C	0.67764100	3.03830700	-3.39043000

C	1.71051000	2.09926000	-3.53660700

C	3.03310500	2.56754700	-3.52620200

C	3.31362000	3.92679900	-3.39459600

O	2.53140100	6.21671100	-3.25438400

O	-0.13500800	5.25050500	-3.23840600

H	-0.36173000	2.73471600	-3.34571300

H	3.87592300	1.89687000	-3.64531900

O	4.62733000	4.32315600	-3.41834600

C	-1.30989900	4.91909800	3.87141000

C	-2.53808100	4.24280300	3.97624800

C	-2.57935800	2.84918600	3.91329300

C	-1.40975100	2.09467100	3.73800700

C	-0.19283600	2.77911600	3.59188200

C	-0.13641000	4.17084000	3.66472300

O	-1.26604700	6.29073600	3.97780400

O	-3.70609900	4.90800700	4.25091600

H	-3.54053300	2.37153900	4.06661900

H	0.73611900	2.24819000	3.41831500

O	1.09566600	4.77429600	3.62105000

C	-0.85563500	6.76070500	5.27320000

H	-0.86042200	7.85061600	5.21792000

H	-1.56020900	6.42952100	6.04371100

H	0.15208200	6.40715500	5.51263200

C	-4.15051000	5.84919100	3.26652700

H	-5.08607200	6.26237400	3.64766900

H	-3.42311500	6.65116300	3.12139800

H	-4.34234100	5.34687800	2.30925200

C	1.33047400	5.66591400	2.51908300

H	2.32801200	6.07916700	2.67675500

H	1.31893600	5.11524800	1.57205600

H	0.59430200	6.47332900	2.49699900

C	3.01150700	6.78804400	-4.48367500

H	3.16801100	7.84918200	-4.28312200

H	3.95439200	6.32449000	-4.78744800

H	2.26639400	6.67186000	-5.27853400

C	-0.20091400	6.16067700	-2.13240100

H	-1.15428100	6.68246000	-2.23386200

H	-0.18644700	5.61494000	-1.18031300

H	0.62209300	6.87707500	-2.15139300

C	5.10311500	4.93282500	-2.20336800

H	6.17389500	5.08578800	-2.34750700

H	4.61012300	5.89167900	-2.02239900

H	4.93274200	4.26995200	-1.34864600

N	2.82847000	2.47136700	0.14114200

O	2.43787900	3.61126700	-0.10860000

O	4.00312100	2.17399400	0.36692400

1 30 1.0 58 1.0

2 7 1.0 89 1.0

3 43 1.0 139 1.0

4 18 1.0 78 1.0

5 16 1.0 68 1.0

6 32 1.0 135 1.0

7 10 1.5 17 1.5

8 9 1.0 13 1.5 63 1.5

9

10 14 1.5 26 1.0

11 12 1.0 29 1.5 31 1.5

12

13 16 1.5 40 1.0

14 15 1.0 42 1.5

15

16 38 1.5

17 20 1.5 48 1.0

18 35 1.5 44 1.5

19 36 1.5 43 1.5 146 1.0

20 21 1.0 42 1.5

21

22 26 1.0 32 1.5 66 1.5

23 30 1.5 33 1.5 146 1.0

24 25 1.0 35 1.5 39 1.5

25

26 27 1.0 28 1.0

27

28

29 30 1.5 48 1.0

30

31 33 1.5 71 1.0

32 45 1.5

33 34 1.0

34

35 40 1.0

36 37 1.0 65 1.5

37

38 46 1.5 51 1.0

39 54 1.5 182 1.0

40 41 1.0 116 1.0

41

42 193 1.0

43 50 1.5

44 54 1.5 143 1.0

45 51 1.0 61 1.5

46 47 1.0 63 1.5

47

48 49 1.0 130 1.0

49

50 56 1.5 143 1.0

51 52 1.0 53 1.0

52

53

54 55 1.0

55

56 57 1.0 65 1.5

57

58 59 1.0 60 1.0 132 1.0

59

60

61 62 1.0 64 1.5

62

63 73 1.0

64 66 1.5 72 1.0

65 117 1.0

66 67 1.0

67

68 69 1.0 70 1.0 134 1.0

69

70

71 74 1.0 81 1.0 92 1.0

72 104 1.0 108 1.0 112 1.0

73 85 1.0 96 1.0 100 1.0

74 75 1.0 76 1.0 77 1.0

75

76

77

78 79 1.0 80 1.0 133 1.0

79

80

81 82 1.0 83 1.0 84 1.0

82

83

84

85 86 1.0 87 1.0 88 1.0

86

87

88

89 90 1.0 91 1.0 131 1.0

90

91

92 93 1.0 94 1.0 95 1.0

93

94

95

96 97 1.0 98 1.0 99 1.0

97

98

99

100 101 1.0 102 1.0 103 1.0

101

102

103

104 105 1.0 106 1.0 107 1.0

105

106

107

108 109 1.0 110 1.0 111 1.0

109

110

111

112 113 1.0 114 1.0 115 1.0

113

114

115

116

117 118 1.0 119 1.0 120 1.0

118 124 1.0 125 1.0 126 1.0

119 127 1.0 128 1.0 129 1.0

120 121 1.0 122 1.0 123 1.0

121

122

123

124

125

126

127

128

129

130

131

132

133

134

135 136 1.0 137 1.0 138 1.0

136

137

138

139 140 1.0 141 1.0 142 1.0

140

141

142

143 144 1.0 145 1.0

144

145

146 147 1.0 148 1.0

147

148

149 150 1.0 165 1.0 175 1.0 176 1.0

150 151 1.0 152 1.0 177 1.0

151 158 1.0 173 1.0 174 1.0

152

153 154 1.5 158 1.5 159 1.0

154 155 1.5 160 1.0

155 156 1.5 178 1.0

156 157 1.5 161 1.0

157 158 1.5 162 1.0

158

159

160

161

162

163 164 1.5 168 1.5 169 1.0

164 165 1.5 170 1.0

165 166 1.5

166 167 1.5 171 1.0

167 168 1.5 172 1.0

168 225 1.0

169

170

171

172

173

174

175

176

177

178

179 180 1.5 184 1.5 185 1.0

180 181 1.5 186 1.0

181 182 1.5 187 1.0

182 183 1.5

183 184 1.5 188 1.0

184 189 1.0

185 213 1.0

186 217 1.0

187

188

189 221 1.0

190 191 1.5 195 1.5 196 1.0

191 192 1.5 197 1.0

192 193 1.5 198 1.0

193 194 1.5

194 195 1.5 199 1.0

195 200 1.0

196 201 1.0

197 205 1.0

198

199

200 209 1.0

201 202 1.0 203 1.0 204 1.0

202

203

204

205 206 1.0 207 1.0 208 1.0

206

207

208

209 210 1.0 211 1.0 212 1.0

210

211

212

213 214 1.0 215 1.0 216 1.0

214

215

216

217 218 1.0 219 1.0 220 1.0

218

219

220

221 222 1.0 223 1.0 224 1.0

222

223

224

225 226 1.5 227 1.5

226

227

___________________________

**0 imaginary frequency**

**Energy: -4889.907 Hartree**

# NBO and NCI analysis

Natural Bond Orbital (NBO) analysis was carried out using version 3.1 of the NBO program,^4^ as integrated in Gaussian 16. In addition, second-order perturbation theory calculations were performed on the optimized structures at the b3lyp/6-31G(d,p) level of theory. Non-covalent interactions (NCI) were analyzed using the Multiwfn software,^5^ and the corresponding visualizations were generated with the ChemCraft V 1.8 program.^6^ The plots display (on the left) the Reduced Density Gradient (RDG) values as a function of the electron density multiplied by the sign of the second eigenvalue of the Hessian matrix (with s = 0.25 a.u.), and (on the right) the gradient isosurfaces (with s = 0.5 a.u.) for the studied complexes. A color-coding scheme was used to visually differentiate the types and strengths of the non-covalent interactions. Green regions indicate medium-to-strong interactions-such as cation∙∙∙π, van der Waals, and C–H···π contacts-whereas red regions highlight areas of repulsive interactions.

**a)**

**b)**

**c)**

**Figure S64:** a,b) Gradient RDG isosurfaces (0.25) for the noncovalent interaction (NCI) regions in (*endo-butyl*)- **3e^+^**⊂**1a** complex. c) Plot of RDG versus sign(I_2_)r for (*endo-butyl*)-**3e^+^**⊂**1a** complex (NCI-RDG isosurfaces with S= 0. 5).

**a)**

**b)**

**c)**

**Figure S65:** a,b) Gradient RDG isosurfaces (0.25) for the noncovalent interaction (NCI) regions in (*endo-p-nitro-benzyl*)-**3e^+^**⊂**1a** complex. c) Plot of RDG versus sign(I_2_)r for (*endo-p-nitro-benzyl*)-**3e^+^**⊂**1a** complex (NCI-RDG isosurfaces with S = 0. 5).

**a)**

**b)**

**c)**

**Figure S66:** a,b) Gradient RDG isosurfaces (0.25) for the noncovalent interaction (NCI) regions in (*endo-butyl*)-**3d^+^**⊂**1a** complex. c) Plot of RDG versus sign(I_2_)r for (*endo-butyl*)-**3d^+^**⊂**1a** complex (NCI-RDG isosurfaces with S = 0. 5).

**a)**

**b)**

**c)**

**Figure S67:** a,b) Gradient RDG isosurfaces (0.25) for the noncovalent interaction (NCI) regions in **3c^+^**⊂**1a** complex. c) Plot of RDG versus sign(I_2_)r for **3c^+^**⊂**1a** complex (NCI-RDG isosurfaces with S = 0. 5).

**a)**

**b)**

**c)**

**Figure S68:** a,b) Gradient RDG isosurfaces (0.25) for the noncovalent interaction (NCI) regions in **3b^+^**⊂**1a** complex. c) Plot of RDG versus sign(I_2_)r for **3b^+^**⊂**1a** complex (NCI-RDG isosurfaces with S = 0. 5).

**a)**

**b)**

**c)**

**Figure S69:** a,b) Gradient RDG isosurfaces (0.25) for the noncovalent interaction (NCI) regions in **3a^+^**⊂**1a** complex. c) Plot of RDG versus sign(I_2_)r for **3a^+^**⊂**1a** complex (NCI-RDG isosurfaces with S = 0. 5).

**a)**

**b)**

**c)**

**Figure S70:** a,b) Gradient RDG isosurfaces (0.25) for the noncovalent interaction (NCI) regions in (*endo-p-nitro-benzyl* )-**1a** complex. c) Plot of RDG versus sign(I_2_)r for (*endo-p-nitro-benzyl* )-**3f^+^**⊂**1a** complex (NCI-RDG isosurfaces with S = 0. 5).

**a)**

**b)**

**c)**

**Figure S71:** a,b) Gradient RDG isosurfaces (0.25) for the noncovalent interaction (NCI) regions in (*eso-p-nitro-benzyl* )-**3f^+^**⊂**1a** complex. c) Plot of RDG versus sign(I_2_)r for (*eso-p-nitro-benzyl* )-**1a** complex (NCI-RDG isosurfaces with S = 0. 5).

**a)**

**b)**

**c)**

**Figure S72:** a,b) Gradient RDG isosurfaces (0.25) for the noncovalent interaction (NCI) regions in (*endo-p-nitro-benzyl* )-**3e^+^**⊂**2** complex. c) Plot of RDG versus sign(I_2_)r for (*endo-p-nitro-benzyl* )- **3e^+^**⊂**2** complex (NCI-RDG isosurfaces with S = 0. 5).

**a)**

**b)**

**c)**

**Figure S73:** a,b) Gradient RDG isosurfaces (0.25) for the noncovalent interaction (NCI) regions in (*endo-butyl*)-**3e^+^**⊂**2** complex. c) Plot of RDG versus sign(I_2_)r for (*endo-butyl*)-**3e^+^**⊂**2** complex (NCI-RDG isosurfaces with S = 0. 5).

**a)**

**b)**

**c)**

**Figure S74:** a,b) Gradient RDG isosurfaces (0.25) for the noncovalent interaction (NCI) regions in (*endo-p-nitro-benzyl* )-**3f^+^**⊂**2** complex. c) Plot of RDG versus sign(I_2_)r for (*endo-p-nitro-benzyl* )-**3f^+^**⊂**2** complex (NCI-RDG isosurfaces with S = 0. 5).

**a)**

**b)**

**c)**

**Figure S75:** a,b) Gradient RDG isosurfaces (0.25) for the noncovalent interaction (NCI) regions in (*eso-p-nitro-benzyl* )-**3f^+^**⊂**2** complex. c) Plot of RDG versus sign(I_2_)r for (*eso-p-nitro-benzyl* )-**3f^+^**⊂**2** complex (NCI-RDG isosurfaces with S = 0. 5).

**a)**

**b)**

**c)**

**Figure S76:** a,b) Gradient RDG isosurfaces (0.25) for the noncovalent interaction (NCI) regions in (*endo-butyl*)-**3d^+^**⊂**2** complex. c) Plot of RDG versus sign(I_2_)r for (*endo-butyl*)-**3d^+^**⊂**2** complex (NCI-RDG isosurfaces with S = 0. 5).

**a)**

**b)**

**c)**

**Figure S77:** a,b) Gradient RDG isosurfaces (0.25) for the noncovalent interaction (NCI) regions in (*endo-benzyl*)- **3d^+^**⊂**2** complex. c) Plot of RDG versus sign(I_2_)r for (*endo-benzyl*)- **3d^+^**⊂**2** complex (NCI-RDG isosurfaces with S = 0. 5).

**a)**

**b)**

**c)**

**Figure S78:** a,b) Gradient RDG isosurfaces (0.25) for the noncovalent interaction (NCI) regions in **3b^+^**⊂**2** complex. c) Plot of RDG versus sign(I_2_)r for **3b^+^**⊂**2** complex (NCI-RDG isosurfaces with S = 0.5).

**a)**

**b)**

**c)**

**Figure S79:** a,b) Gradient RDG isosurfaces (0.25) for the noncovalent interaction (NCI) regions in **3c^+^**⊂**2** complex. c) Plot of RDG versus sign(I_2_)r for **3c^+^**⊂**2** complex (NCI-RDG isosurfaces with S = 0.5).

**a)**

**b)**

**c)**

**Figure S80:** a,b) Gradient RDG isosurfaces (0.25) for the noncovalent interaction (NCI) regions in **3a^+^**⊂**2** complex. c) Plot of RDG versus sign(I_2_)r for **3a^+^**⊂**2** complex (NCI-RDG isosurfaces with S = 0.5).

# Figure S81: DFT-optimized structure of the 3b^+^⊂2 complex. where it is evident that the benzyl unit of the axis 3b⁺ cannot interact with the 1,2,3-trimethoxybenzene portion of receptor 2.

# Figure S82: DFT-optimized structure of the 3b^+^⊂1a complex, where it is evident that the α*^out^*-CH₂–N⁺H₂–Bn group, protruding from the cavity of 1a, can form a stabilizing hydrogen bond interaction with the oxygen atom at the lower rim of calixarene 1a, represented as +N⁺C−H···O*^calix^*, with a C−H···O angle of 164° and a C−O distance of 3.3 Å.

# Figure S83: DFT-optimized structure of the 3c^+^⊂2 complex, where it is evident that one of the two hydrogen bonds has a highly unfavorable +N-H···O angle of 129°. In particular, the +N-H···O average angle is 148°.

**Figure S84**: DFT-optimized structure of the **3b^+^⊂2** complex, where it is evident that the +N-H···O average angle is 166°.

# References

**[1]** C. Gaeta, F. Troisi, P. Neri, “*endo* -Cavity Complexation and Through-the-Annulus Threading of Large Calixarenes Induced by Very Loose Alkylammonium Ion Pairs” *Org. Lett.* **2010**, *12*, 2092–2095.

**[2]** E. Krieger, G. Vriend, “YASARA View—molecular graphics for all devices—from smartphones to workstations” *Bioinformatics* **2014**, *30*, 2981–2982.

**[3]** Gaussian 16, Revision A.03, M. J. Frisch, G. W. Trucks, H. B. Schlegel, G. E. Scuseria, M. A. Robb, J. R. Cheeseman, G. Scalmani, V. Barone, G. A. Petersson, H. Nakatsuji, X. Li, M. Caricato, A. V. Marenich, J. Bloino, B. G. Janesko, R. Gomperts, B. Mennucci, H. P. Hratchian, J. V. Ortiz, A. F. Izmaylov, J. L. Sonnenberg, D. Williams-Young, F. Ding, F. Lipparini, F. Egidi, J. Goings, B. Peng, A. Petrone, T. Henderson, D. Ranasinghe, V. G. Zakrzewski, J. Gao, N. Rega, G. Zheng, W. Liang, M. Hada, M. Ehara, K. Toyota, R. Fukuda, J. Hasegawa, M. Ishida, T. Nakajima, Y. Honda, O. Kitao, H. Nakai, T. Vreven, K. Throssell, J. A. Montgomery, Jr., J. E. Peralta, F. Ogliaro, M. J. Bearpark, J. J. Heyd, E. N. Brothers, K. N. Kudin, V. N. Staroverov, T. A. Keith, R. Kobayashi, J. Normand, K. Raghavachari, A. P. Rendell, J. C. Burant, S. S. Iyengar, J. Tomasi, M. Cossi, J. M. Millam, M. Klene, C. Adamo, R. Cammi, J. W. Ochterski, R. L. Martin, K. Morokuma, O. Farkas, J. B. Foresman, and D. J. Fox, Gaussian, Inc., Wallingford CT, 2016.

**[4]** NBO Version 3.1, E. D. Glendening, A. E. Reed, J. E. Carpenter, F. Weinhold.

**[5]** T. Lu, F. W. Chen, *J. Comput. Chem.* **2012**, 33, 580-592.

**[6]** Chemcraft - graphical software for visualization of quantum chemistry computations. Version 1.8, build 682. https://www.chemcraftprog.com
